# Supplementary material for: Geographical distribution of antimicrobial exposure among very preterm and very low birth weight infants: A nationwide database study in Japan
Source: PLoS One. 2024 Jan 25;19(1):e0295528. doi: 10.1371/journal.pone.0295528 (PMC10810499; doi:10.1371/journal.pone.0295528)

J01AA08. Minocycline

Early Neonatal Exposure among Very Preterm and Very Low Birth Weight Infants (Days 0–6)

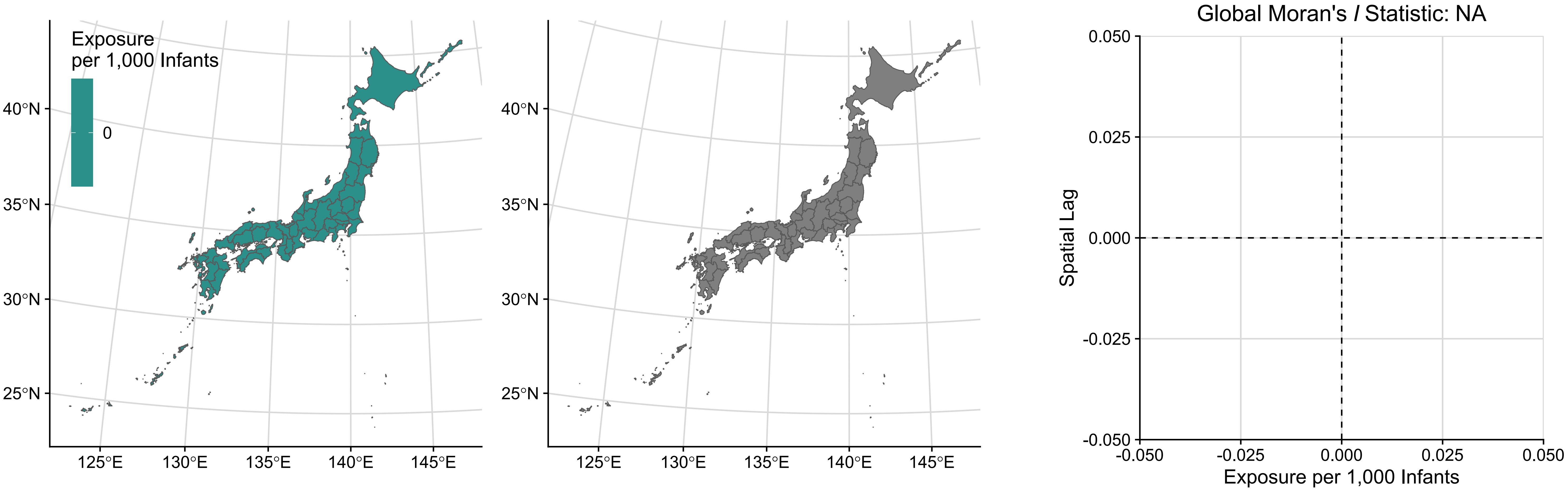

Neonatal Exposure among Very Preterm and Very Low Birth Weight Infants (Days 0–27)

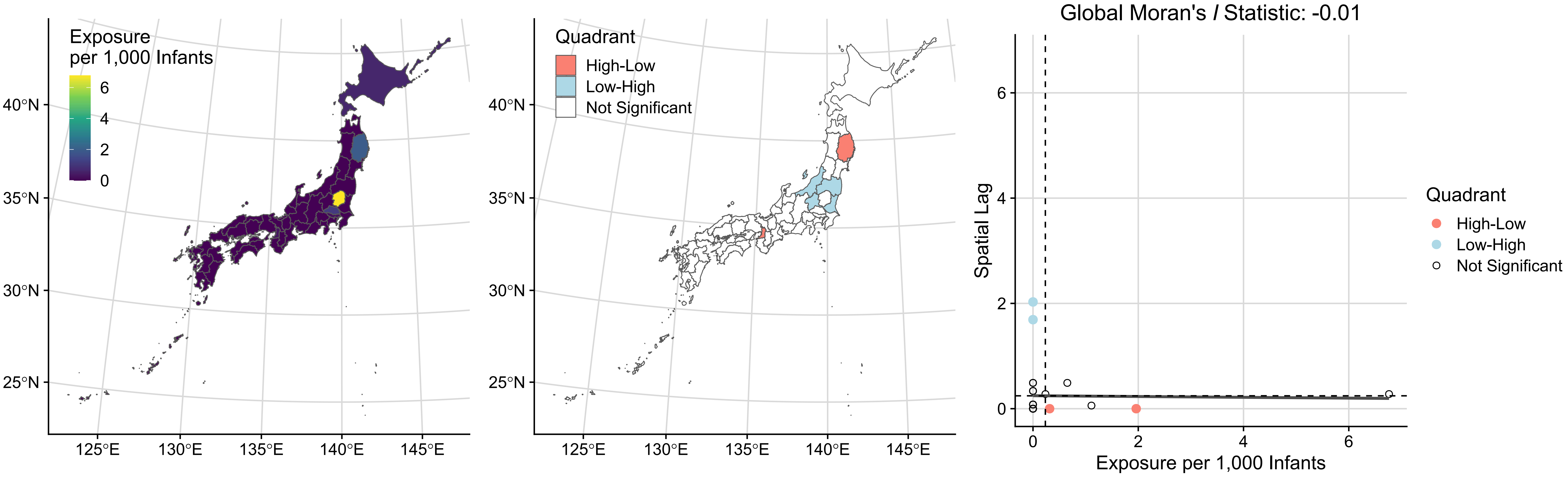

J01BA01. Chloramphenicol

Early Neonatal Exposure among Very Preterm and Very Low Birth Weight Infants (Days 0–6)

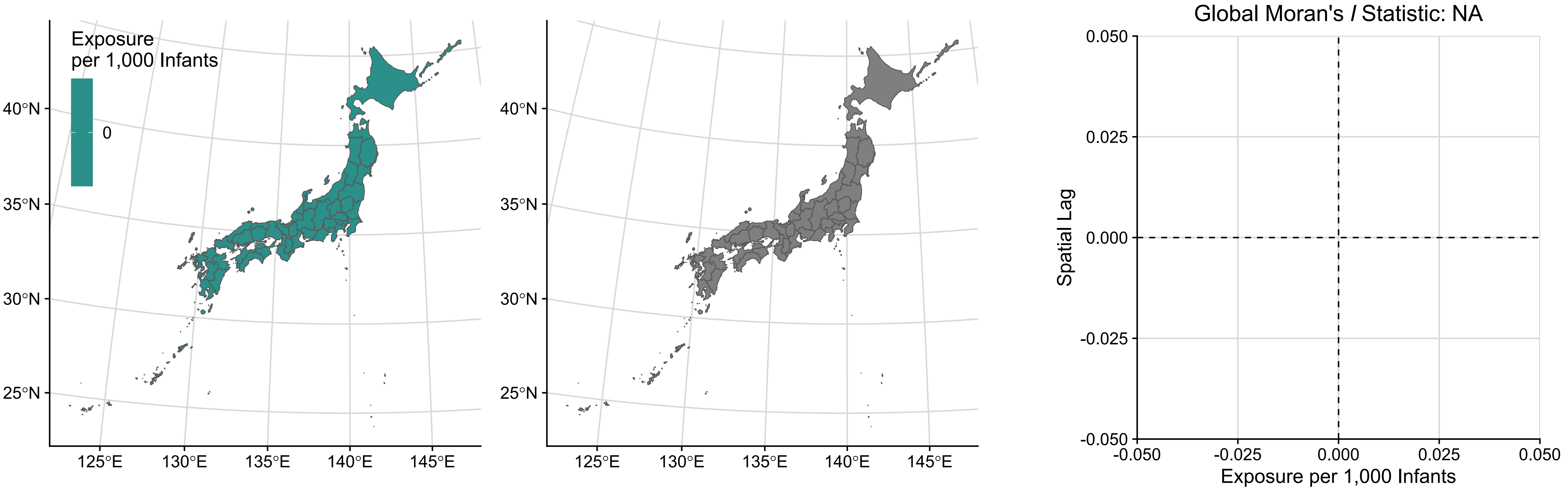

Neonatal Exposure among Very Preterm and Very Low Birth Weight Infants (Days 0–27)

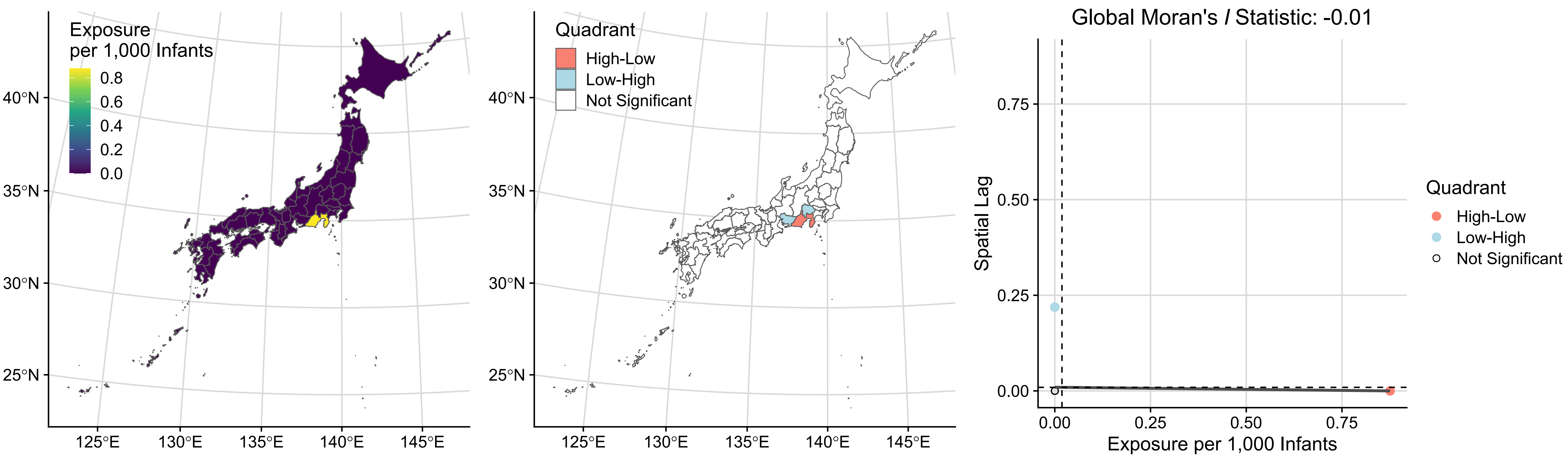

J01CA01. Ampicillin

Early Neonatal Exposure among Very Preterm and Very Low Birth Weight Infants (Days 0–6)

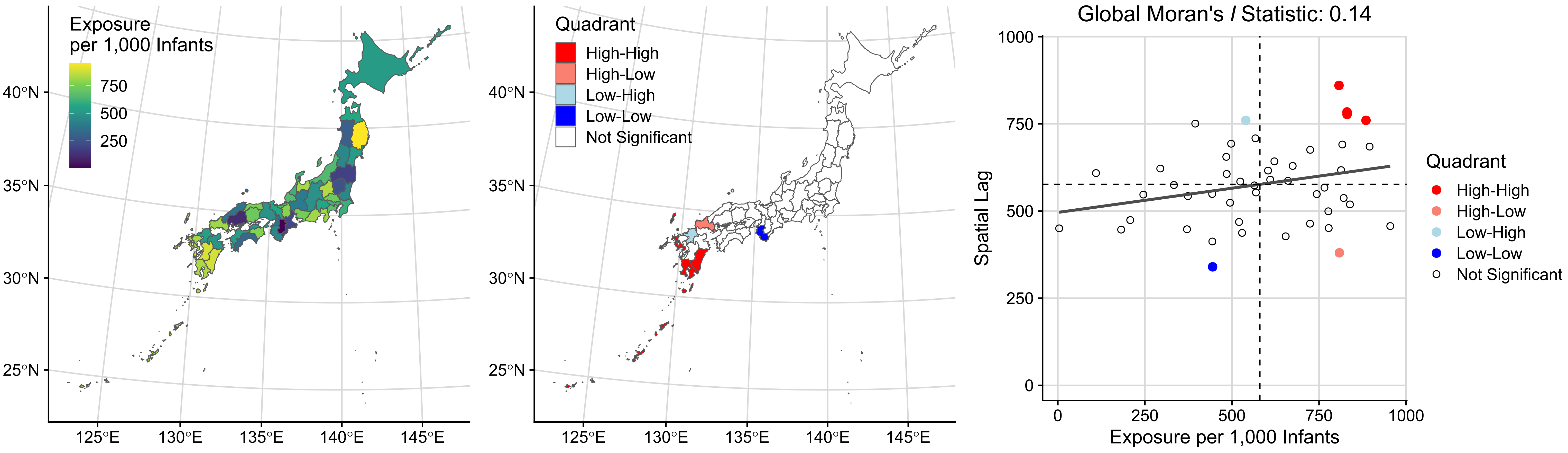

Neonatal Exposure among Very Preterm and Very Low Birth Weight Infants (Days 0–27)

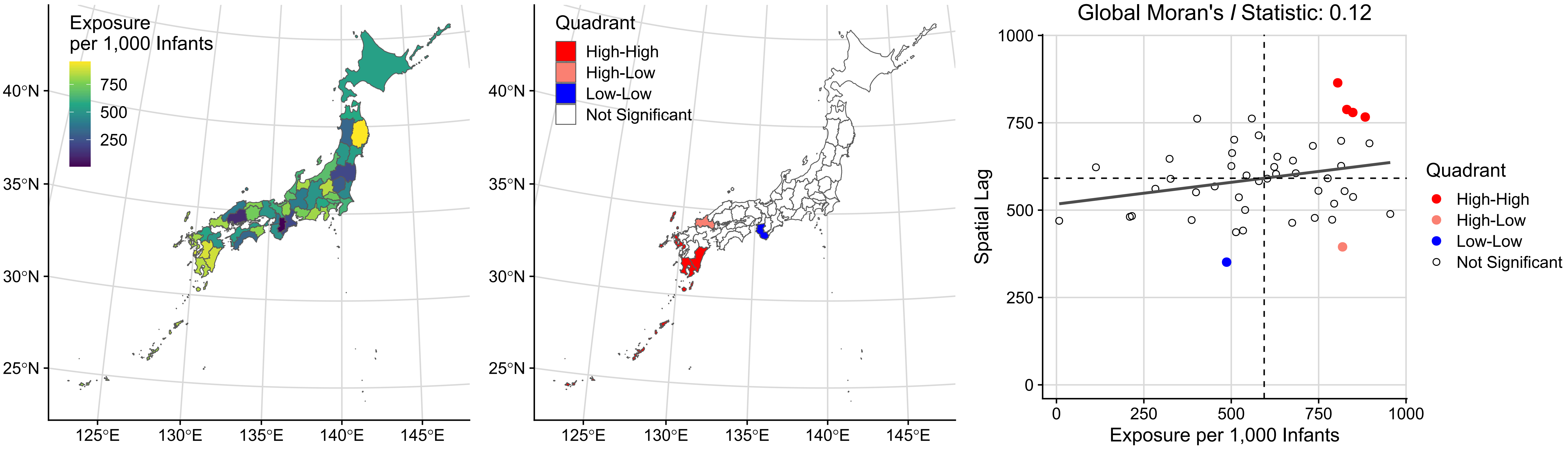

J01CA12. Piperacillin

Early Neonatal Exposure among Very Preterm and Very Low Birth Weight Infants (Days 0–6)

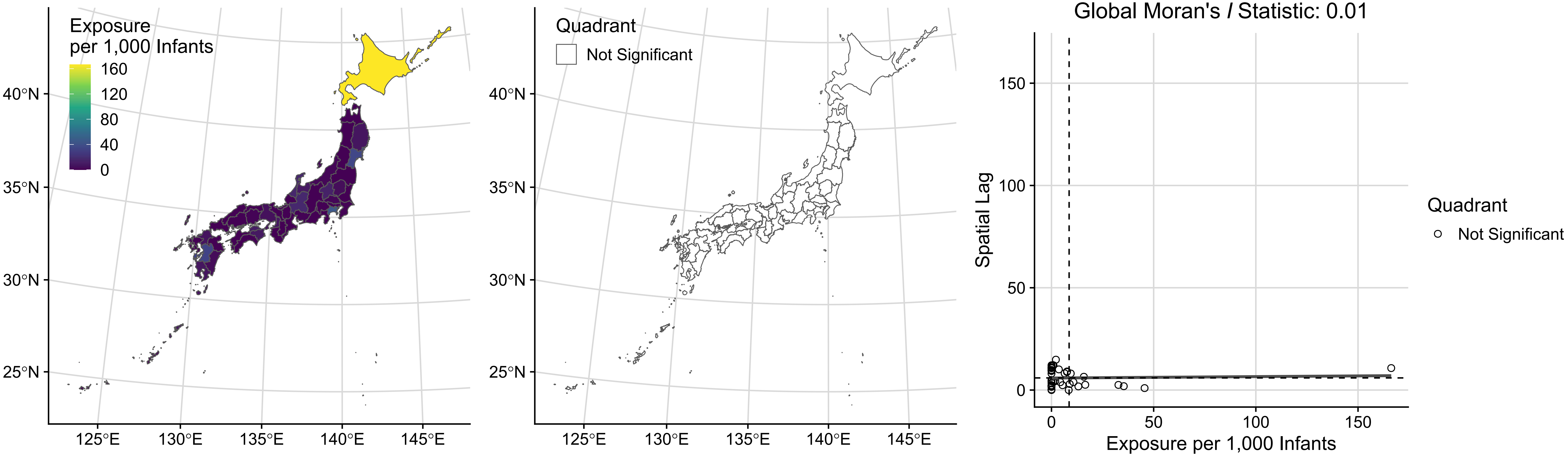

Neonatal Exposure among Very Preterm and Very Low Birth Weight Infants (Days 0–27)

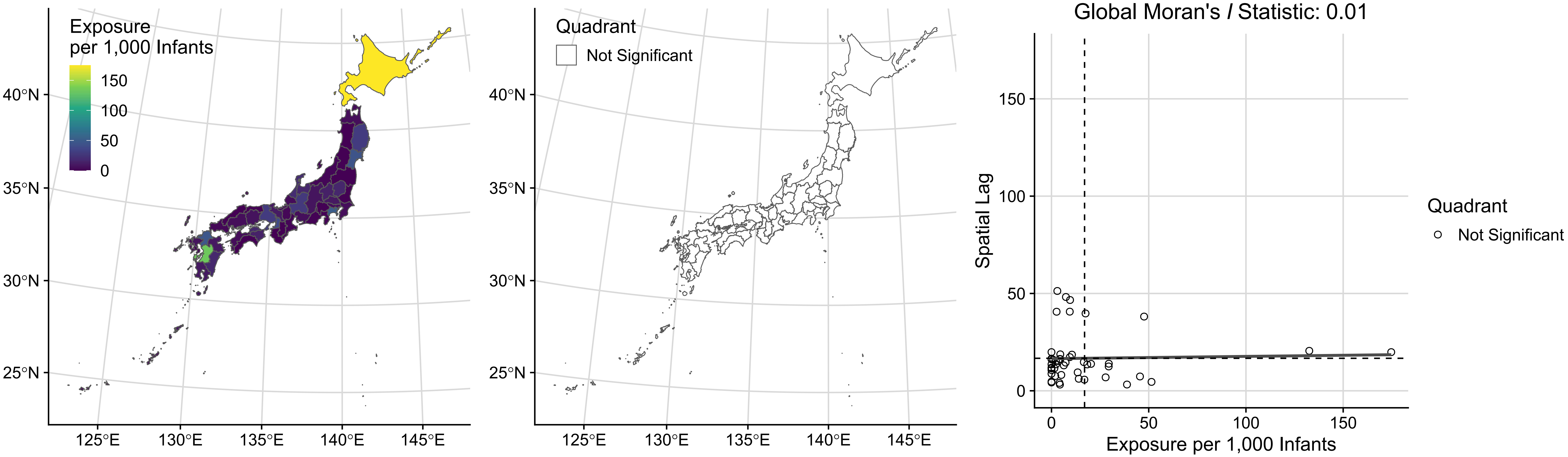

J01CA19. Aspoxicillin

Early Neonatal Exposure among Very Preterm and Very Low Birth Weight Infants (Days 0–6)

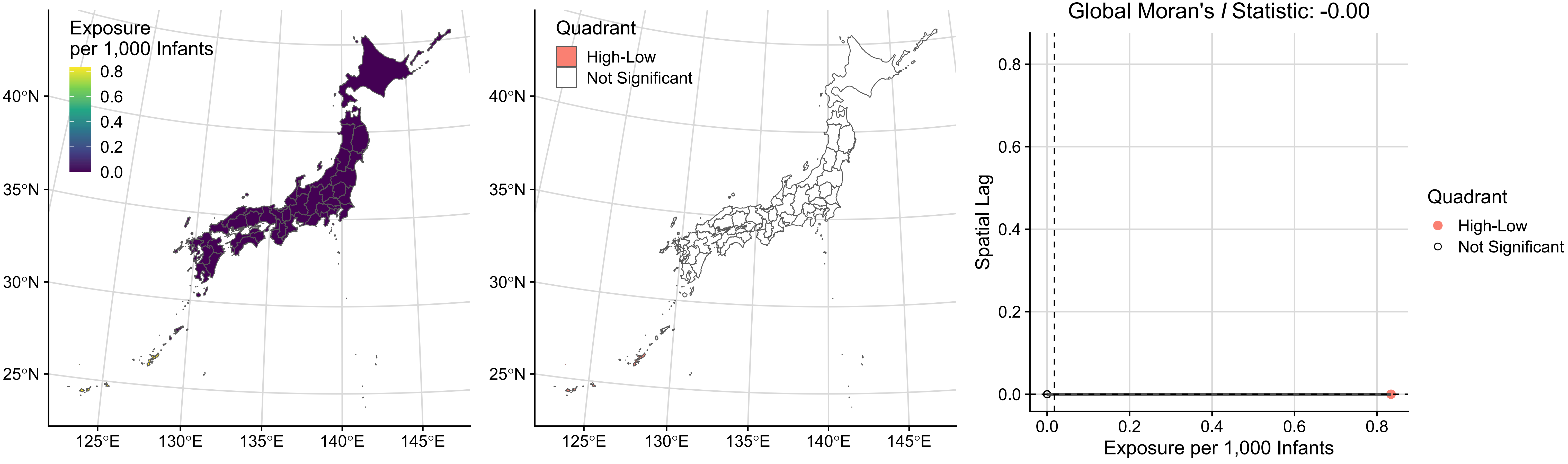

Neonatal Exposure among Very Preterm and Very Low Birth Weight Infants (Days 0–27)

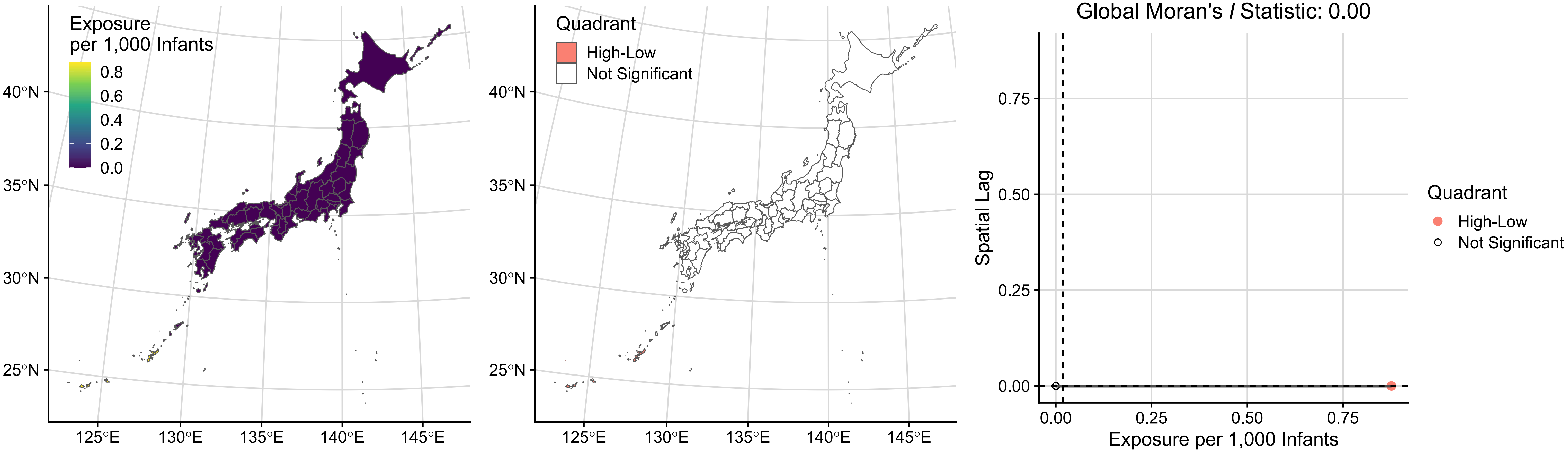

J01CA51. Ampicillin, Combinations

Early Neonatal Exposure among Very Preterm and Very Low Birth Weight Infants (Days 0–6)

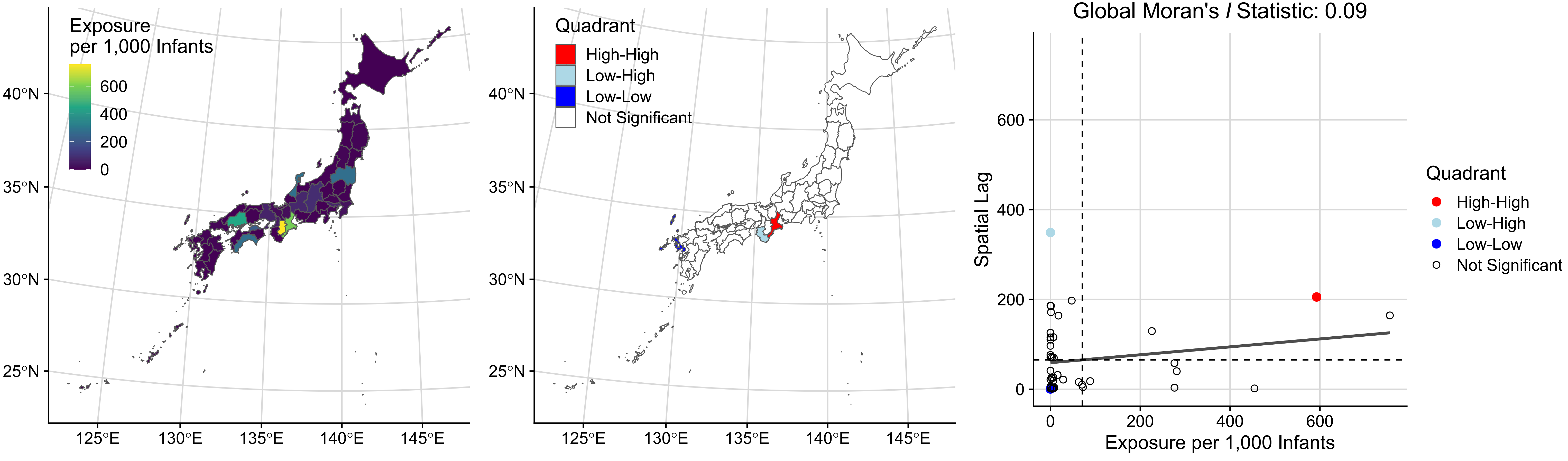

Neonatal Exposure among Very Preterm and Very Low Birth Weight Infants (Days 0–27)

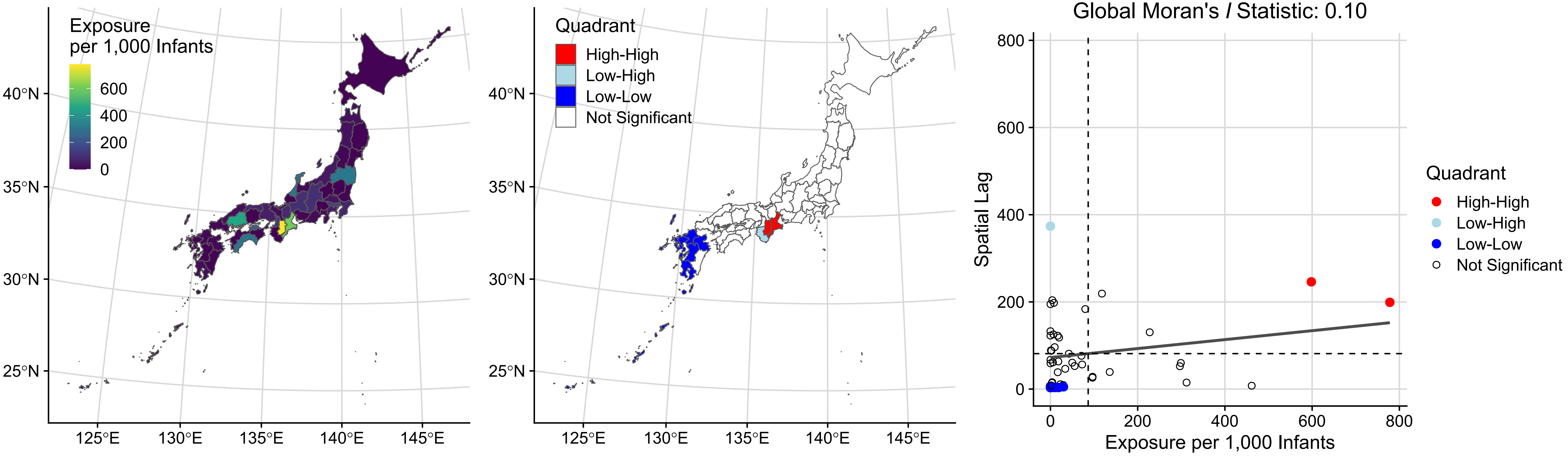

J01CE01. Benzylpenicillin

Early Neonatal Exposure among Very Preterm and Very Low Birth Weight Infants (Days 0–6)

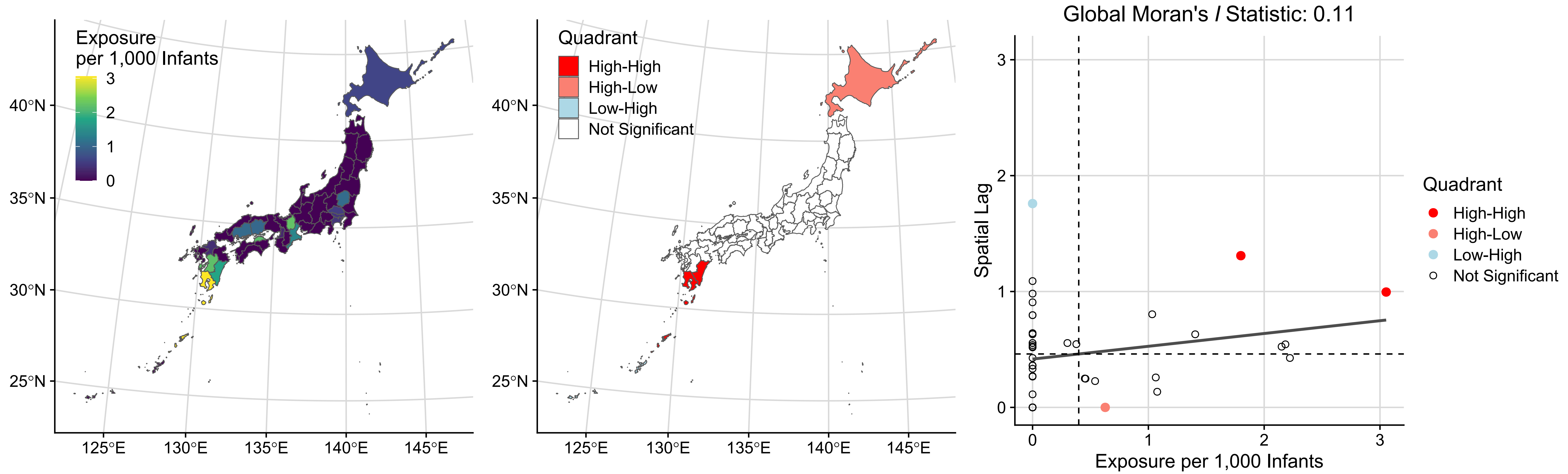

Neonatal Exposure among Very Preterm and Very Low Birth Weight Infants (Days 0–27)

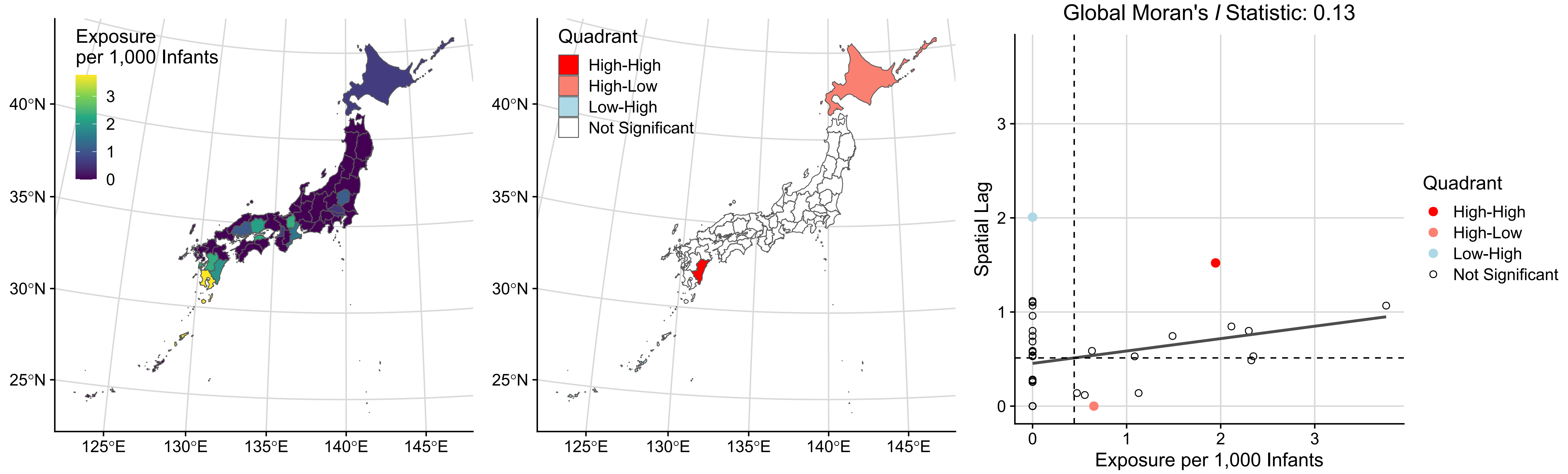

J01CR05. Piperacillin and Beta-Lactamase Inhibitor

Early Neonatal Exposure among Very Preterm and Very Low Birth Weight Infants (Days 0–6)

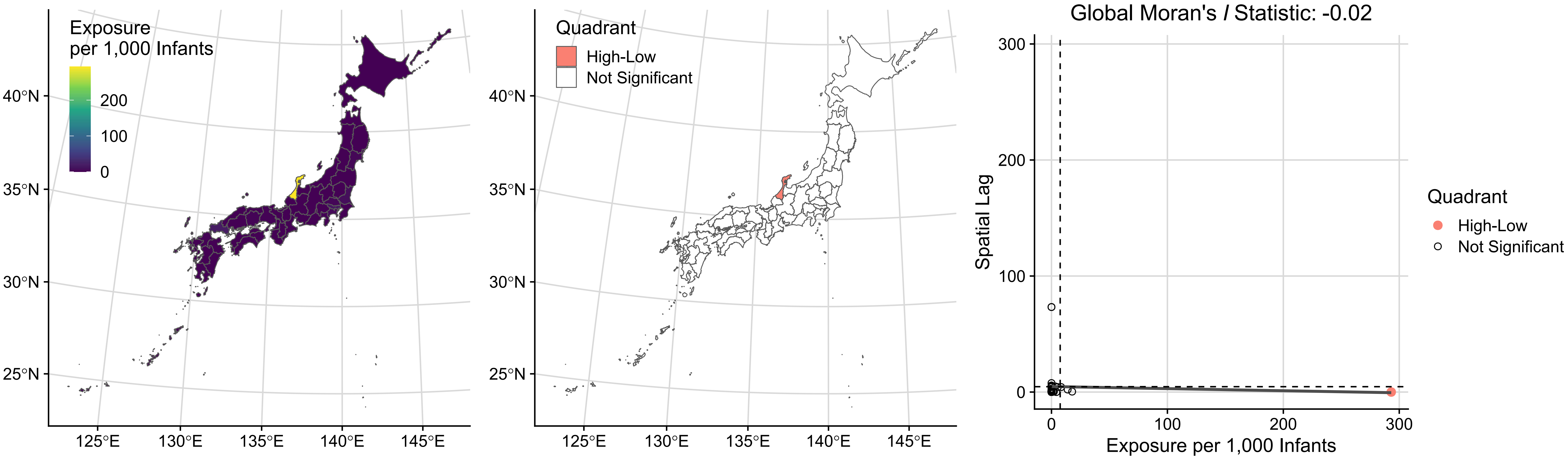

Neonatal Exposure among Very Preterm and Very Low Birth Weight Infants (Days 0–27)

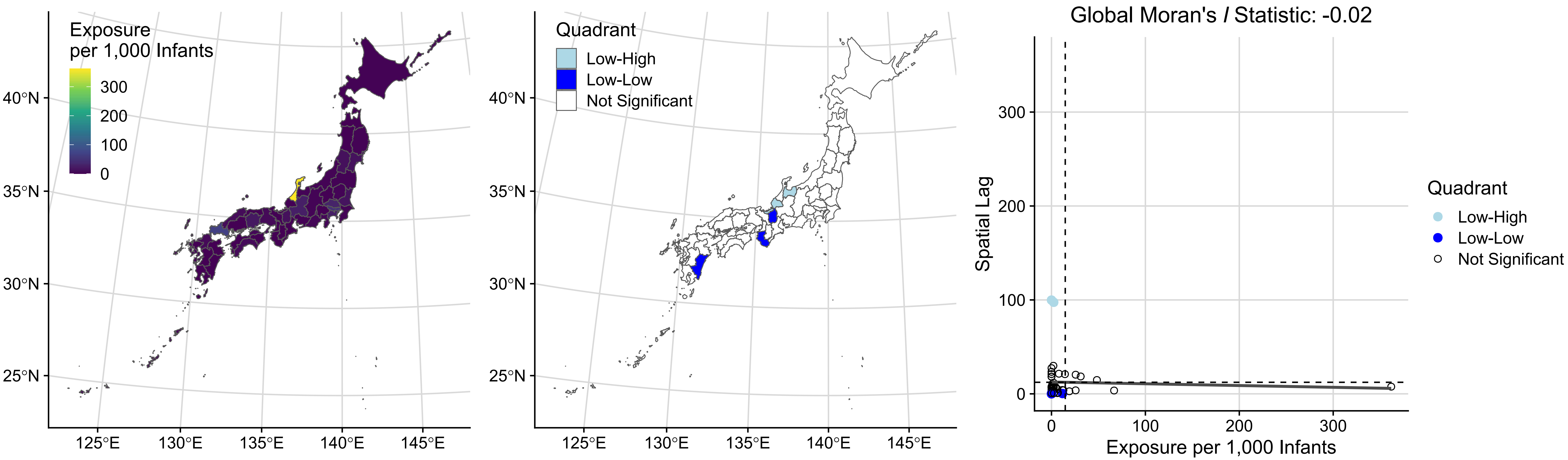

J01DB04. Cefazolin

Early Neonatal Exposure among Very Preterm and Very Low Birth Weight Infants (Days 0–6)

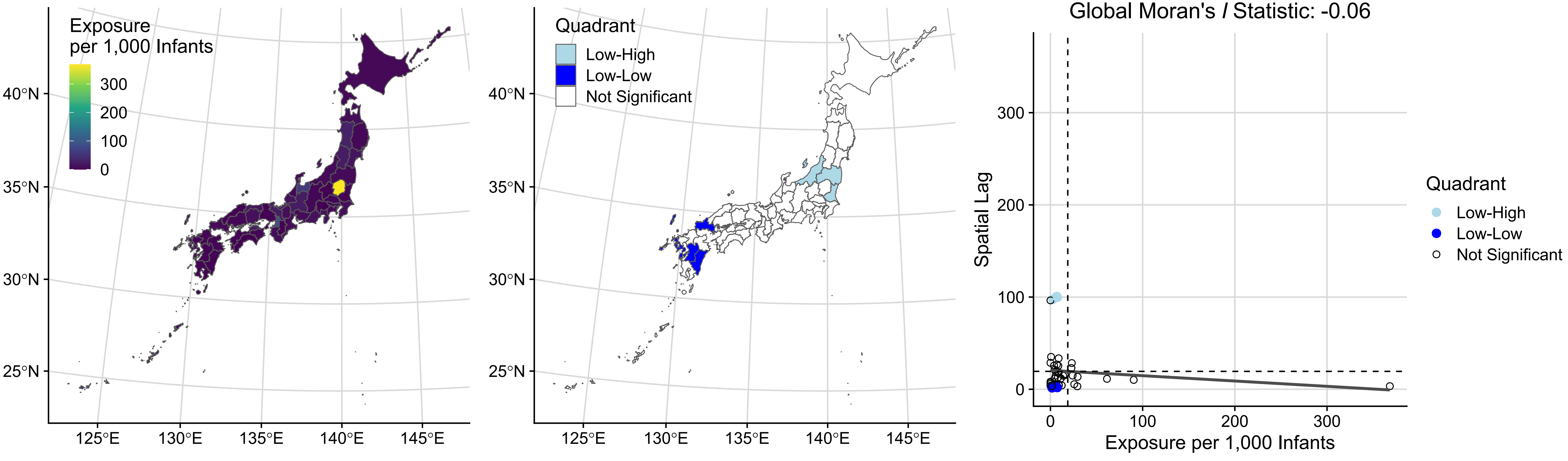

Neonatal Exposure among Very Preterm and Very Low Birth Weight Infants (Days 0–27)

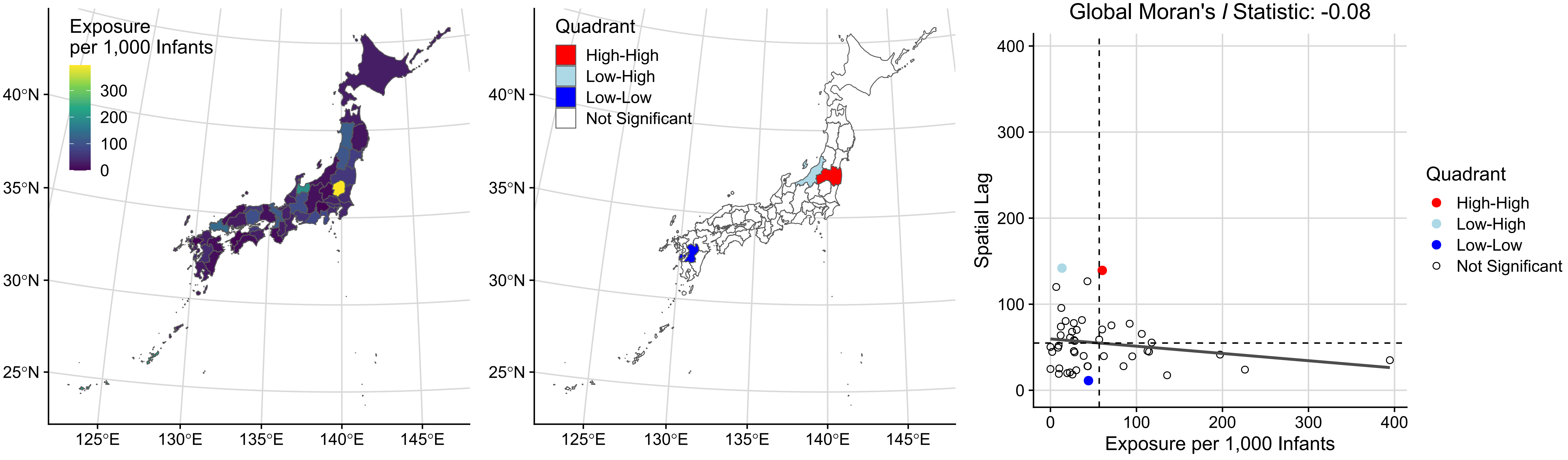

J01DC07. Cefotiam

Early Neonatal Exposure among Very Preterm and Very Low Birth Weight Infants (Days 0–6)

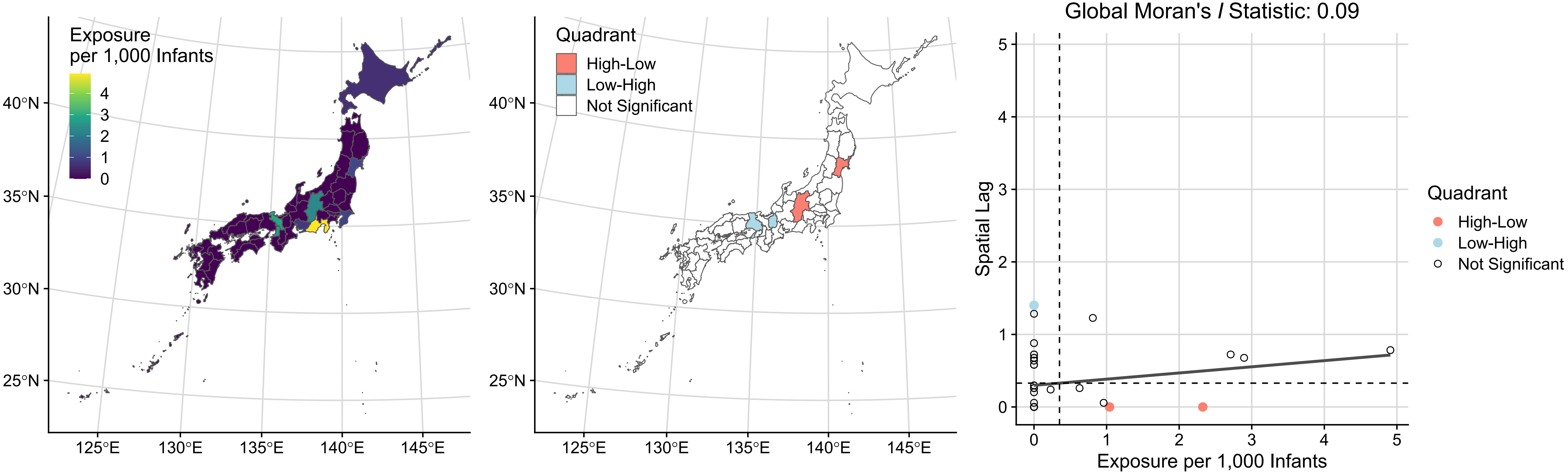

Neonatal Exposure among Very Preterm and Very Low Birth Weight Infants (Days 0–27)

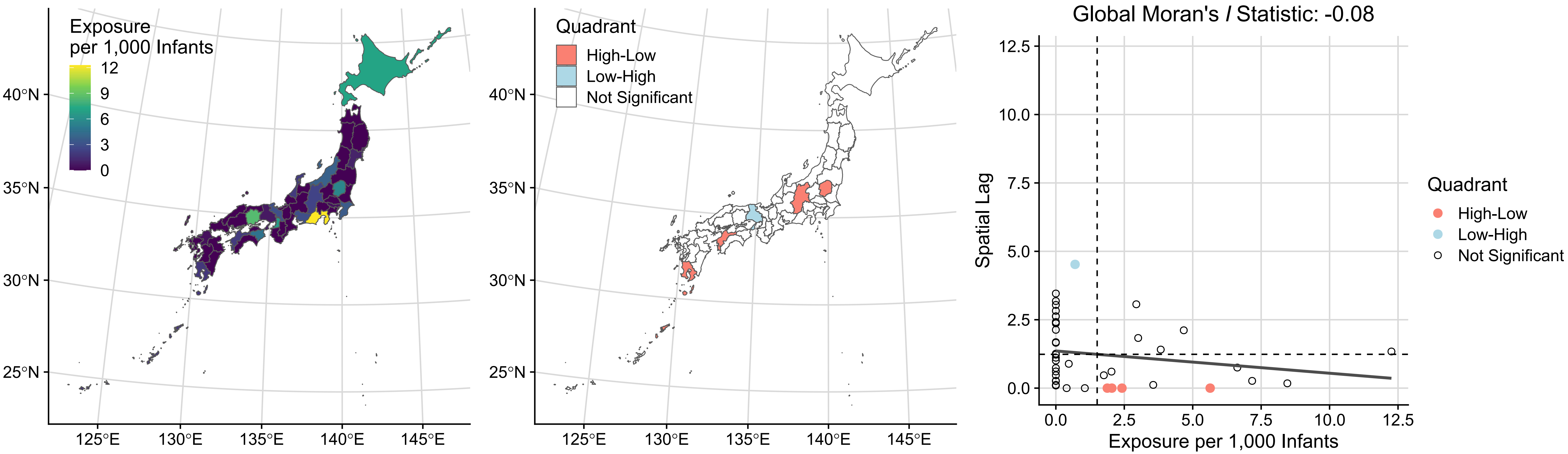

J01DC09. Cefmetazole

Early Neonatal Exposure among Very Preterm and Very Low Birth Weight Infants (Days 0–6)

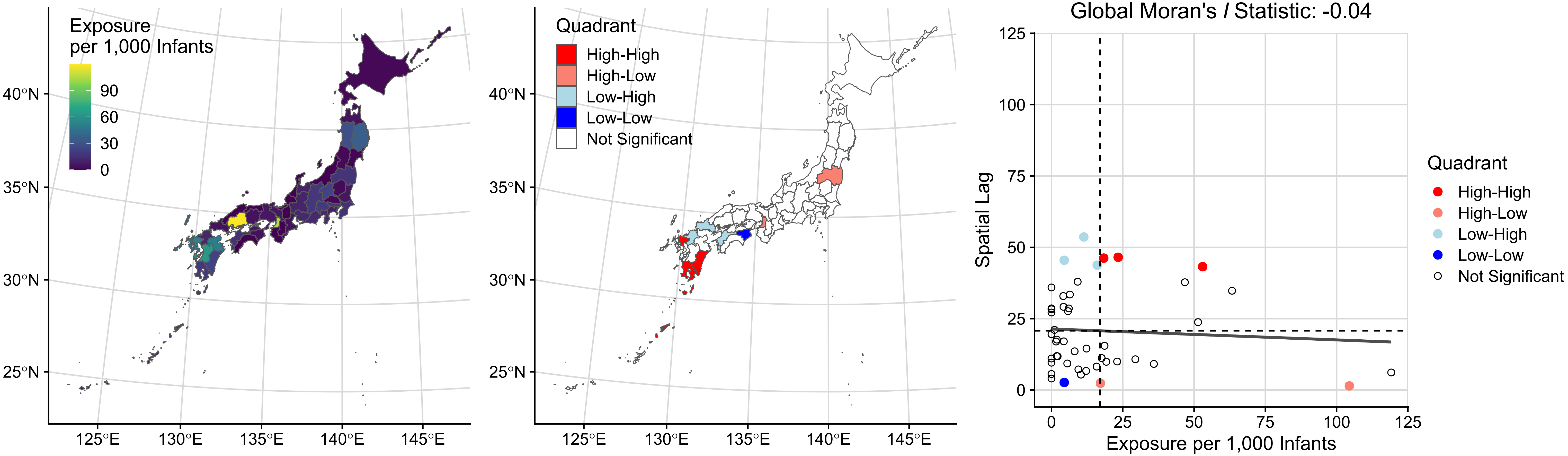

Neonatal Exposure among Very Preterm and Very Low Birth Weight Infants (Days 0–27)

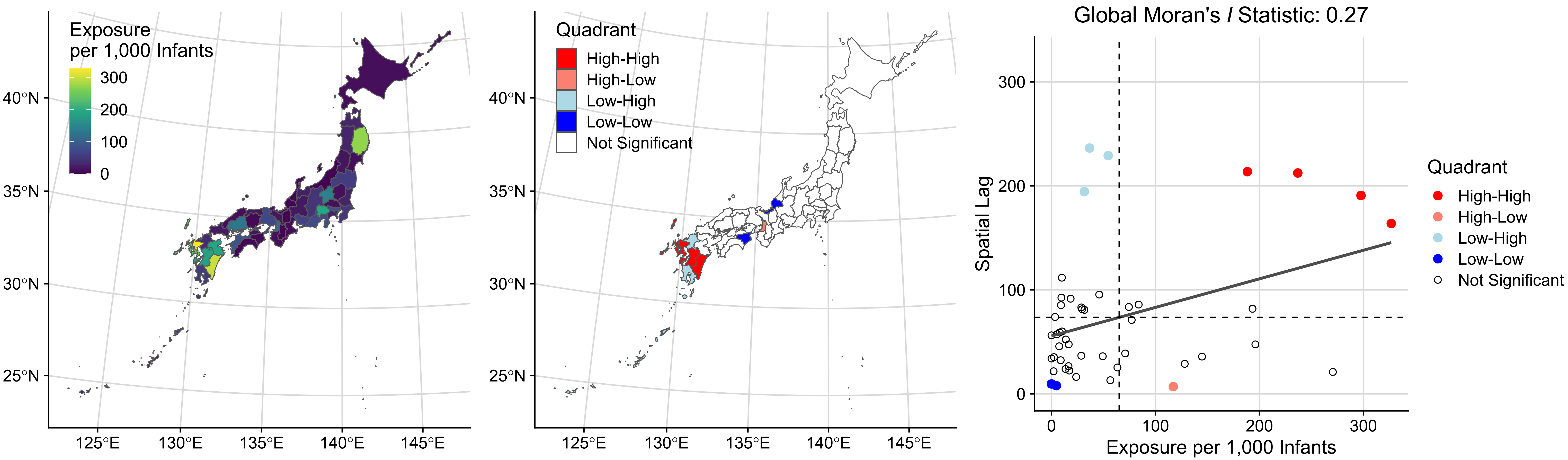

J01DC14. Flomoxef

Early Neonatal Exposure among Very Preterm and Very Low Birth Weight Infants (Days 0–6)

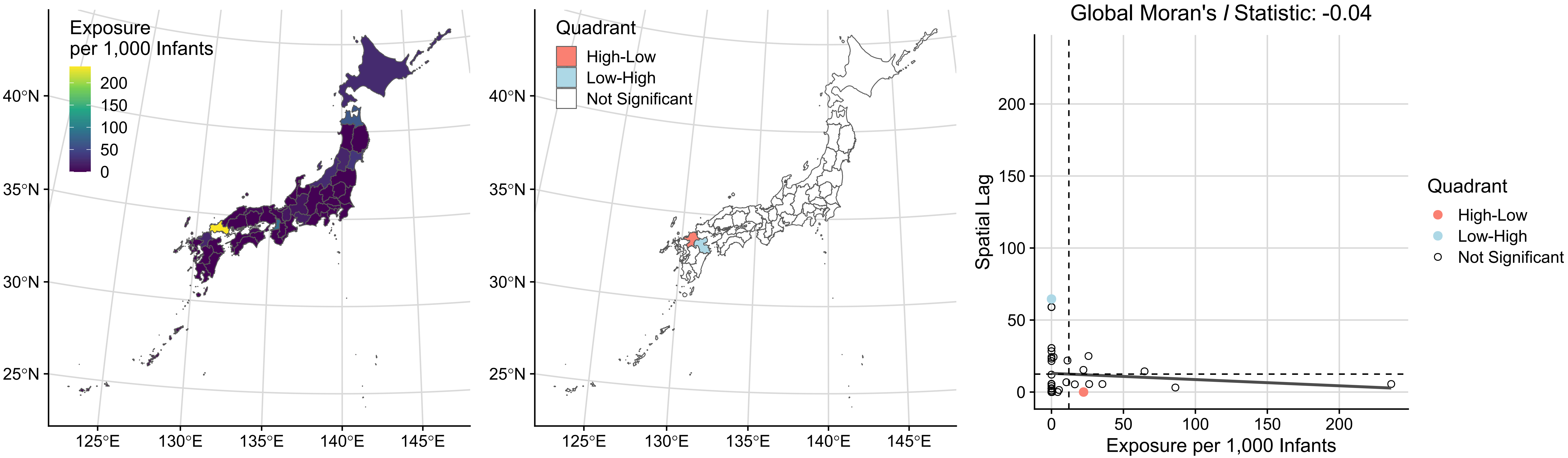

Neonatal Exposure among Very Preterm and Very Low Birth Weight Infants (Days 0–27)

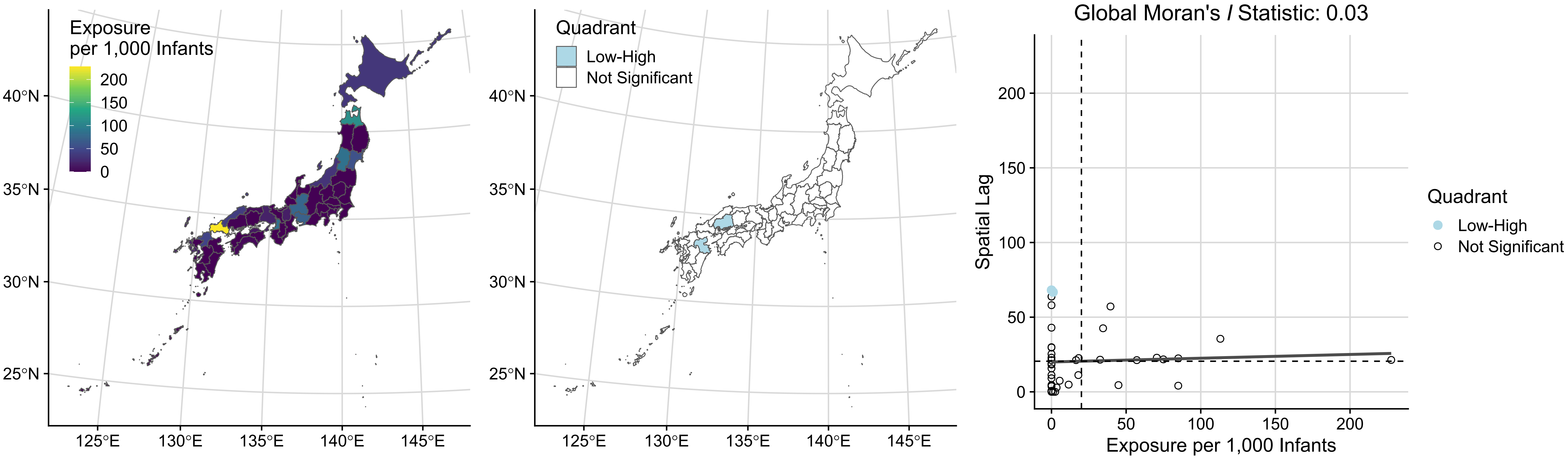

J01DD01. Cefotaxime

Early Neonatal Exposure among Very Preterm and Very Low Birth Weight Infants (Days 0–6)

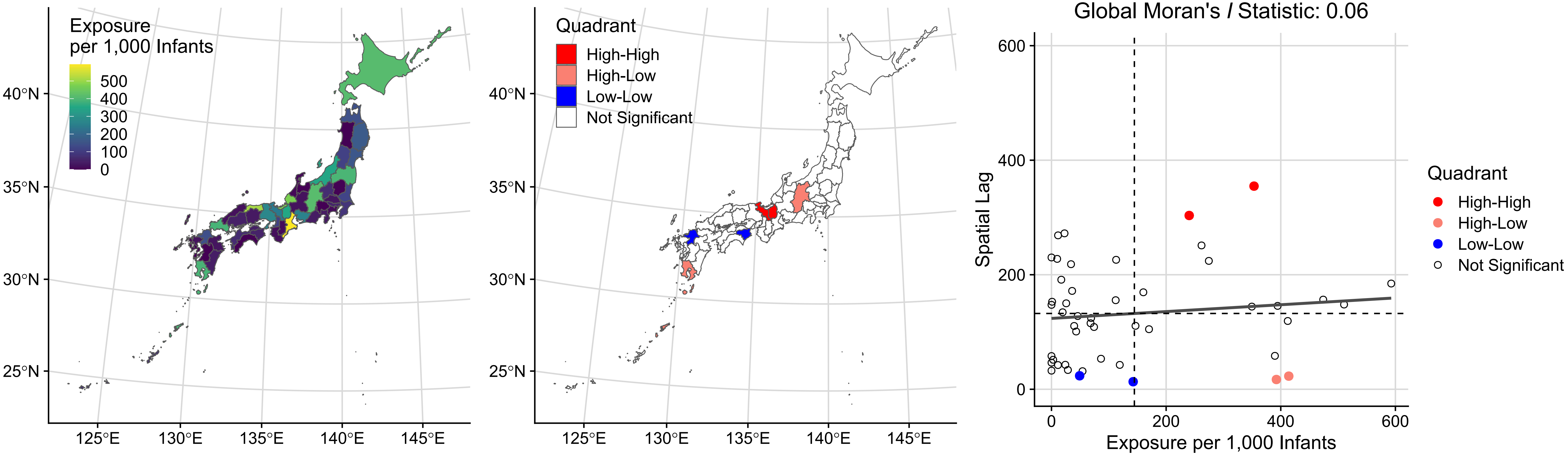

Neonatal Exposure among Very Preterm and Very Low Birth Weight Infants (Days 0–27)

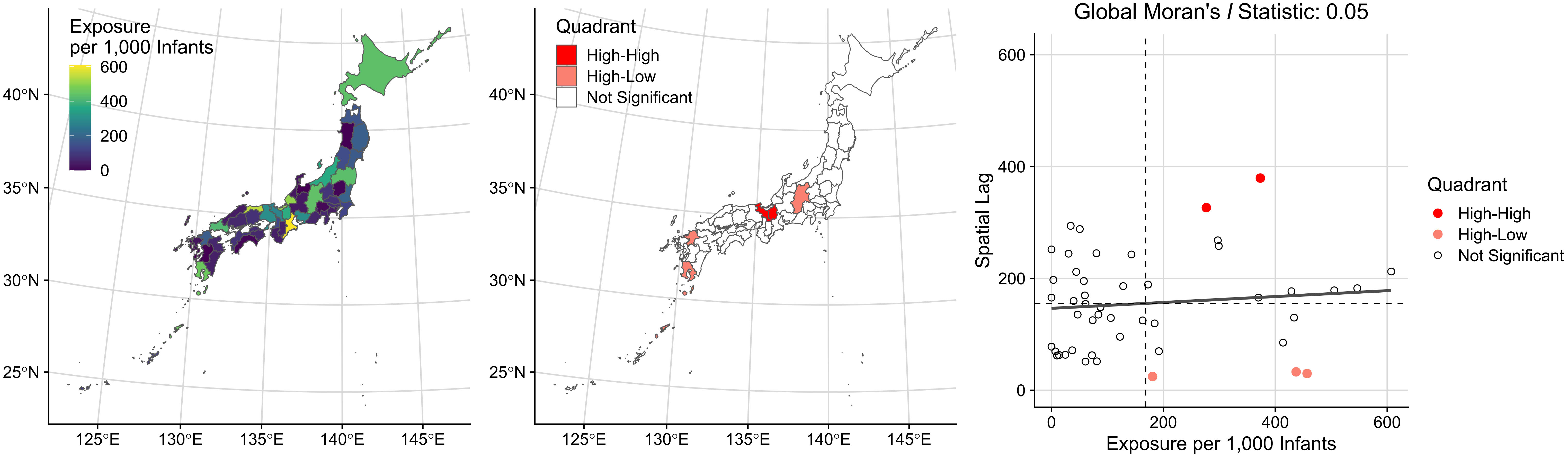

J01DD02. Ceftazidime

Early Neonatal Exposure among Very Preterm and Very Low Birth Weight Infants (Days 0–6)

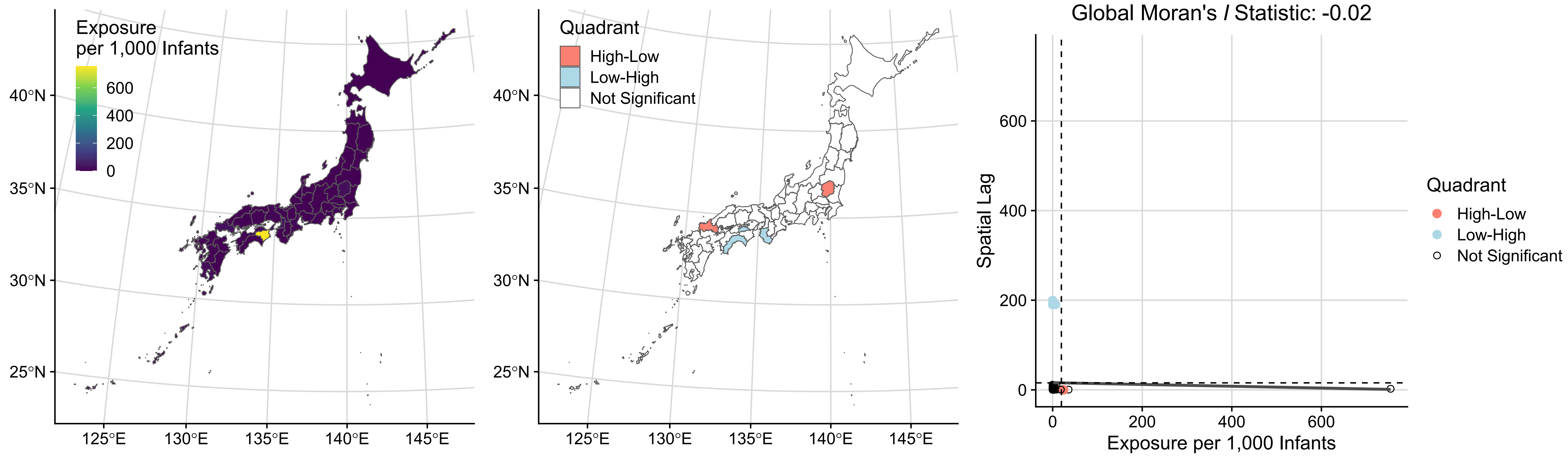

Neonatal Exposure among Very Preterm and Very Low Birth Weight Infants (Days 0–27)

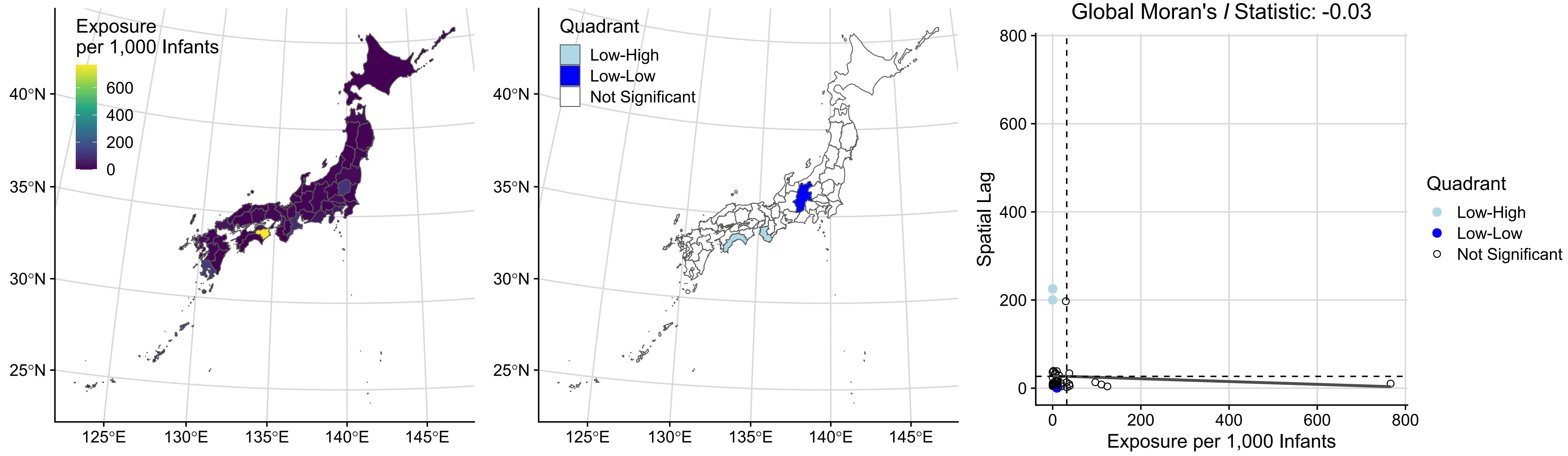

J01DD04. Ceftriaxone

Early Neonatal Exposure among Very Preterm and Very Low Birth Weight Infants (Days 0–6)

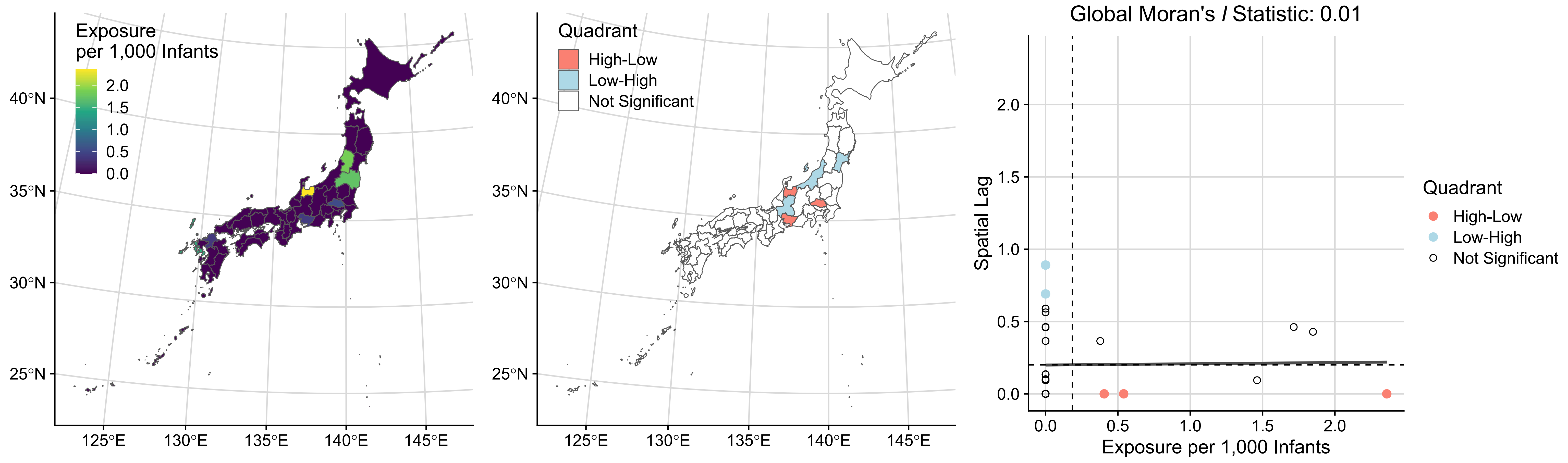

Neonatal Exposure among Very Preterm and Very Low Birth Weight Infants (Days 0–27)

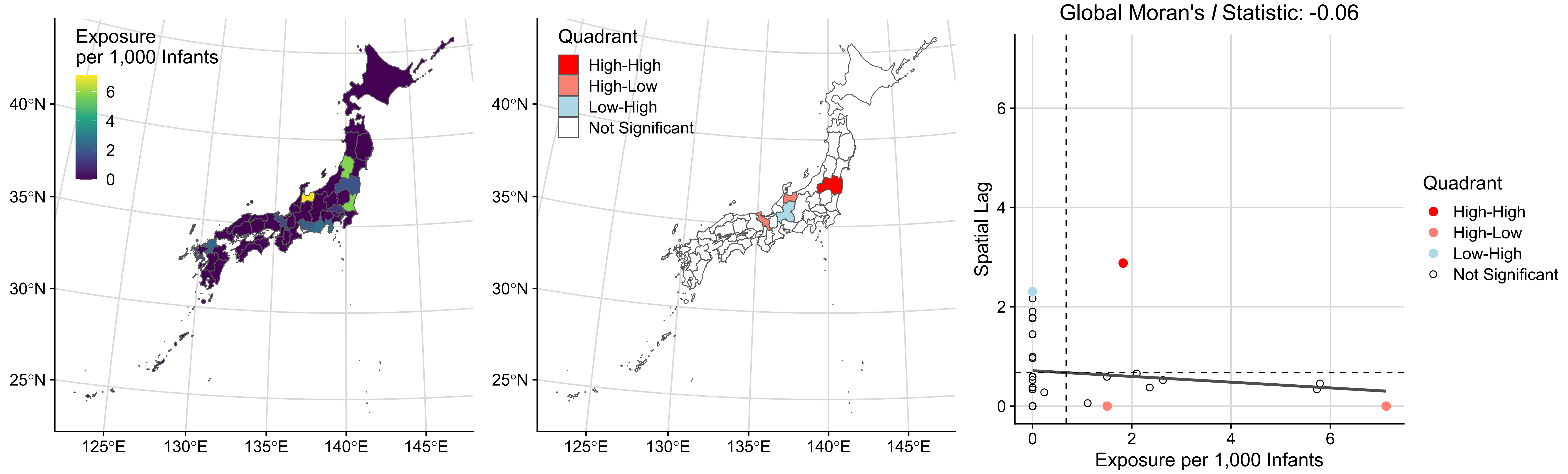

J01DD62. Cefoperazone and Beta-Lactamase Inhibitor

Early Neonatal Exposure among Very Preterm and Very Low Birth Weight Infants (Days 0–6)

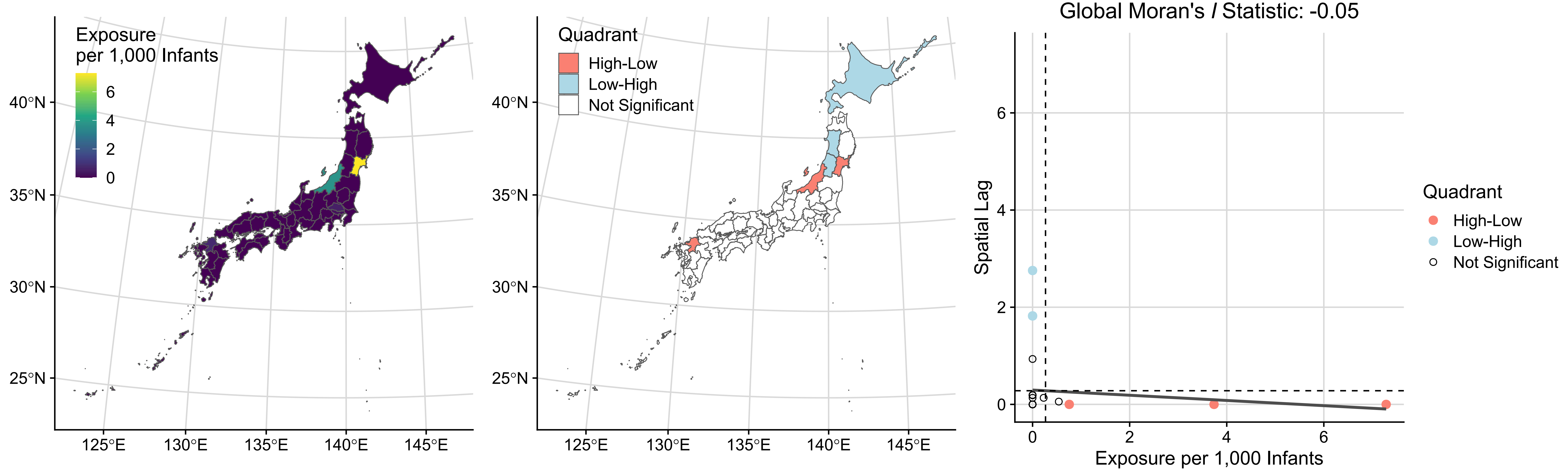

Neonatal Exposure among Very Preterm and Very Low Birth Weight Infants (Days 0–27)

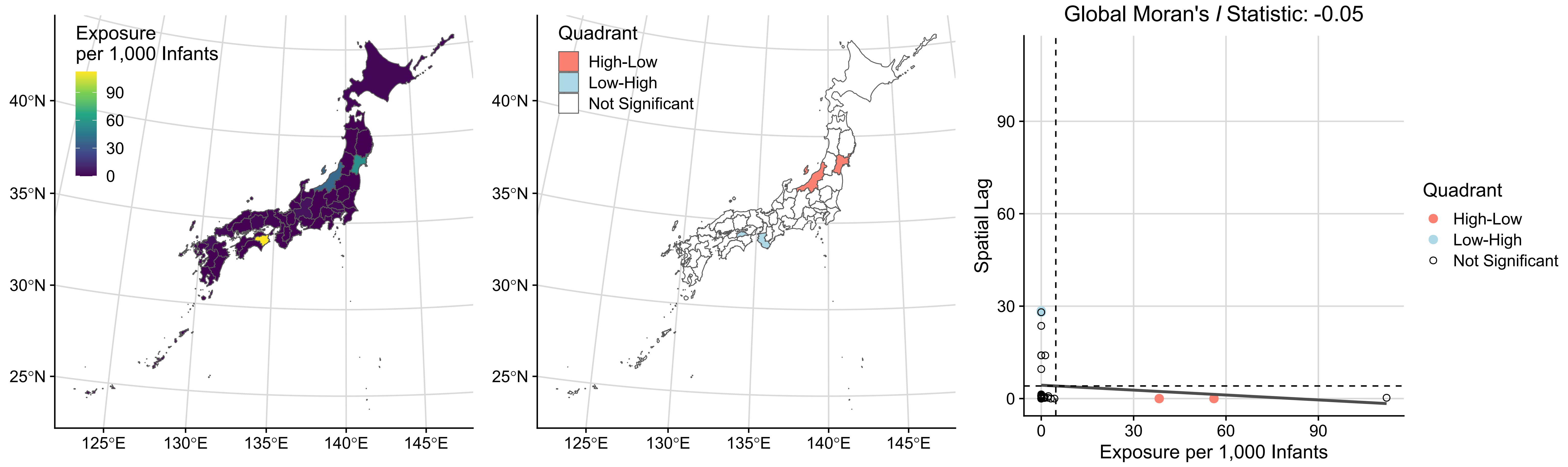

J01DE01. Cefepime

Early Neonatal Exposure among Very Preterm and Very Low Birth Weight Infants (Days 0–6)

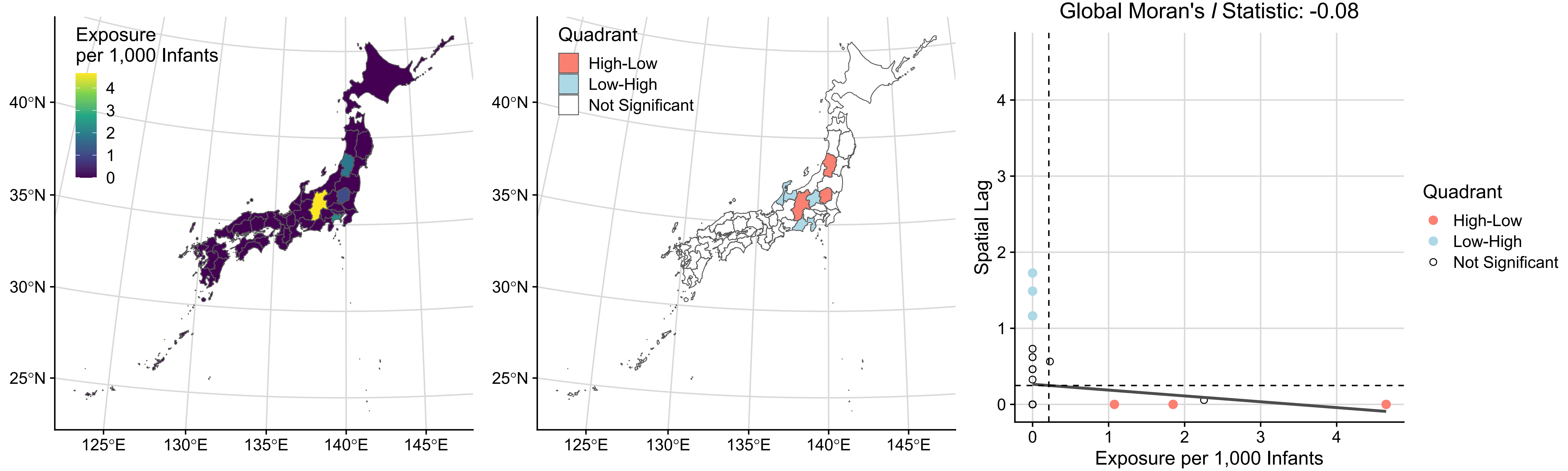

Neonatal Exposure among Very Preterm and Very Low Birth Weight Infants (Days 0–27)

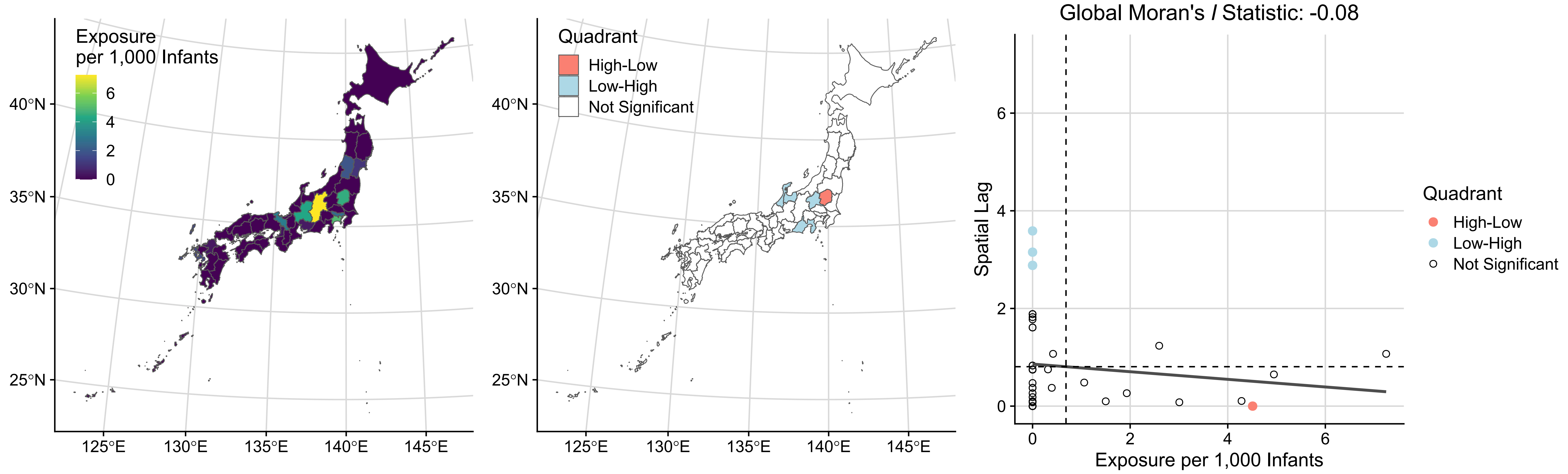

J01DE02. Cefpirome

Early Neonatal Exposure among Very Preterm and Very Low Birth Weight Infants (Days 0–6)

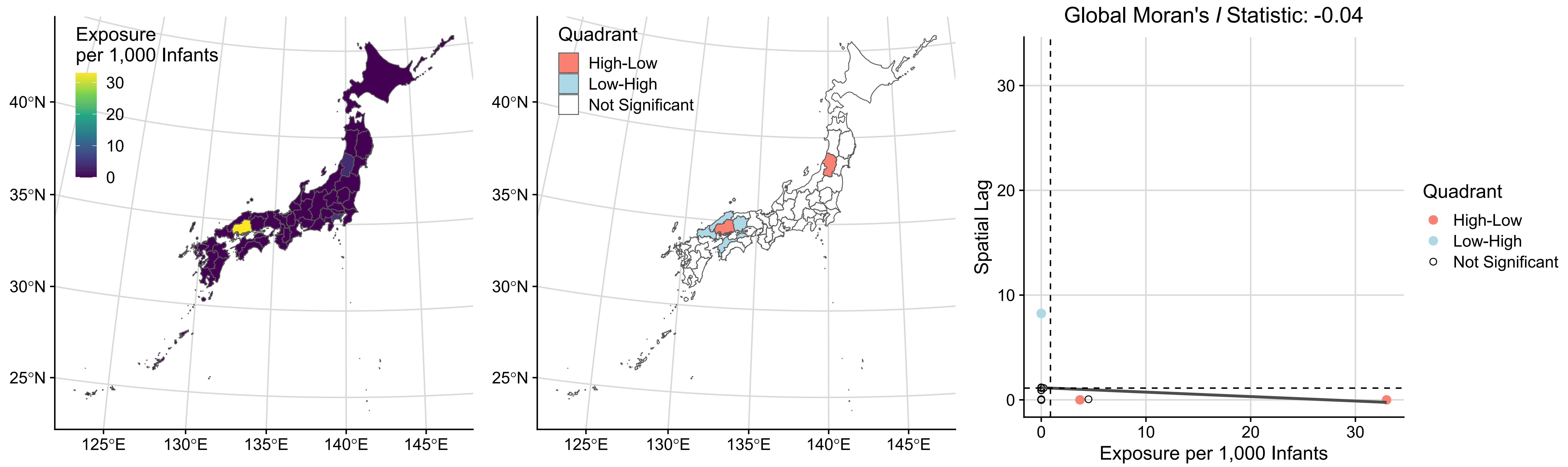

Neonatal Exposure among Very Preterm and Very Low Birth Weight Infants (Days 0–27)

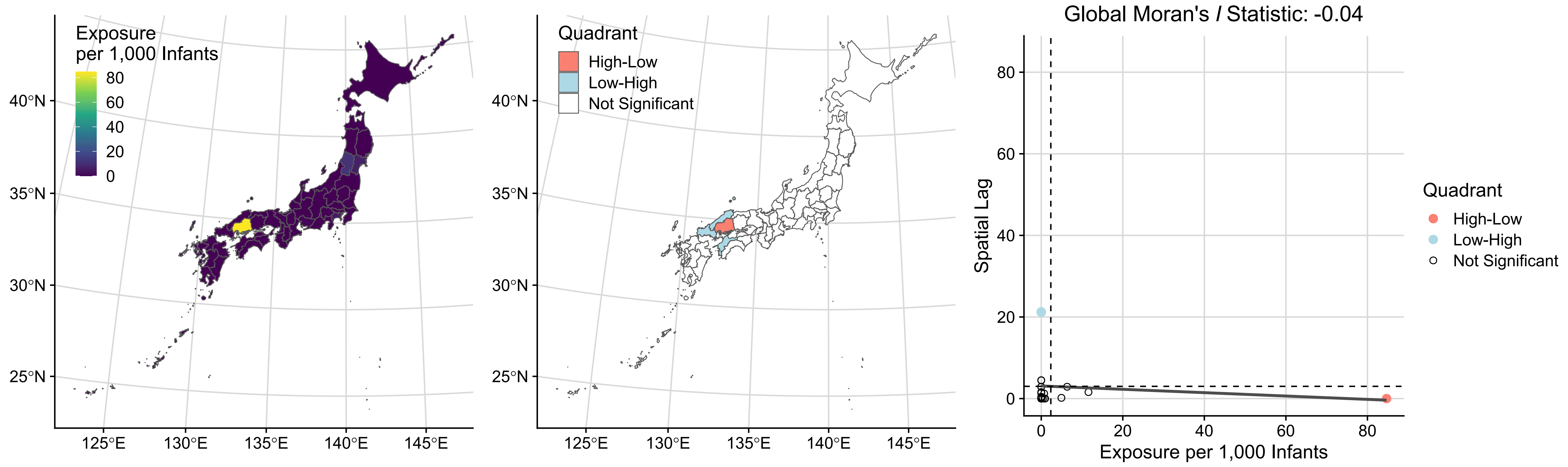

J01DE03. Cefozopran

Early Neonatal Exposure among Very Preterm and Very Low Birth Weight Infants (Days 0–6)

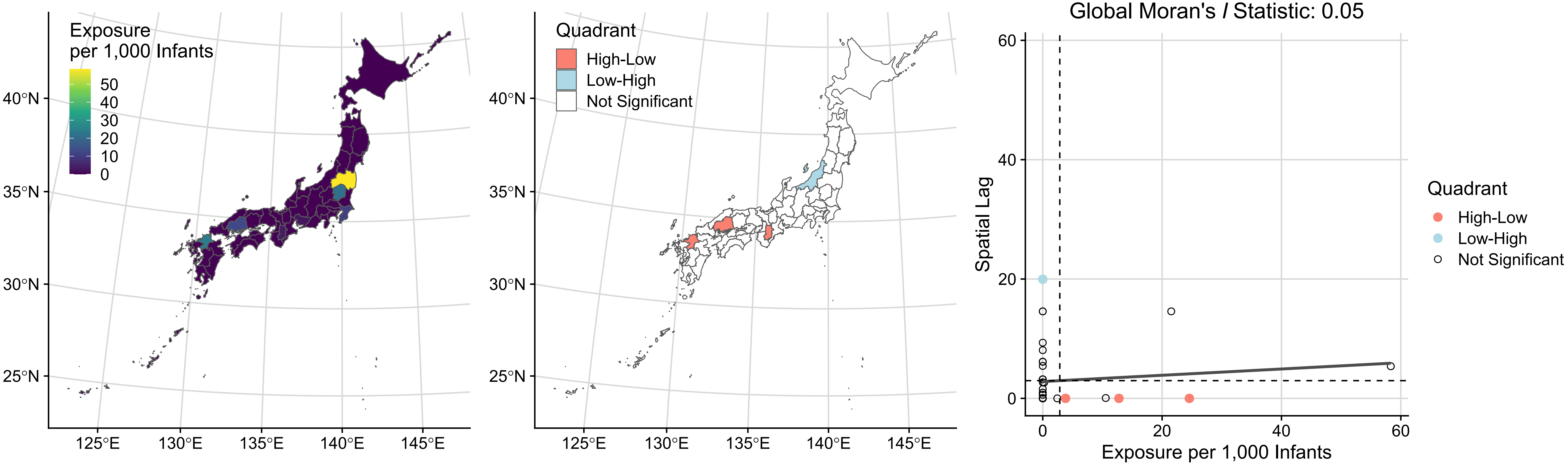

Neonatal Exposure among Very Preterm and Very Low Birth Weight Infants (Days 0–27)

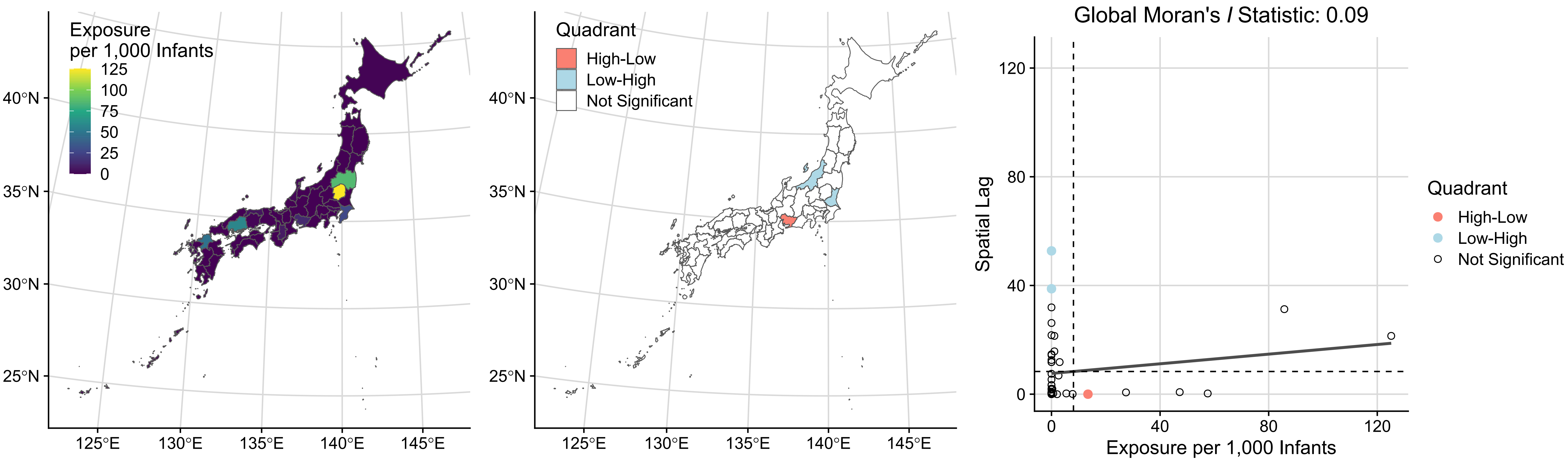

J01DF01. Aztreonam

Early Neonatal Exposure among Very Preterm and Very Low Birth Weight Infants (Days 0–6)

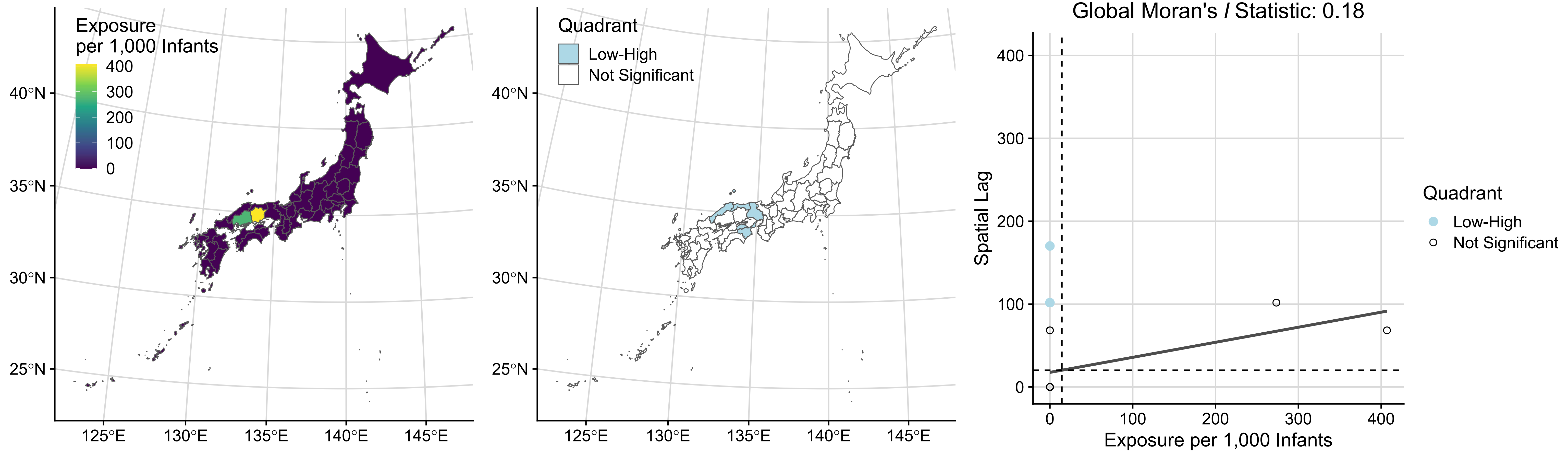

Neonatal Exposure among Very Preterm and Very Low Birth Weight Infants (Days 0–27)

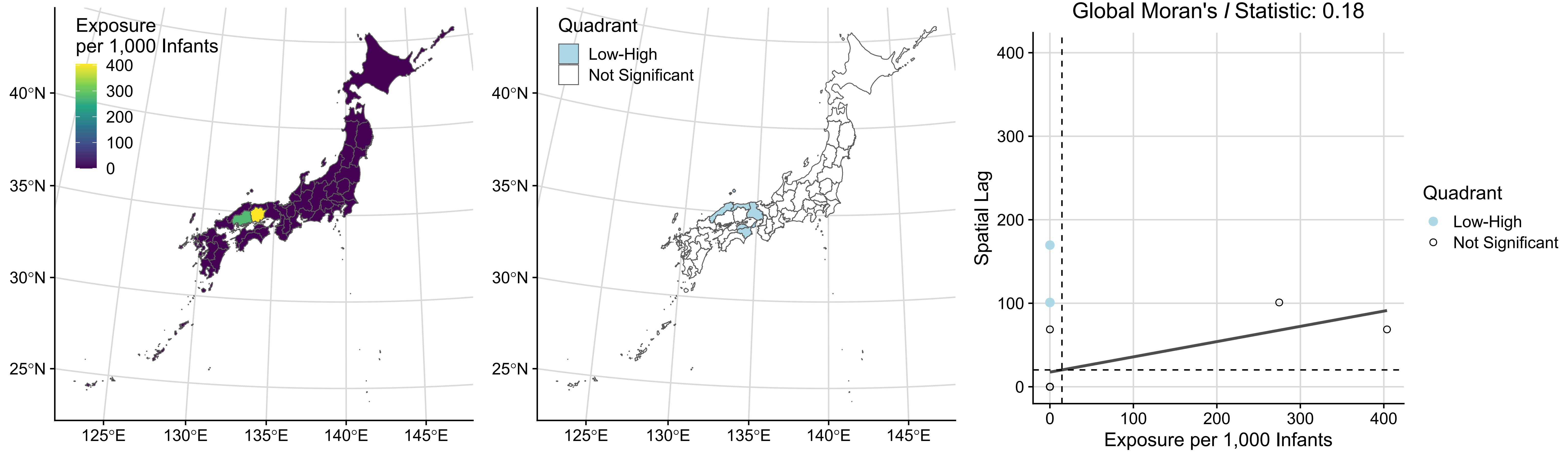

J01DH02. Meropenem

Early Neonatal Exposure among Very Preterm and Very Low Birth Weight Infants (Days 0–6)

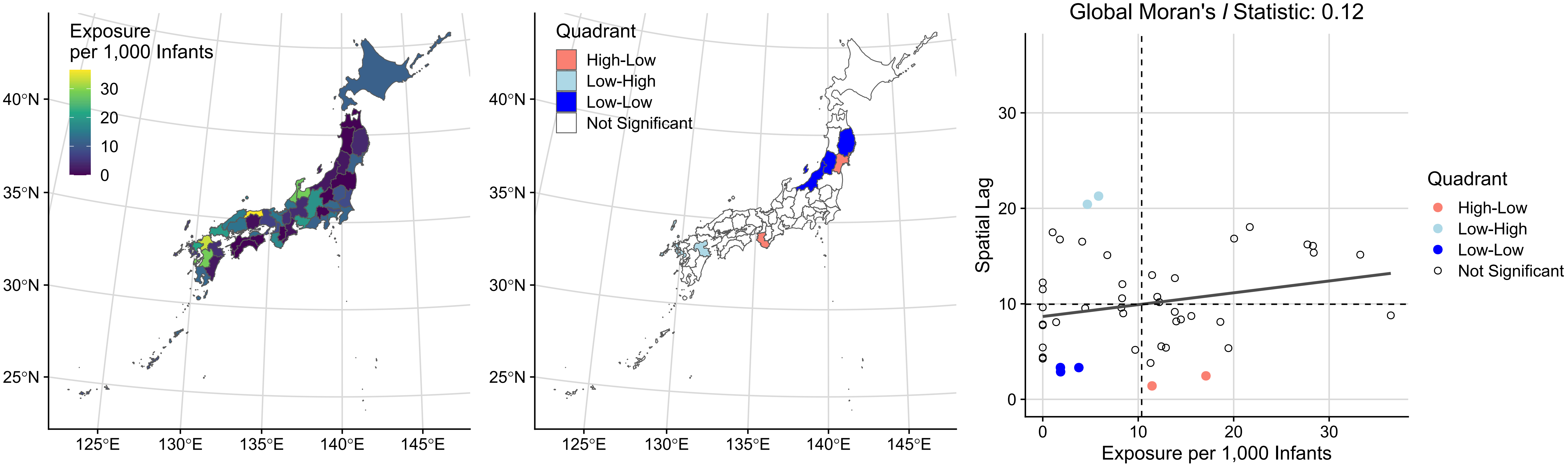

Neonatal Exposure among Very Preterm and Very Low Birth Weight Infants (Days 0–27)

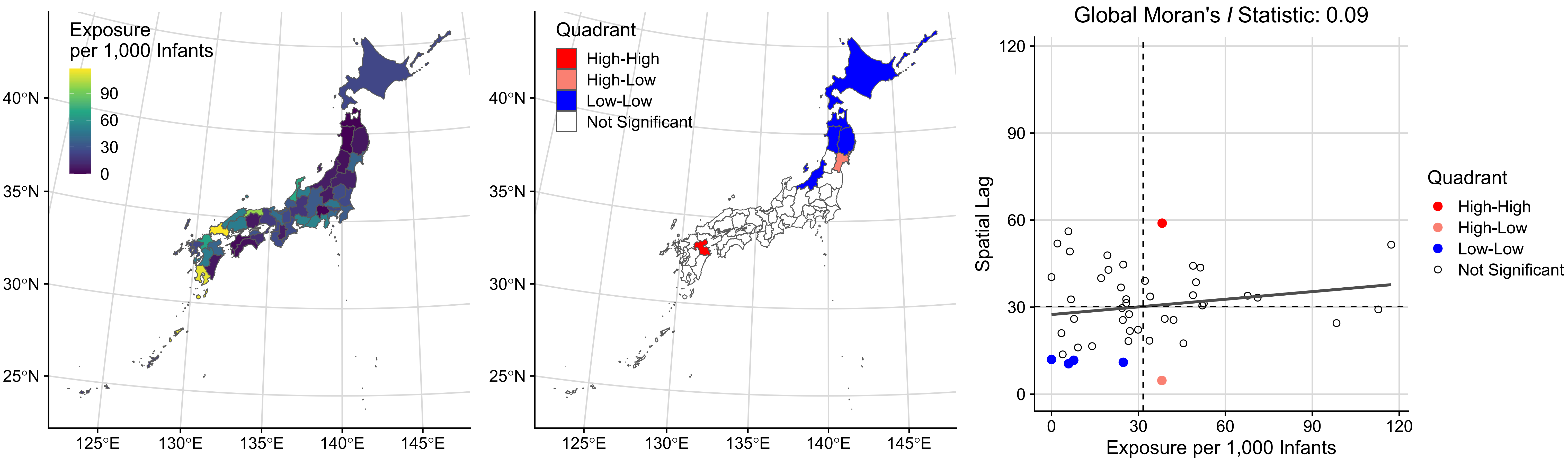

J01DH04. Doripenem

Early Neonatal Exposure among Very Preterm and Very Low Birth Weight Infants (Days 0–6)

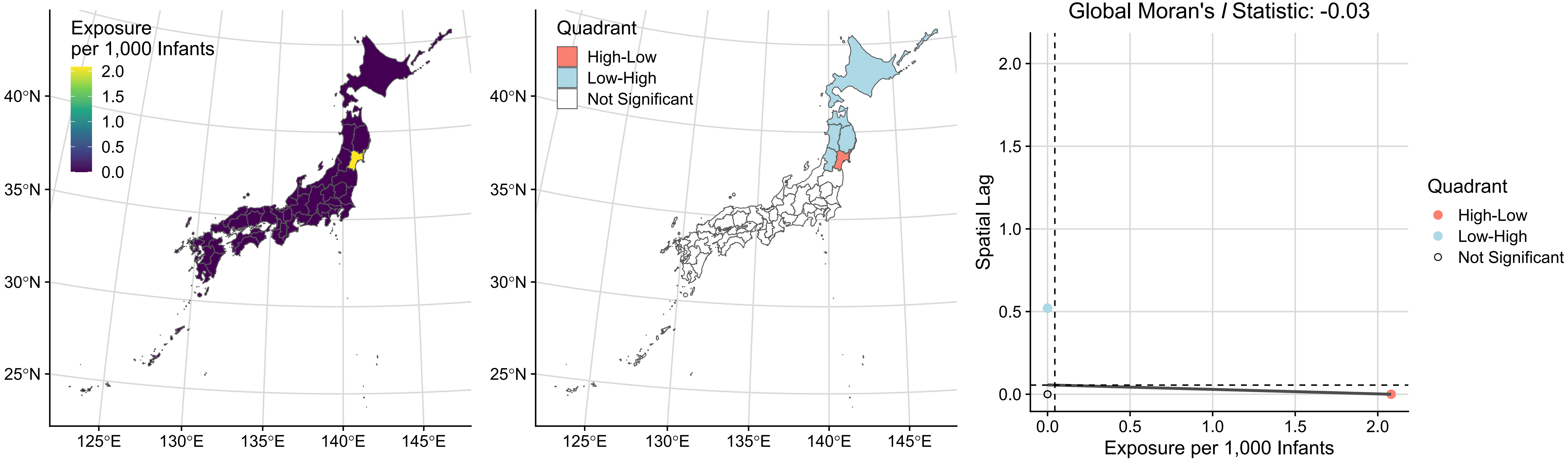

Neonatal Exposure among Very Preterm and Very Low Birth Weight Infants (Days 0–27)

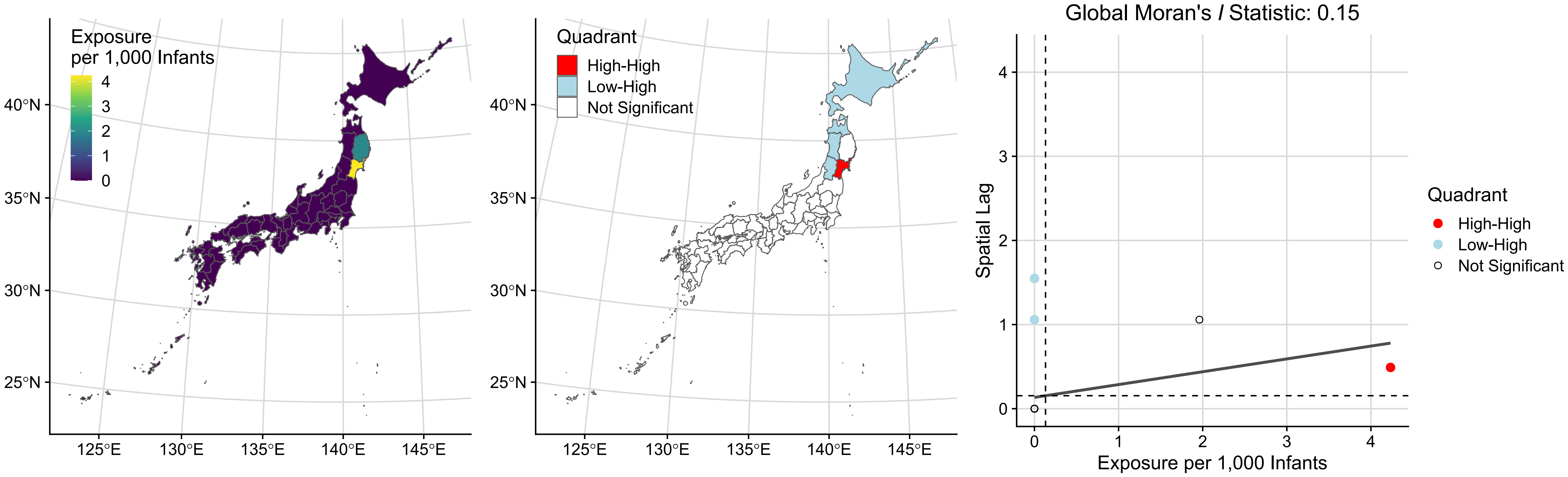

J01DH05. Biapenem

Early Neonatal Exposure among Very Preterm and Very Low Birth Weight Infants (Days 0–6)

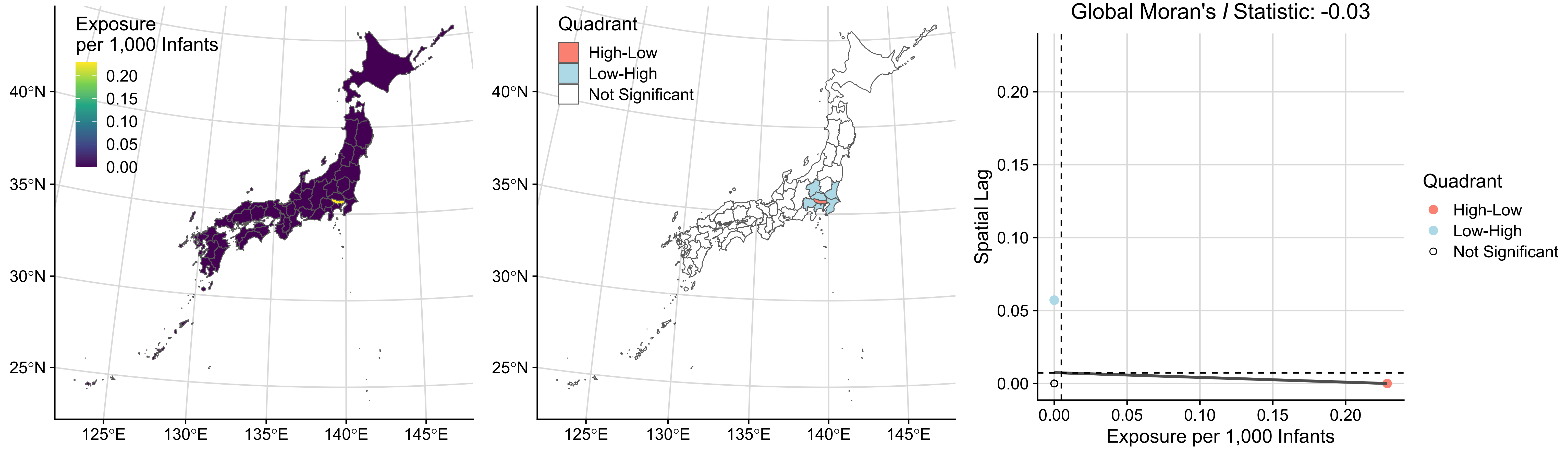

Neonatal Exposure among Very Preterm and Very Low Birth Weight Infants (Days 0–27)

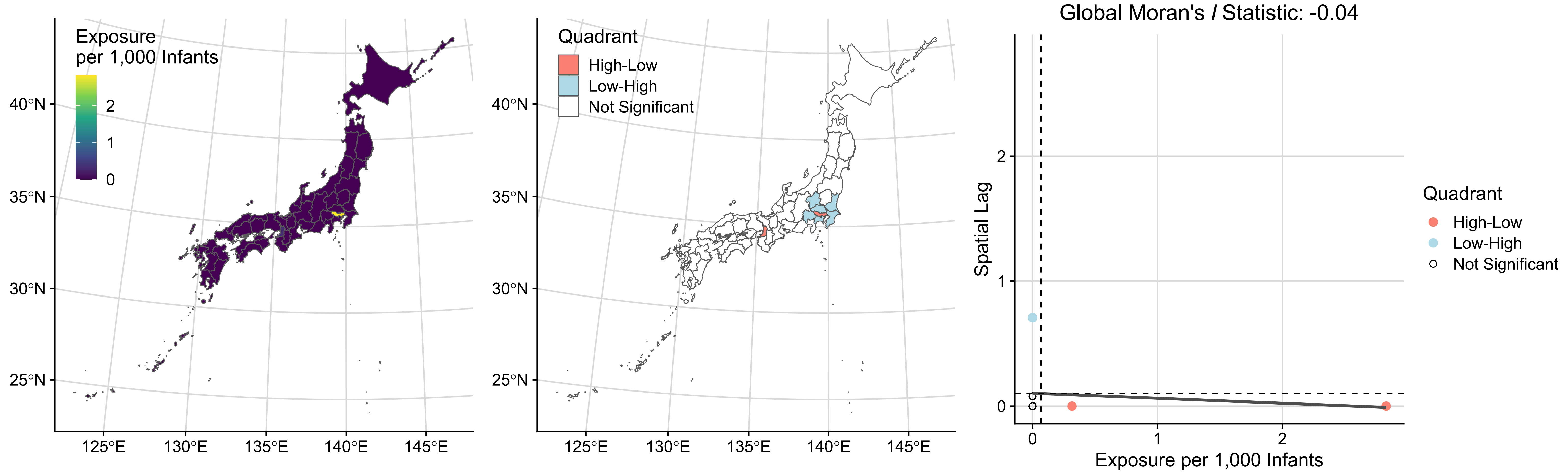

J01DH51. Imipenem and Cilastatin

Early Neonatal Exposure among Very Preterm and Very Low Birth Weight Infants (Days 0–6)

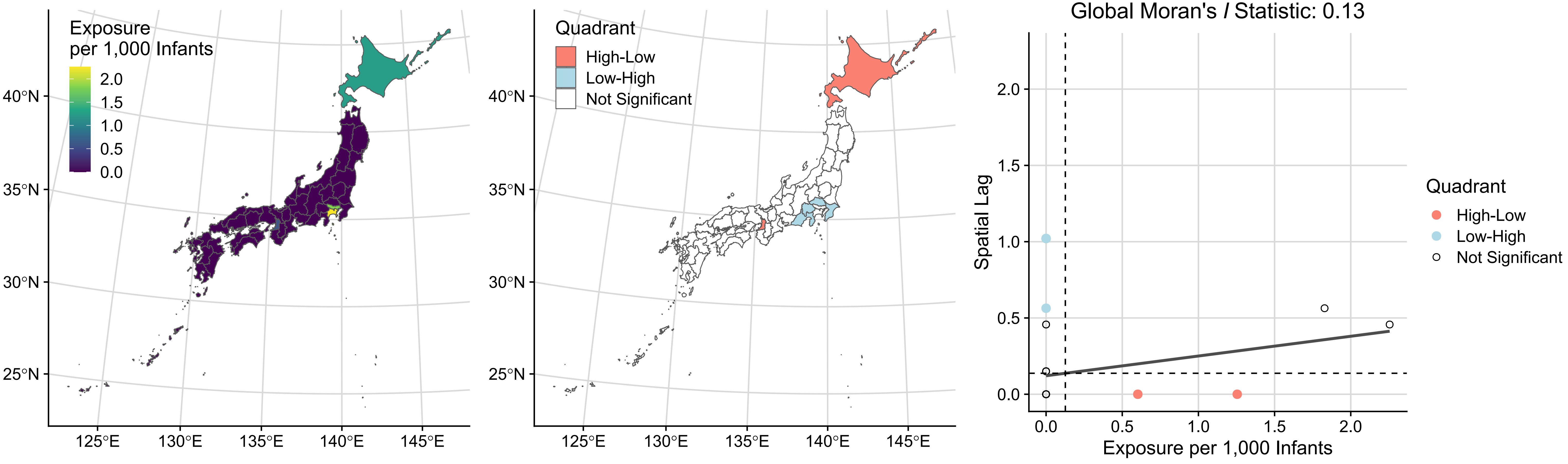

Neonatal Exposure among Very Preterm and Very Low Birth Weight Infants (Days 0–27)

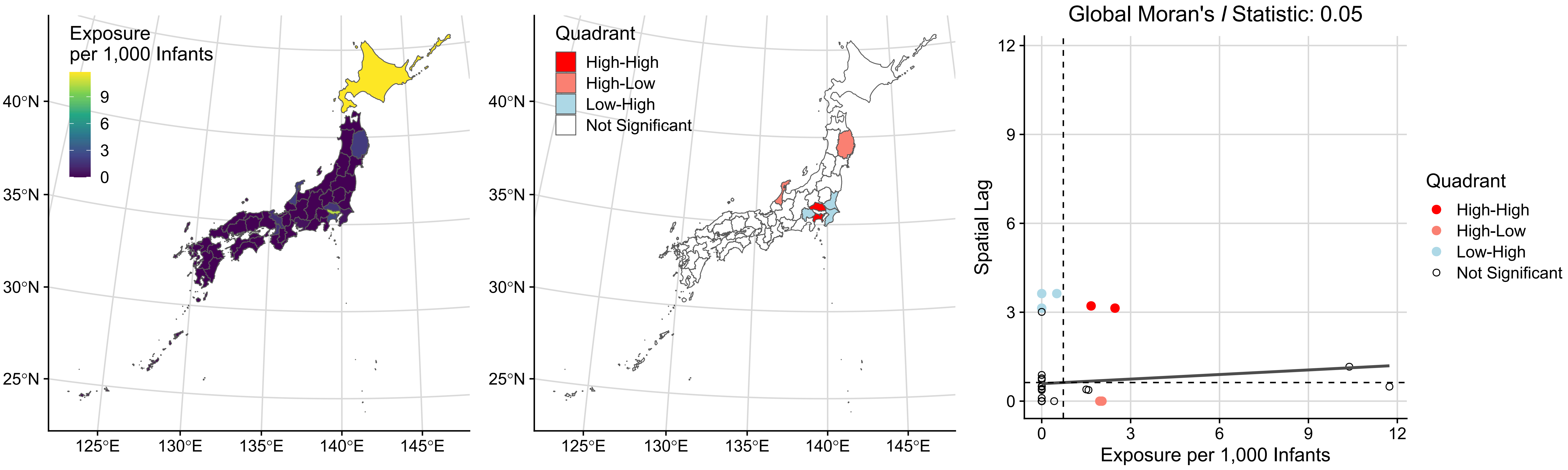

J01DH55. Panipenem and Betamipron

Early Neonatal Exposure among Very Preterm and Very Low Birth Weight Infants (Days 0–6)

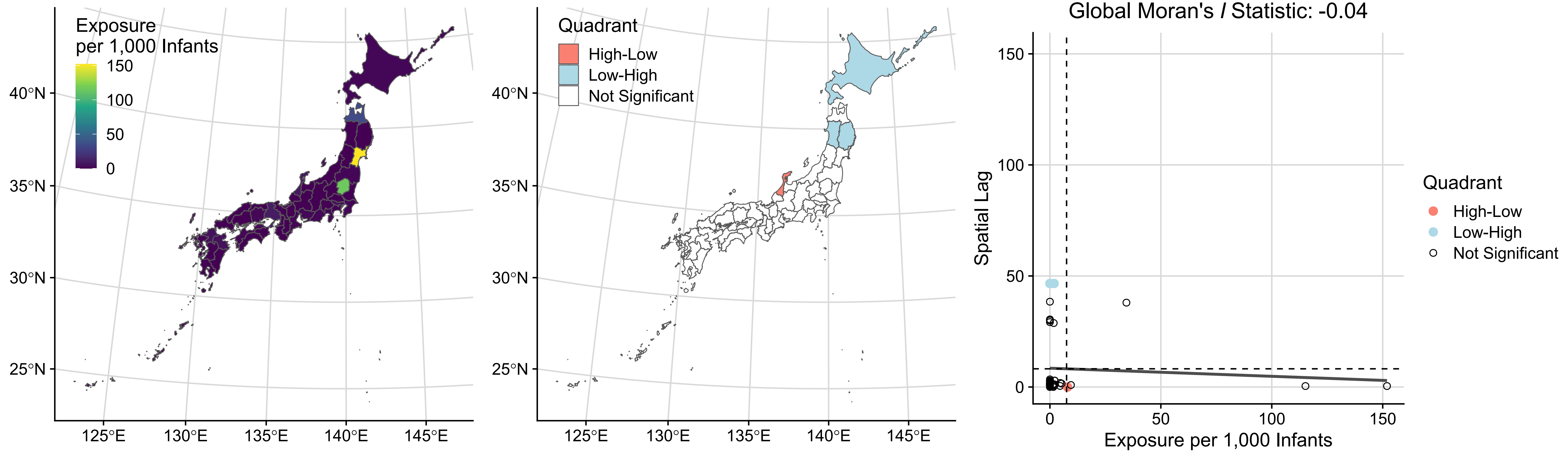

Neonatal Exposure among Very Preterm and Very Low Birth Weight Infants (Days 0–27)

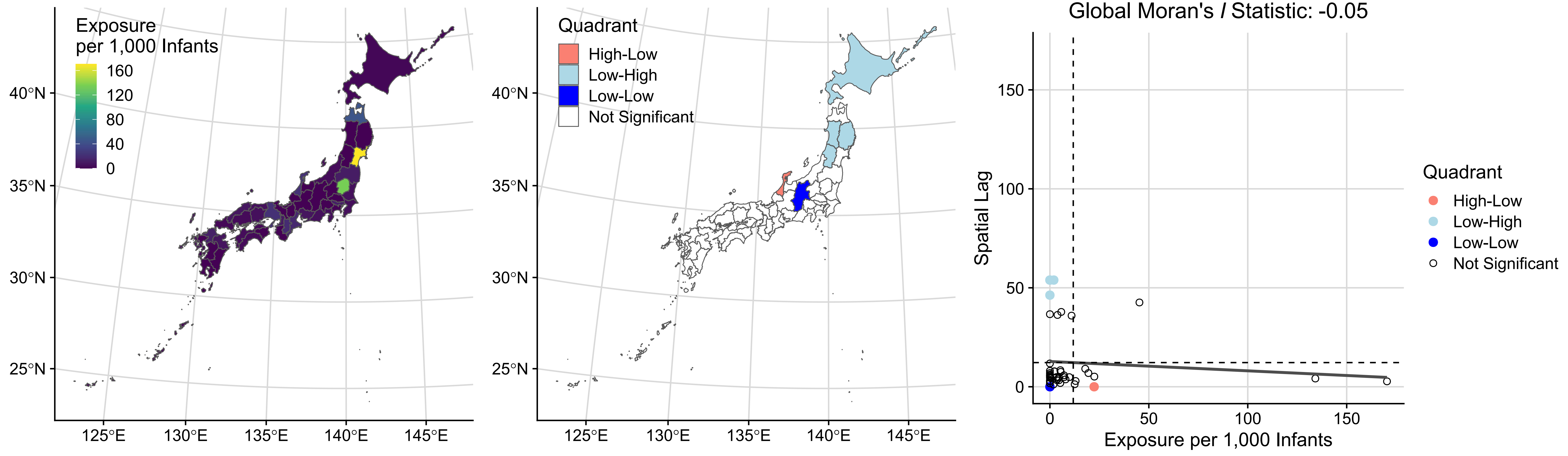

J01EE01. Sulfamethoxazole and Trimethoprim

Early Neonatal Exposure among Very Preterm and Very Low Birth Weight Infants (Days 0–6)

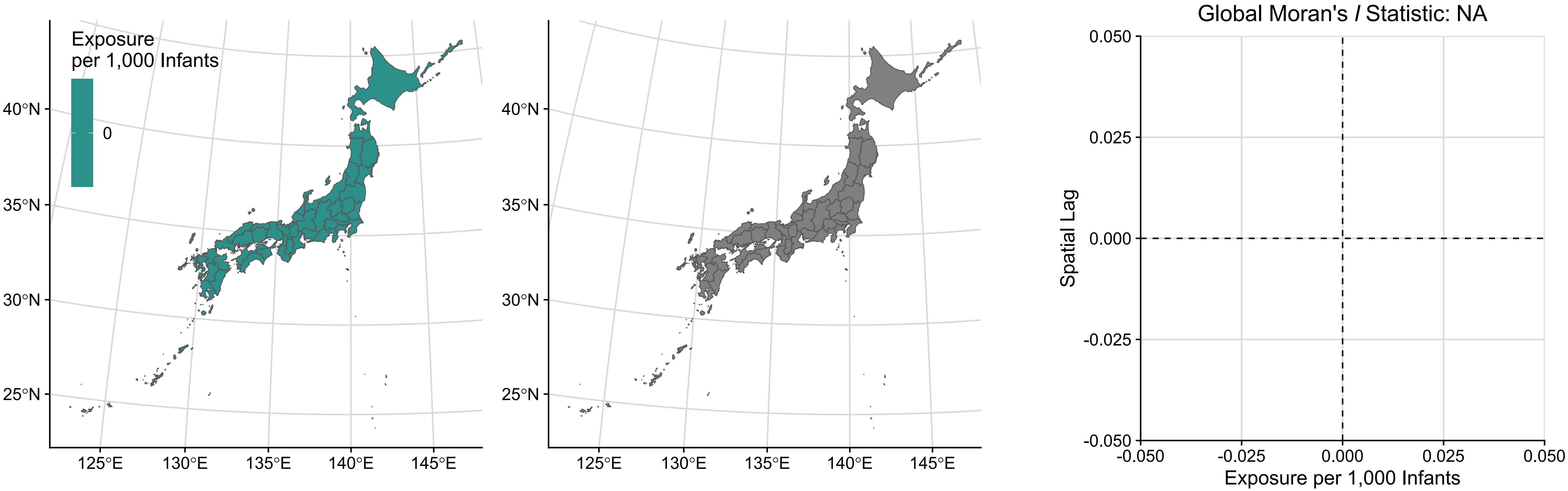

Neonatal Exposure among Very Preterm and Very Low Birth Weight Infants (Days 0–27)

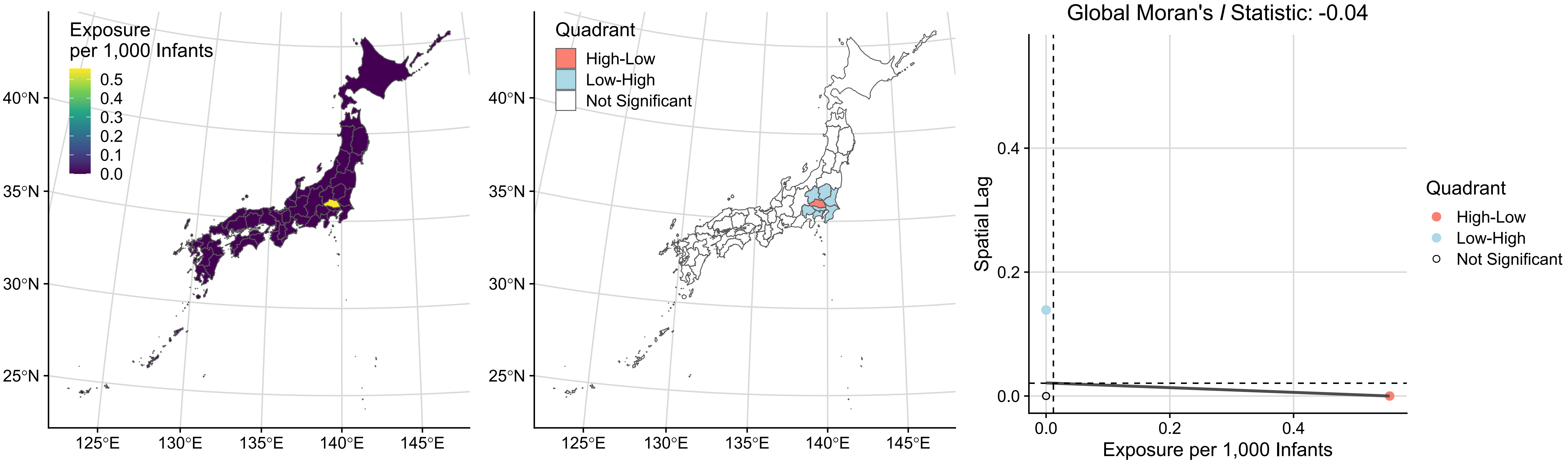

J01FA01. Erythromycin

Early Neonatal Exposure among Very Preterm and Very Low Birth Weight Infants (Days 0–6)

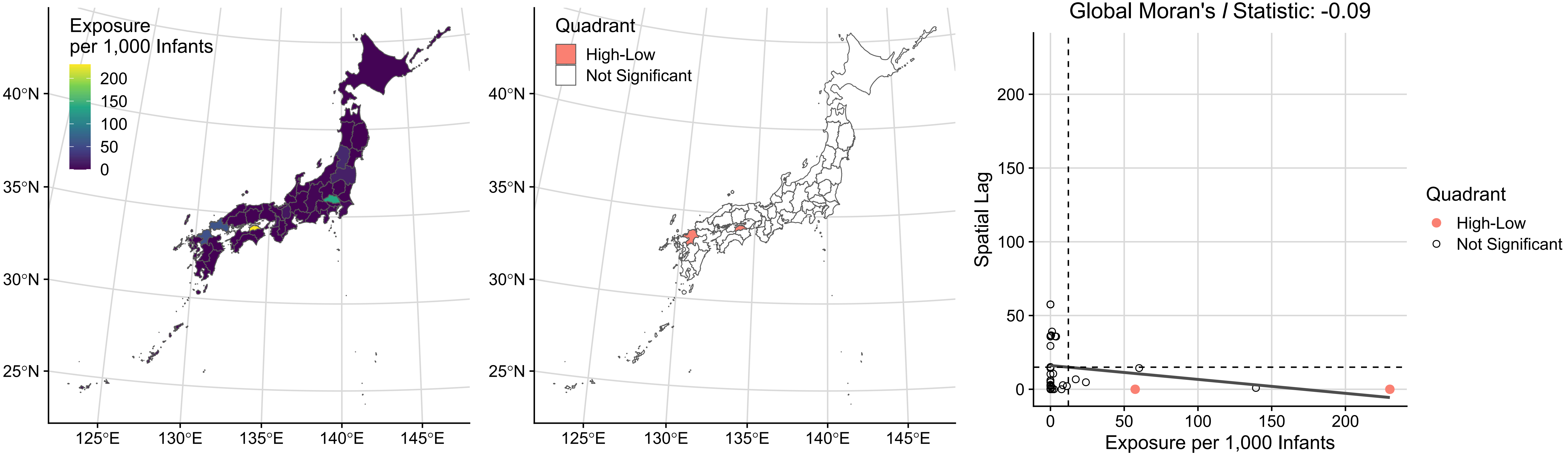

Neonatal Exposure among Very Preterm and Very Low Birth Weight Infants (Days 0–27)

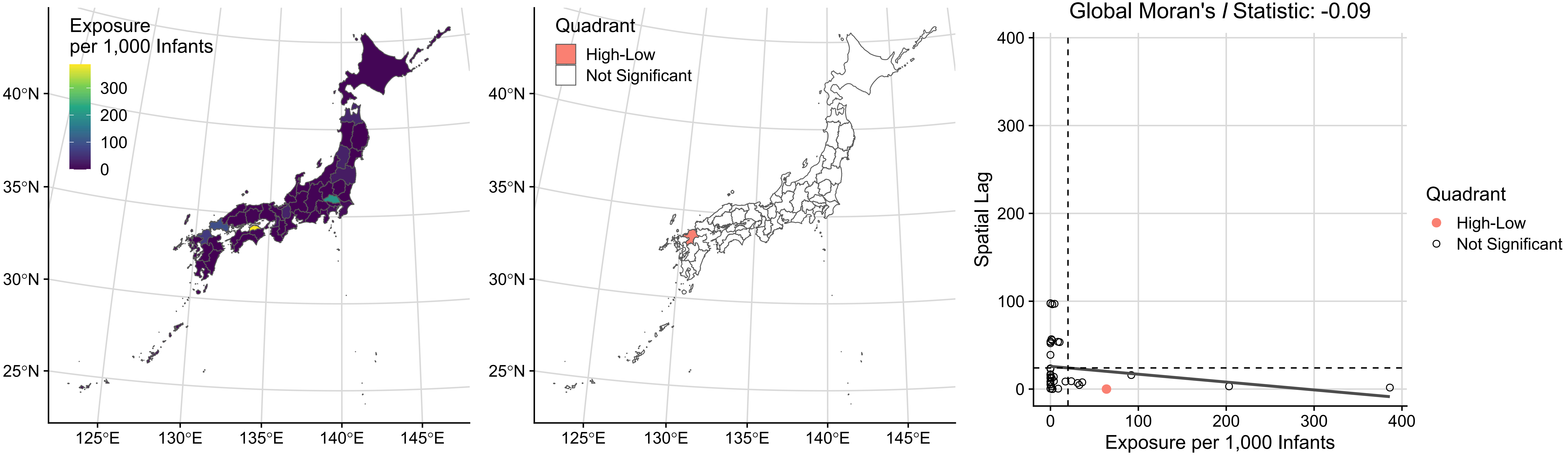

J01FA10. Azithromycin

Early Neonatal Exposure among Very Preterm and Very Low Birth Weight Infants (Days 0–6)

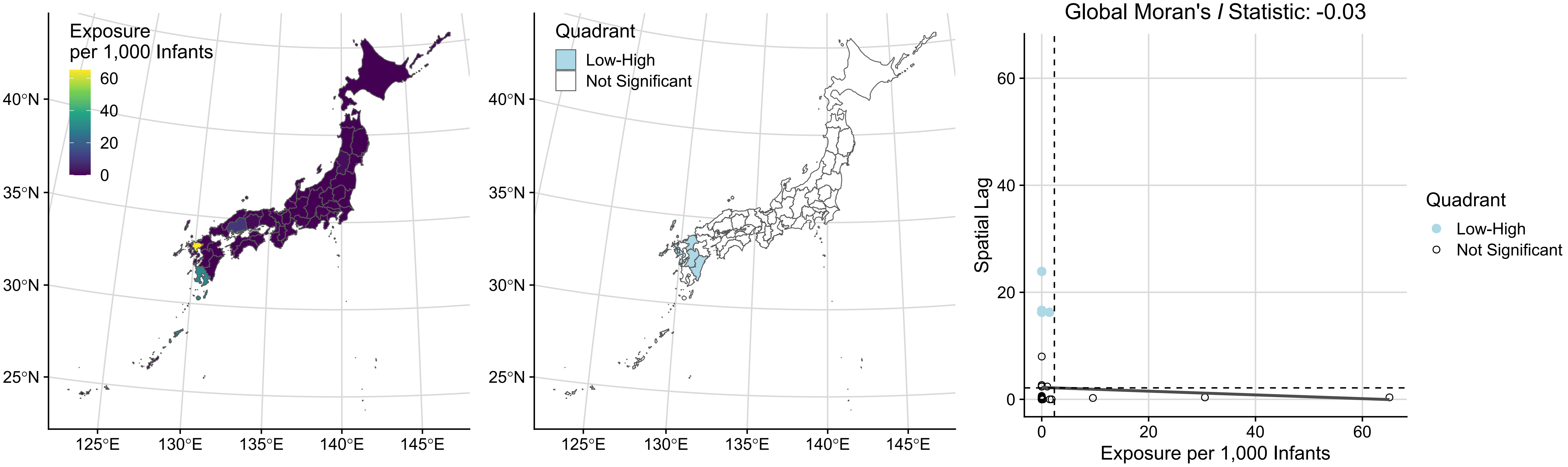

Neonatal Exposure among Very Preterm and Very Low Birth Weight Infants (Days 0–27)

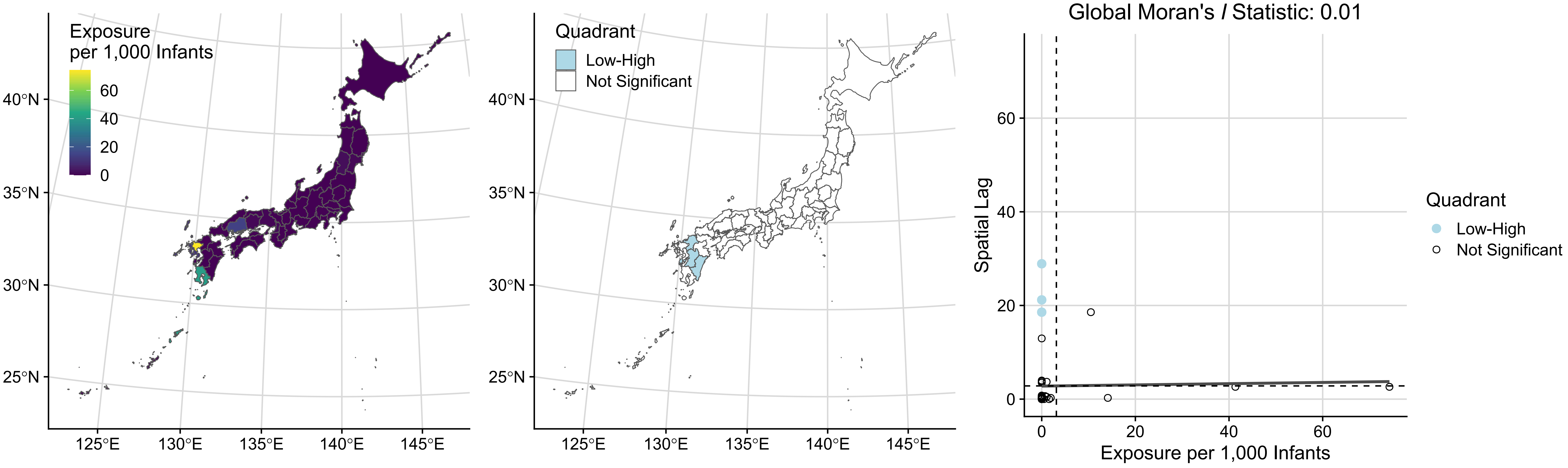

J01FF01. Clindamycin

Early Neonatal Exposure among Very Preterm and Very Low Birth Weight Infants (Days 0–6)

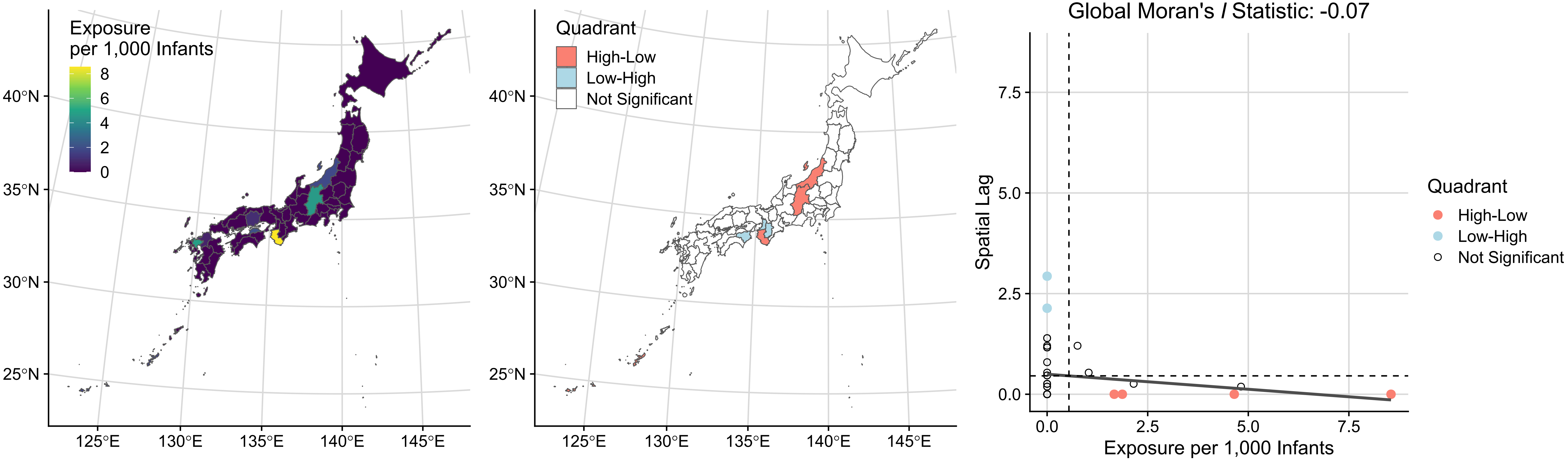

Neonatal Exposure among Very Preterm and Very Low Birth Weight Infants (Days 0–27)

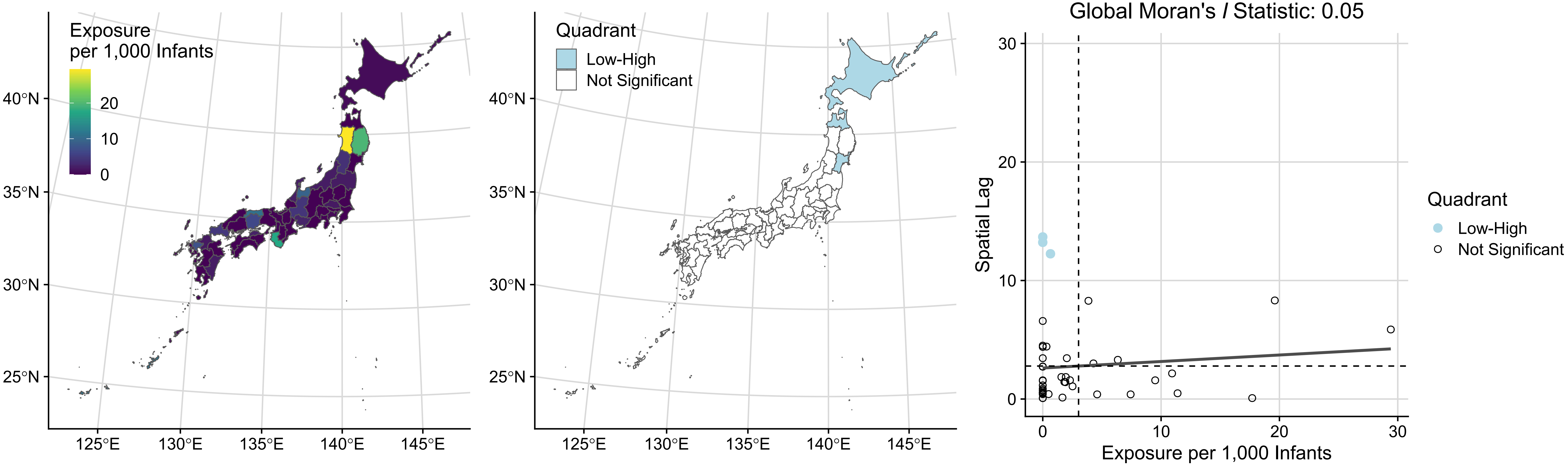

J01GB01. Tobramycin

Early Neonatal Exposure among Very Preterm and Very Low Birth Weight Infants (Days 0–6)

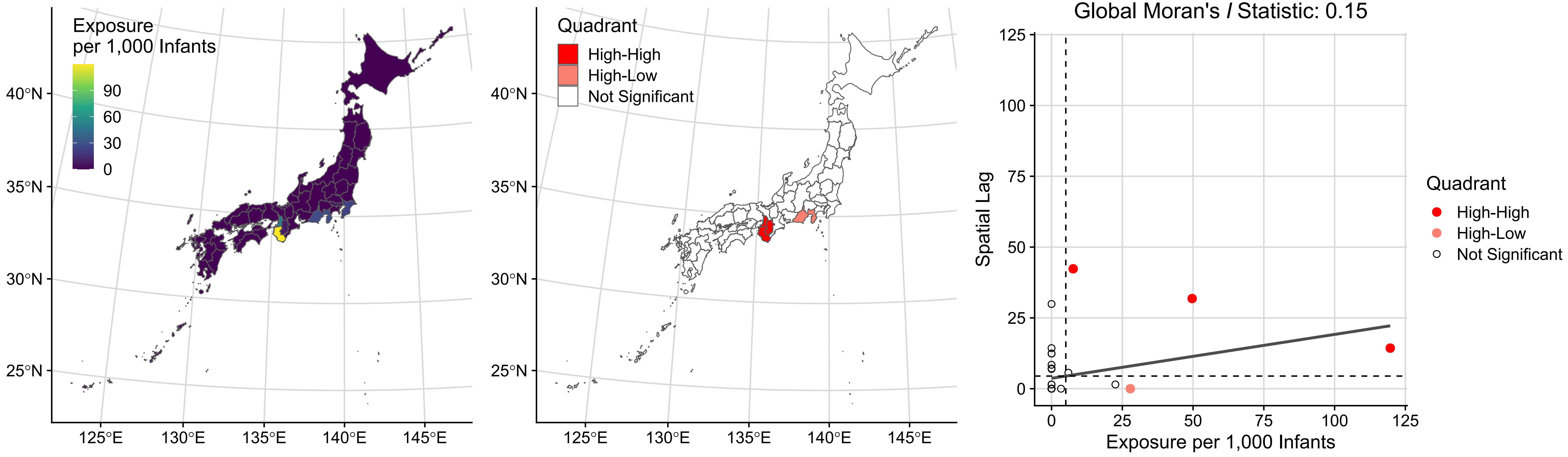

Neonatal Exposure among Very Preterm and Very Low Birth Weight Infants (Days 0–27)

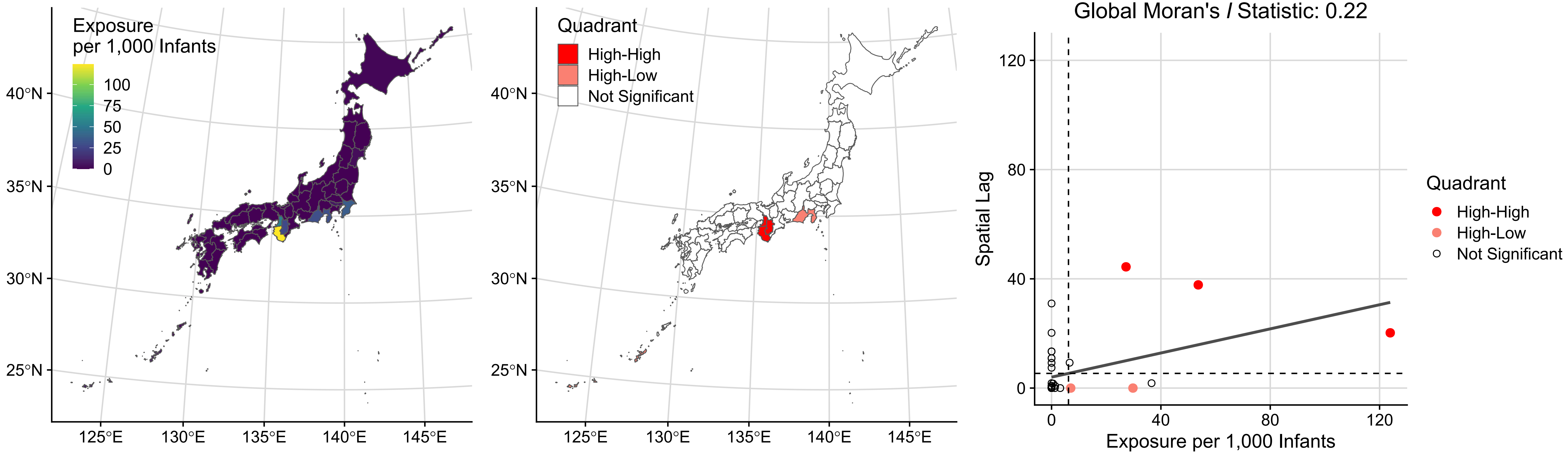

J01GB03. Gentamicin

Early Neonatal Exposure among Very Preterm and Very Low Birth Weight Infants (Days 0–6)

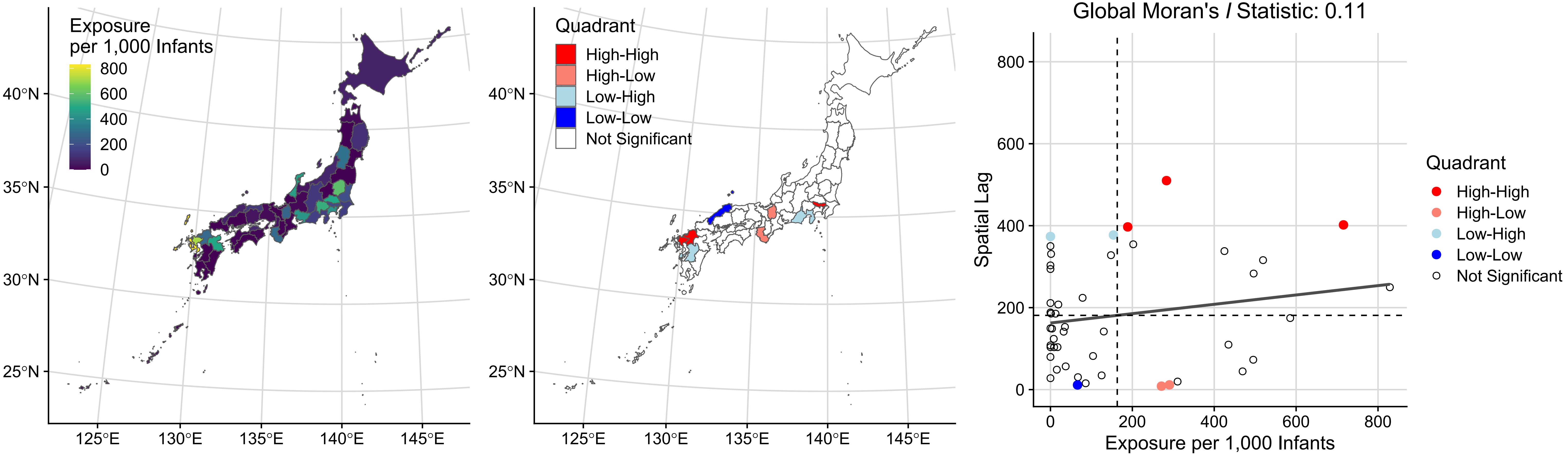

Neonatal Exposure among Very Preterm and Very Low Birth Weight Infants (Days 0–27)

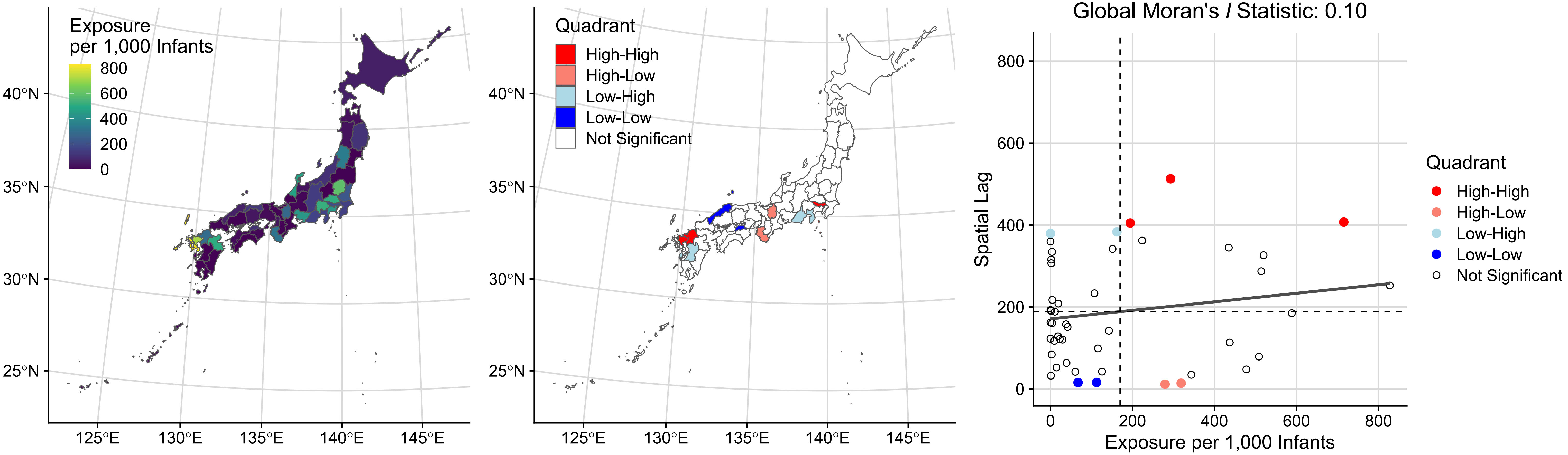

J01GB06. Amikacin

Early Neonatal Exposure among Very Preterm and Very Low Birth Weight Infants (Days 0–6)

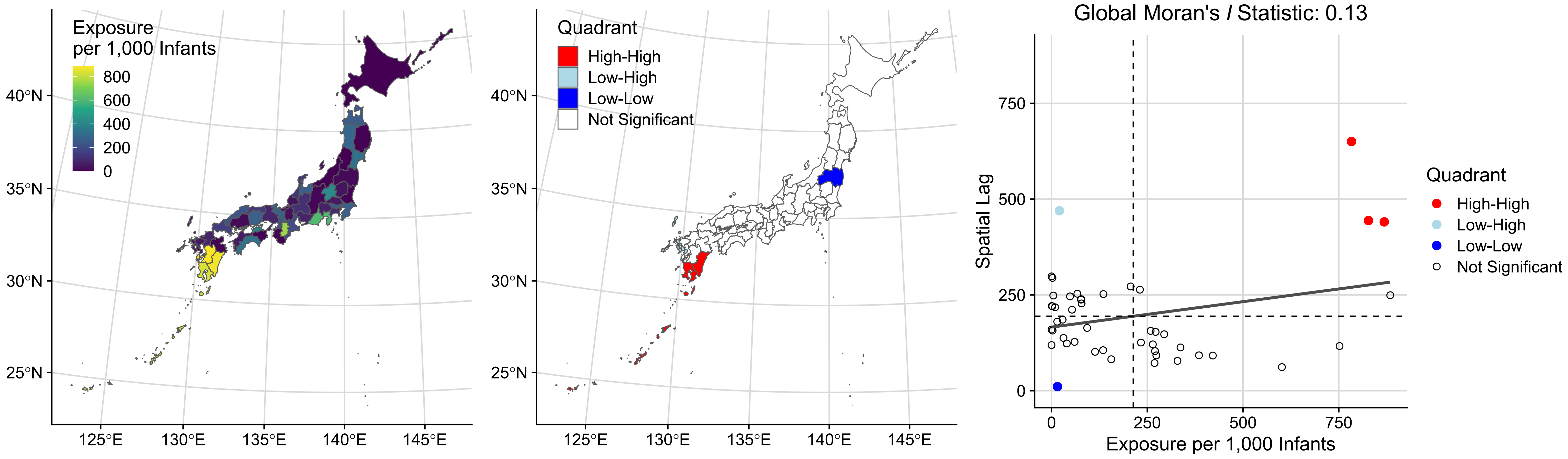

Neonatal Exposure among Very Preterm and Very Low Birth Weight Infants (Days 0–27)

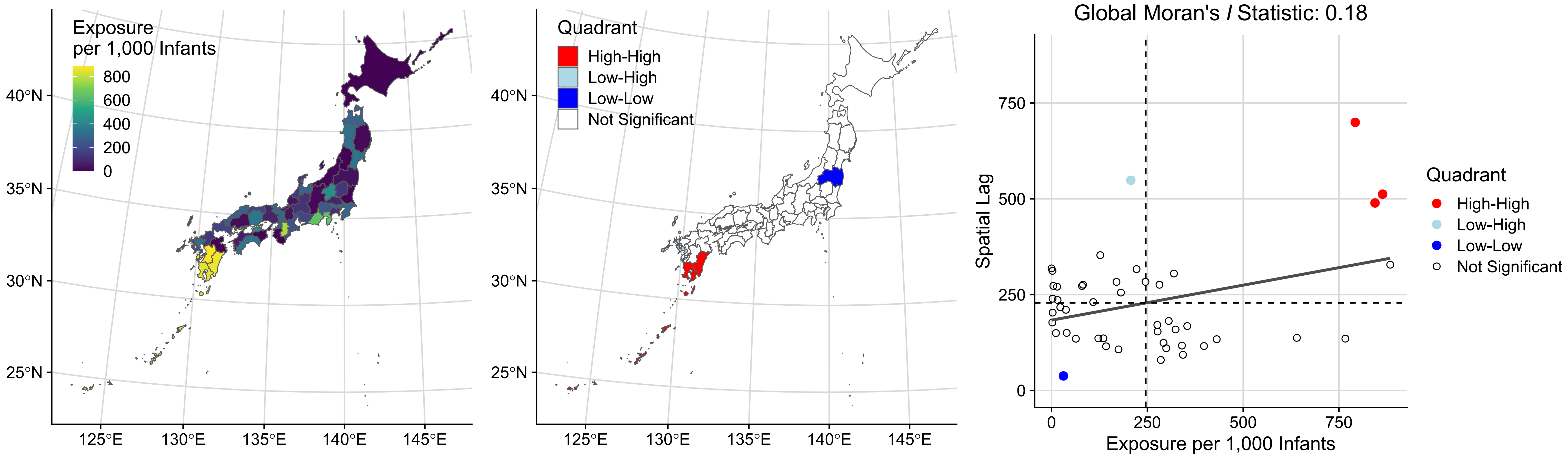

J01GB11. Isepamicin

Early Neonatal Exposure among Very Preterm and Very Low Birth Weight Infants (Days 0–6)

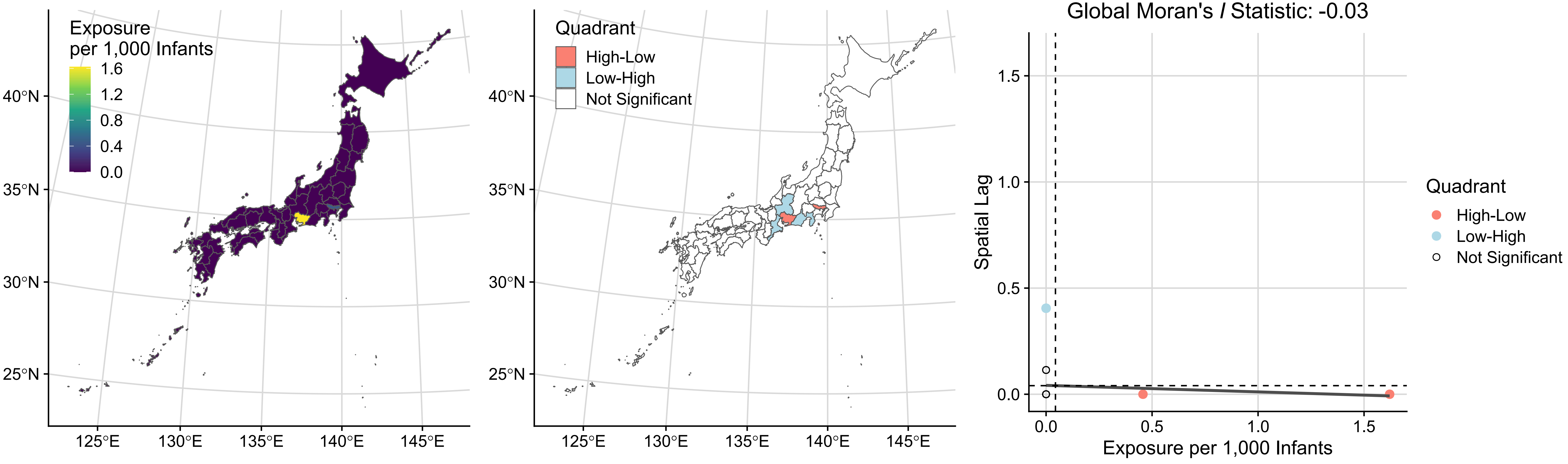

Neonatal Exposure among Very Preterm and Very Low Birth Weight Infants (Days 0–27)

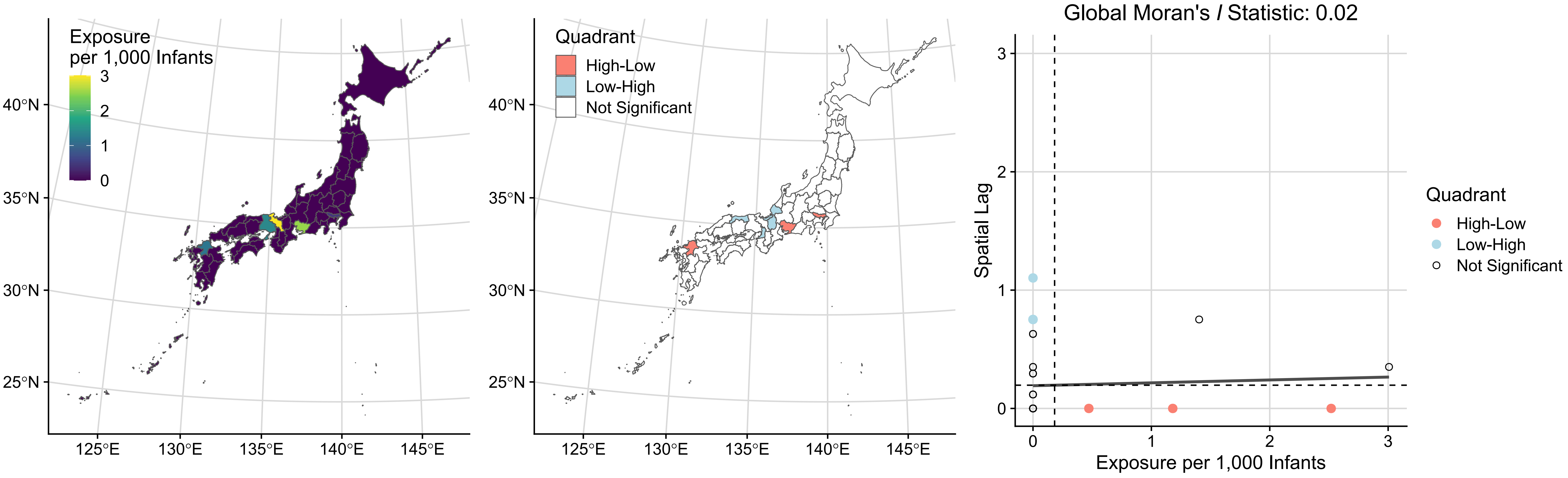

J01GB12. Arbekacin

Early Neonatal Exposure among Very Preterm and Very Low Birth Weight Infants (Days 0–6)

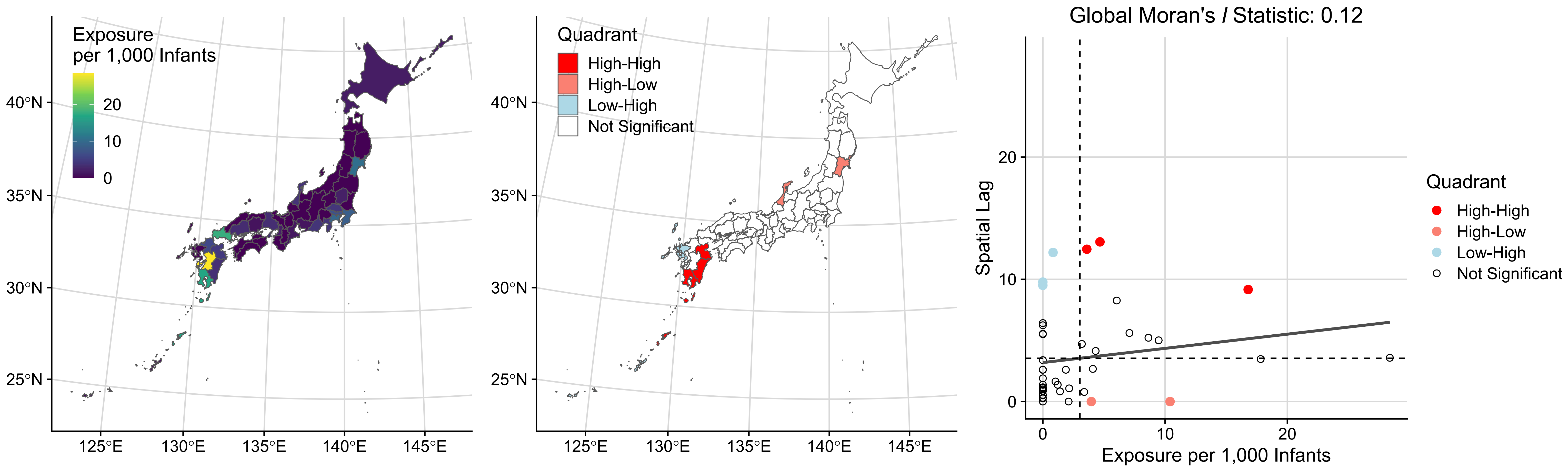

Neonatal Exposure among Very Preterm and Very Low Birth Weight Infants (Days 0–27)

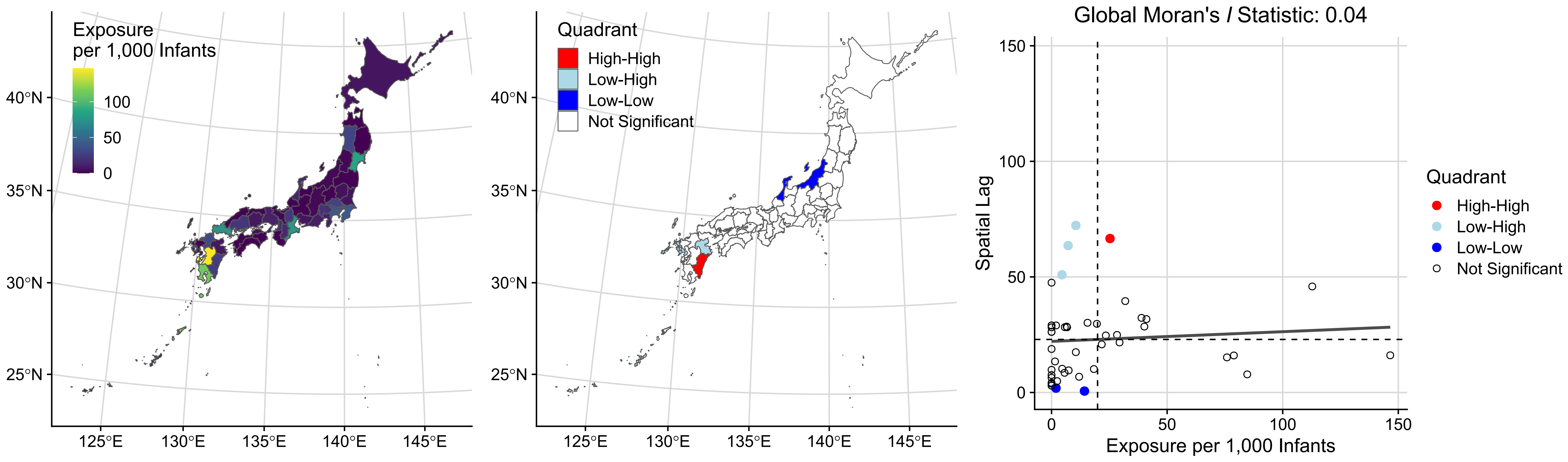

J01MA02. Ciprofloxacin

Early Neonatal Exposure among Very Preterm and Very Low Birth Weight Infants (Days 0–6)

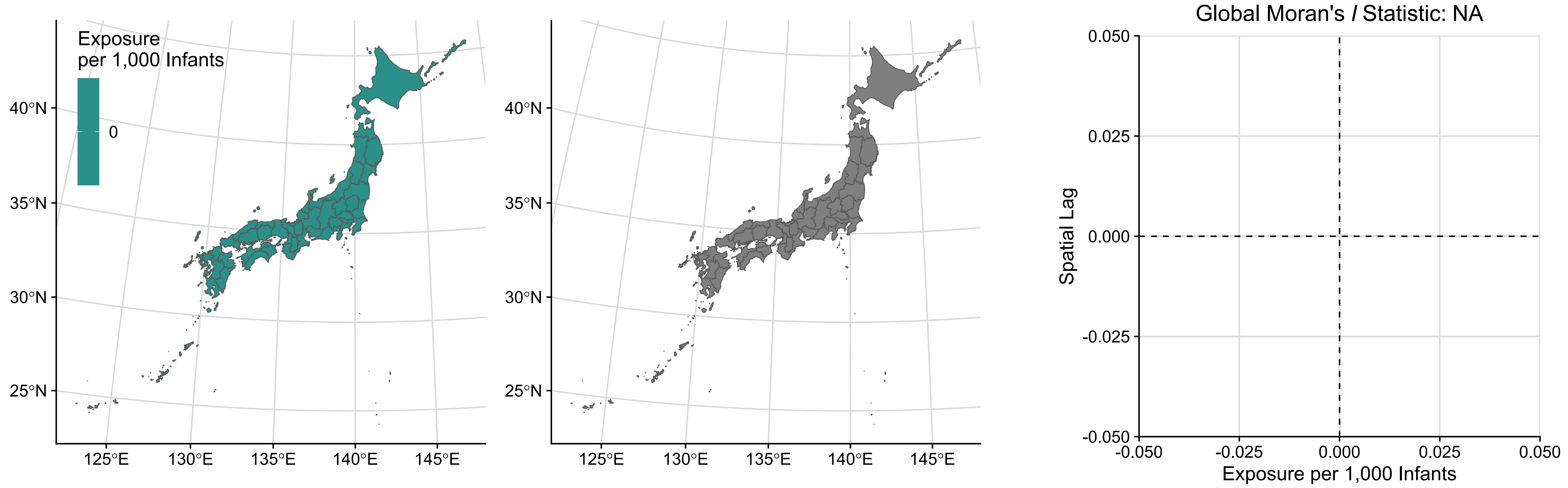

Neonatal Exposure among Very Preterm and Very Low Birth Weight Infants (Days 0–27)

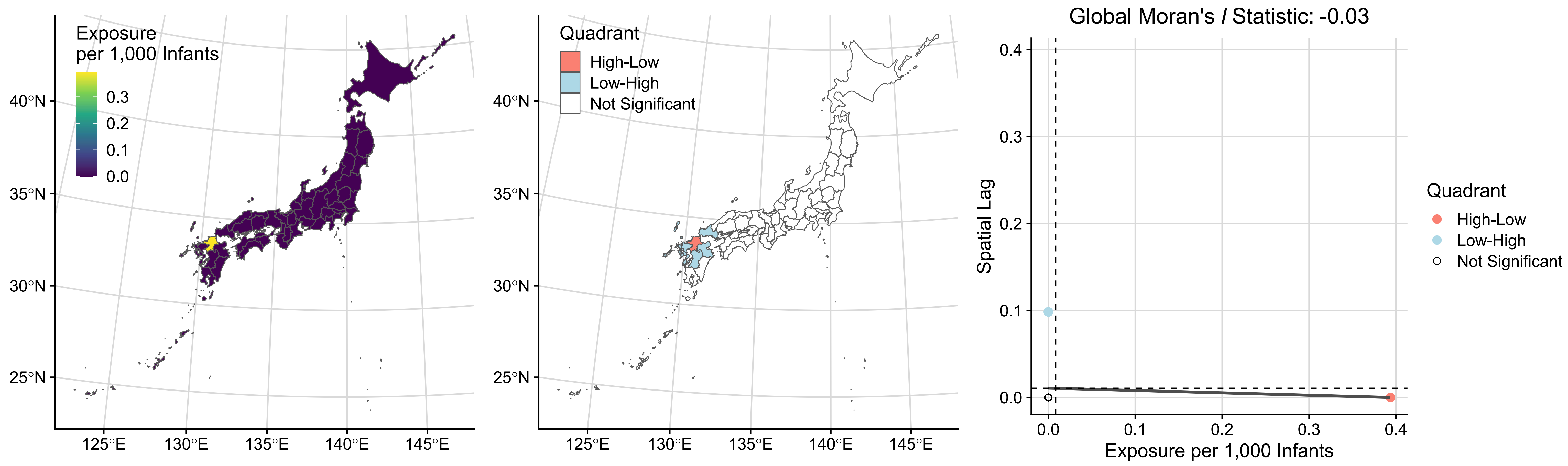

J01MA18. Pazufloxacin

Early Neonatal Exposure among Very Preterm and Very Low Birth Weight Infants (Days 0–6)

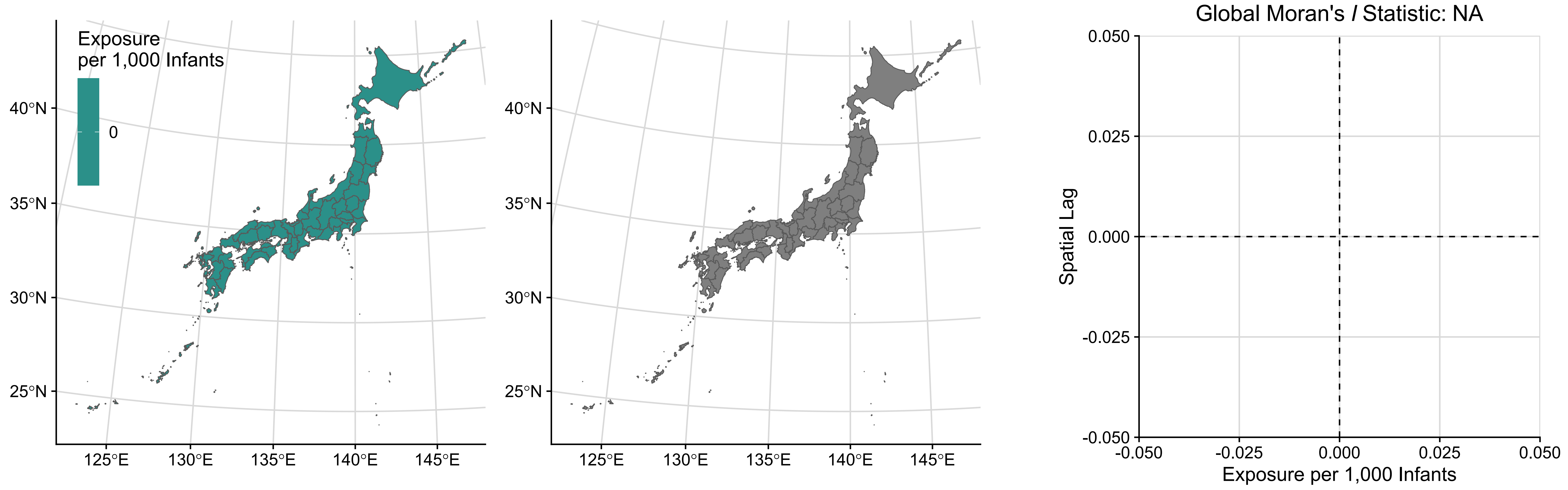

Neonatal Exposure among Very Preterm and Very Low Birth Weight Infants (Days 0–27)

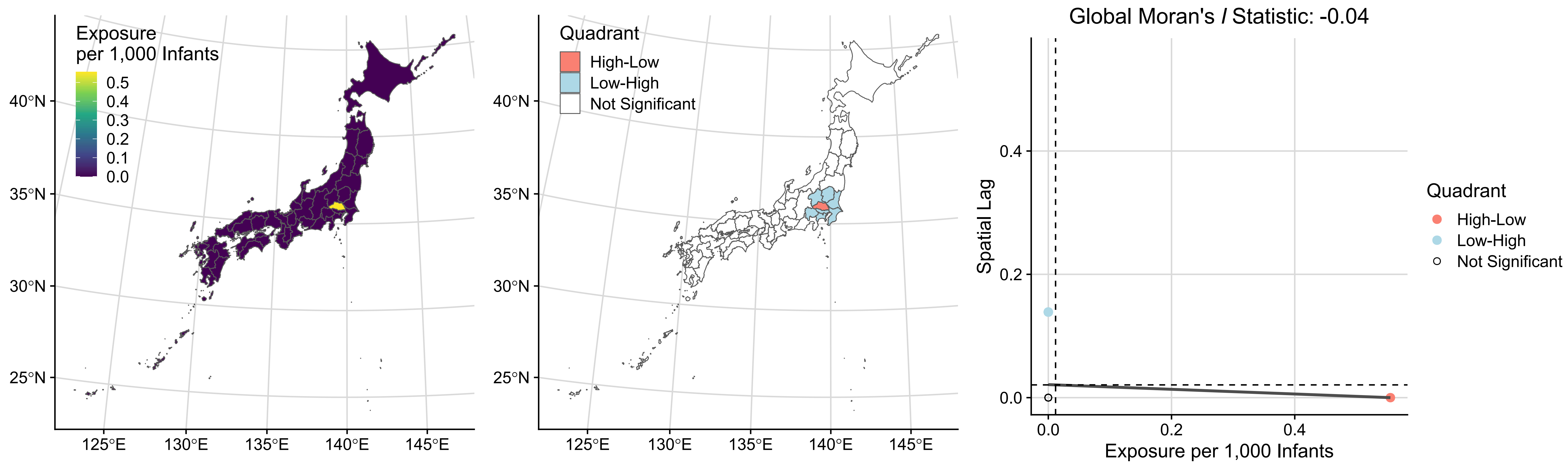

J01XA01. Vancomycin

Early Neonatal Exposure among Very Preterm and Very Low Birth Weight Infants (Days 0–6)

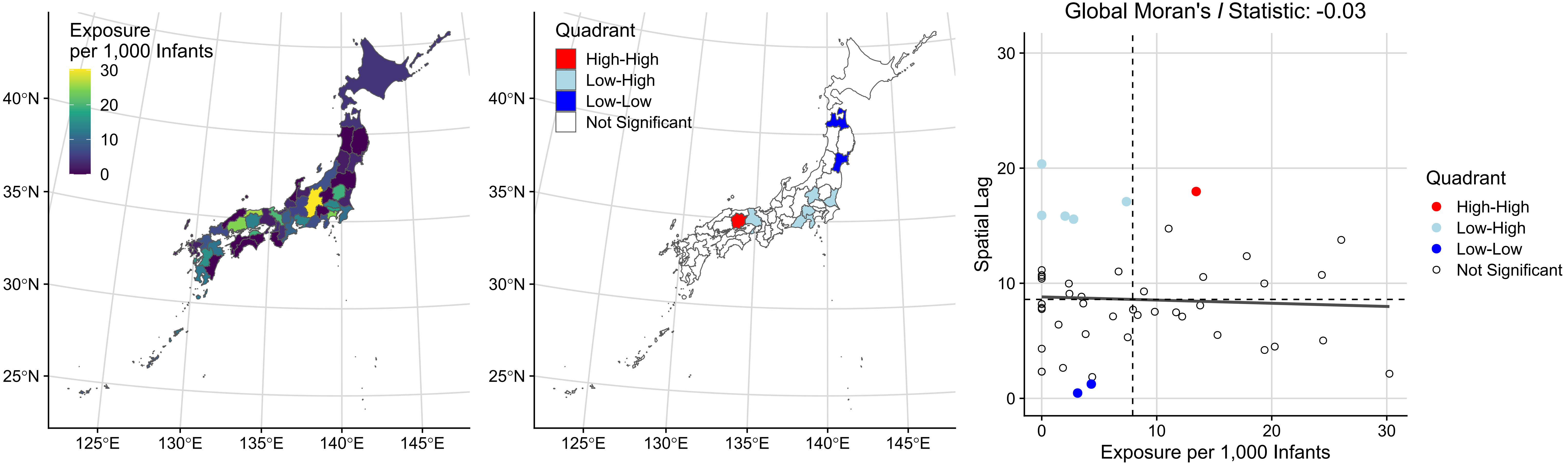

Neonatal Exposure among Very Preterm and Very Low Birth Weight Infants (Days 0–27)

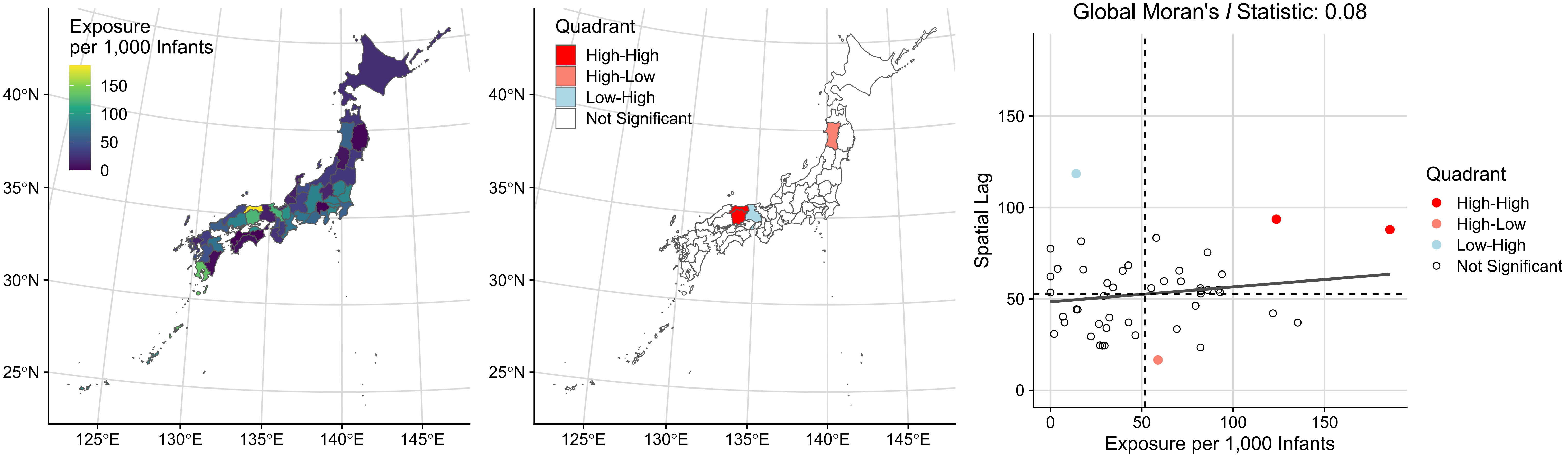

J01XA02. Teicoplanin

Early Neonatal Exposure among Very Preterm and Very Low Birth Weight Infants (Days 0–6)

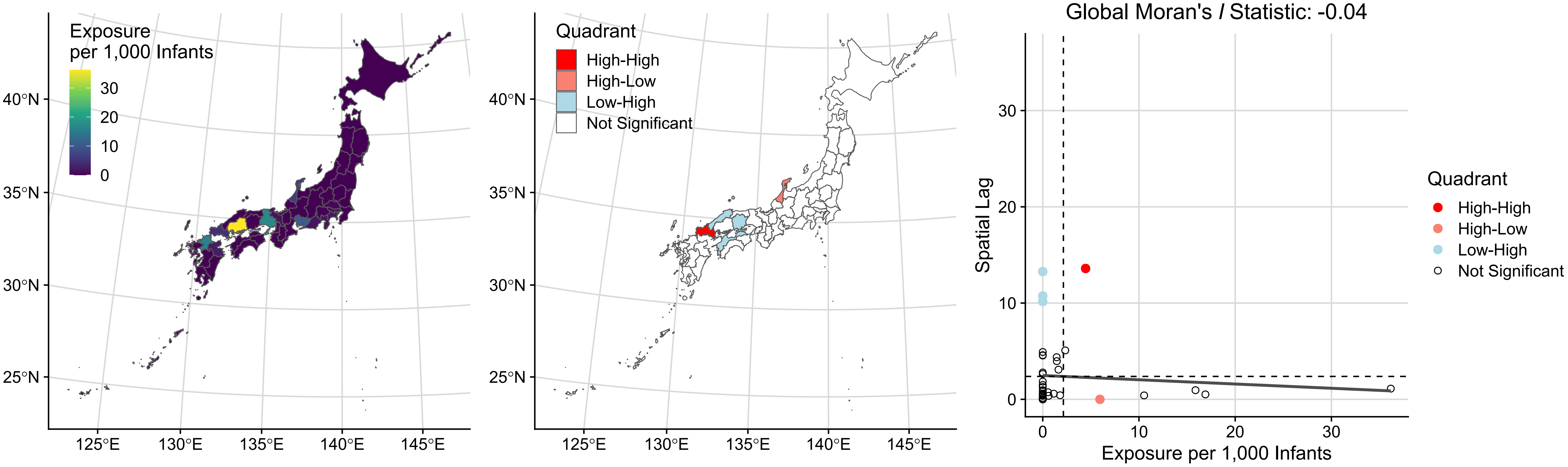

Neonatal Exposure among Very Preterm and Very Low Birth Weight Infants (Days 0–27)

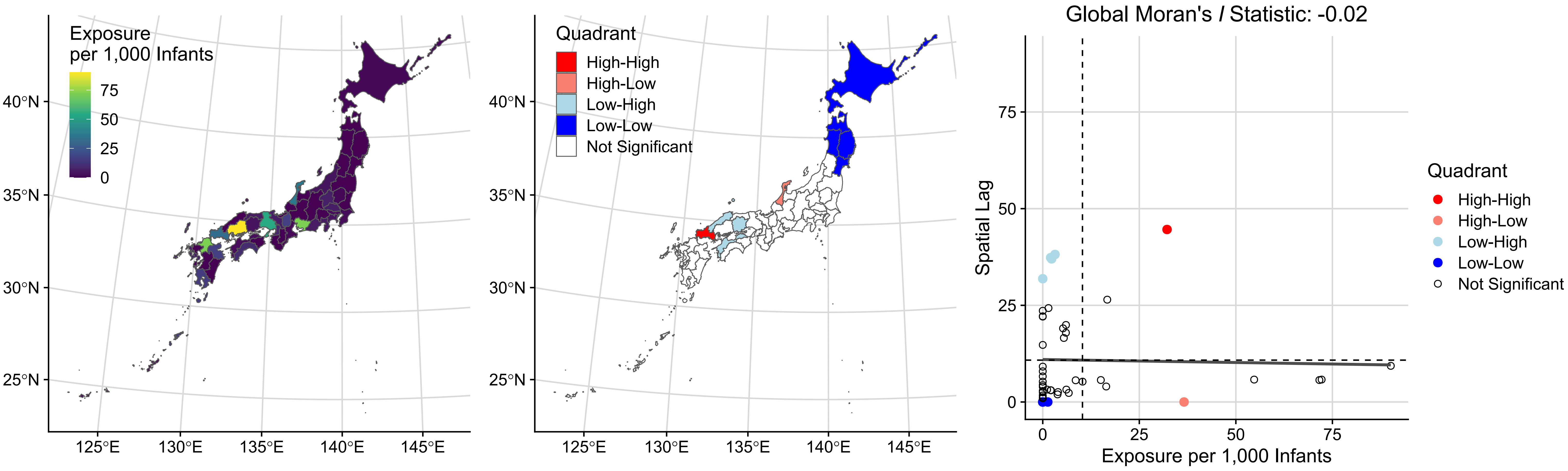

J01XD01. Metronidazole

Early Neonatal Exposure among Very Preterm and Very Low Birth Weight Infants (Days 0–6)

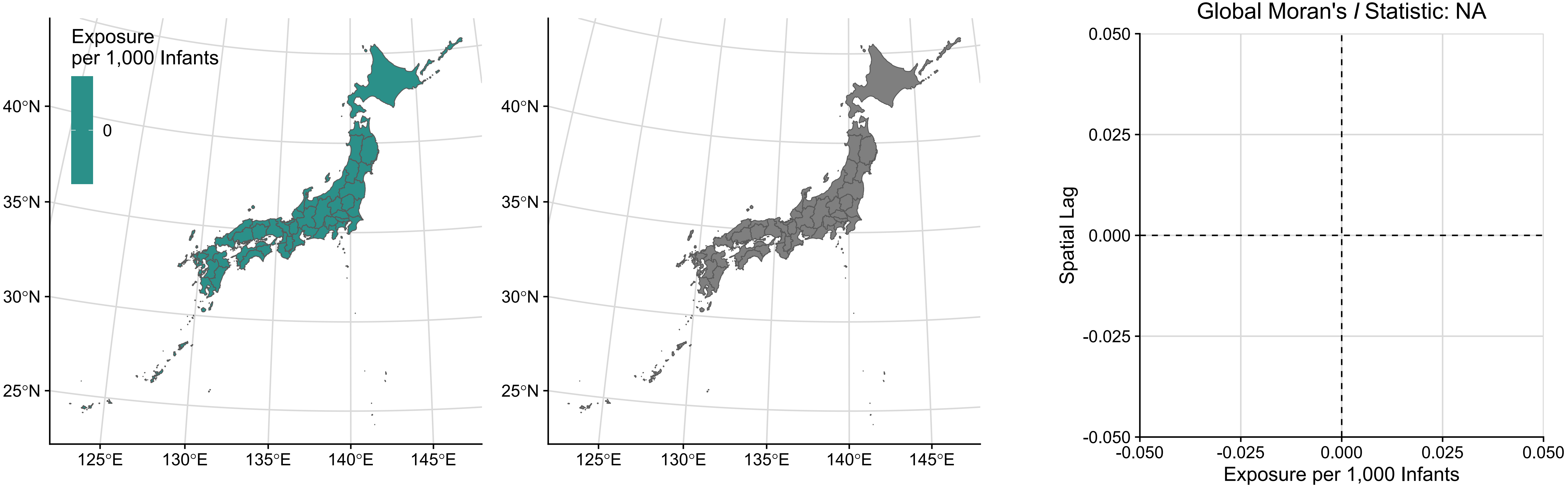

Neonatal Exposure among Very Preterm and Very Low Birth Weight Infants (Days 0–27)

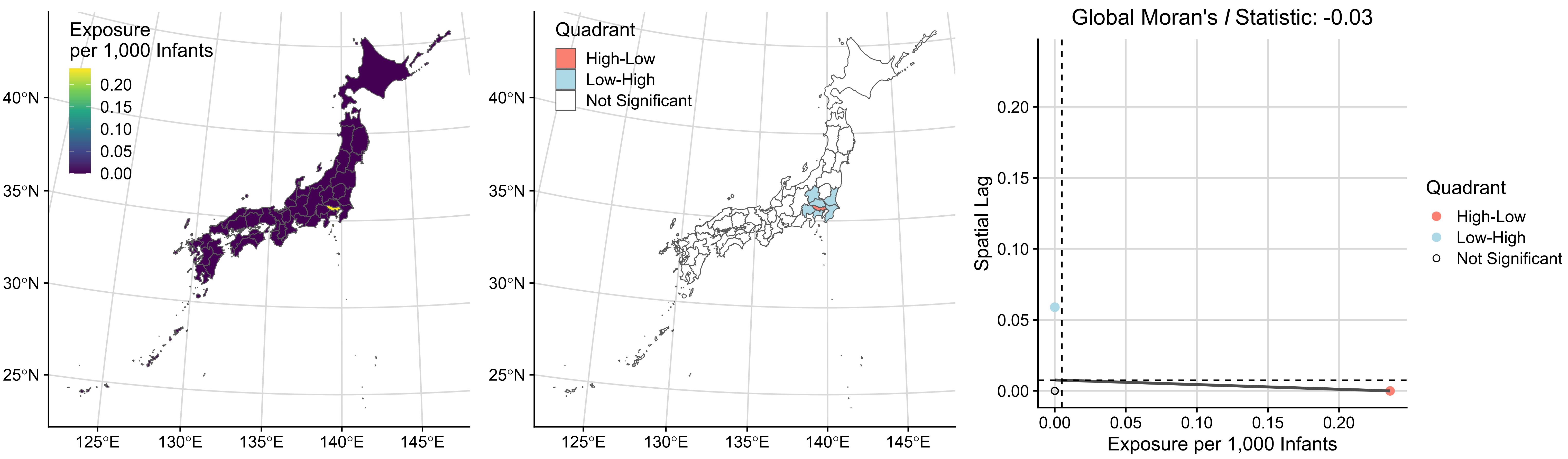

J01XX01. Fosfomycin

Early Neonatal Exposure among Very Preterm and Very Low Birth Weight Infants (Days 0–6)

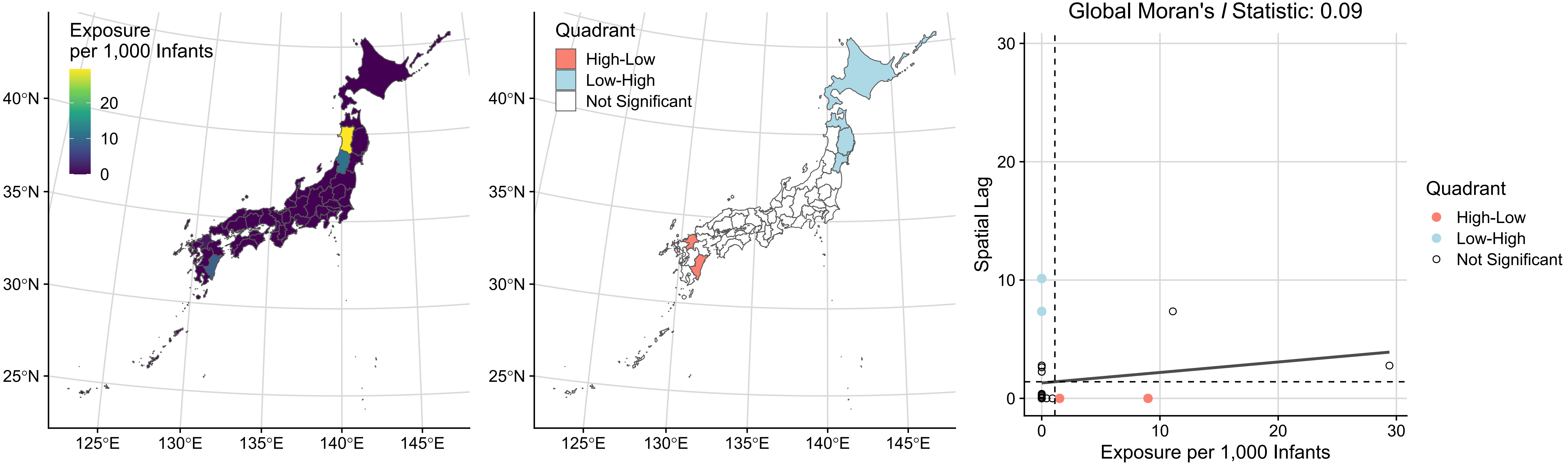

Neonatal Exposure among Very Preterm and Very Low Birth Weight Infants (Days 0–27)

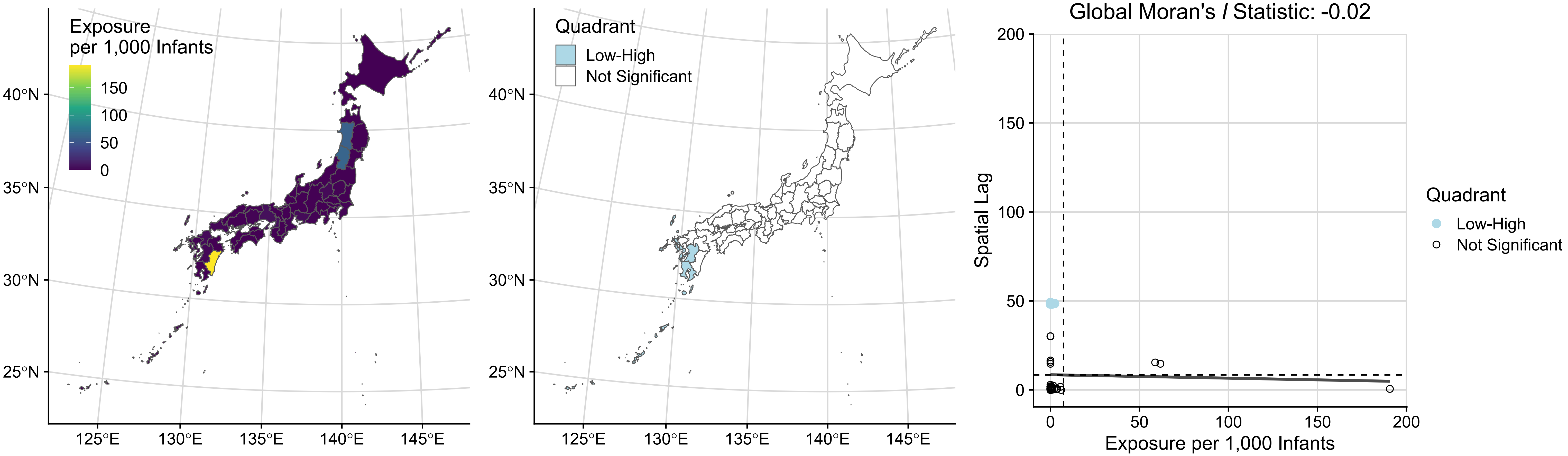

J01XX08. Linezolid

Early Neonatal Exposure among Very Preterm and Very Low Birth Weight Infants (Days 0–6)

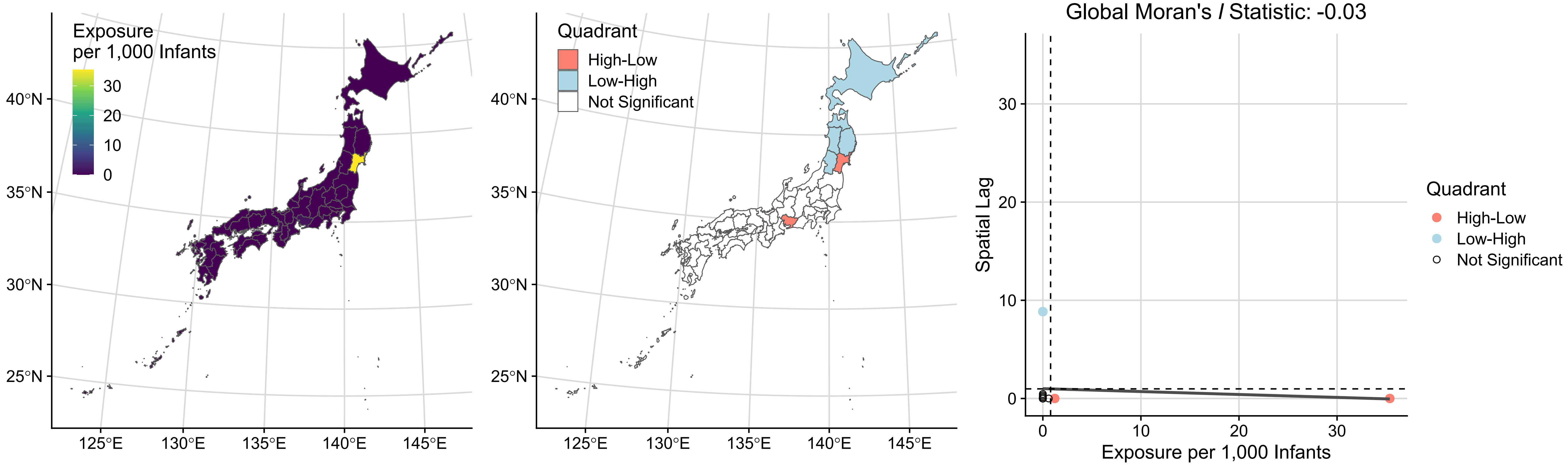

Neonatal Exposure among Very Preterm and Very Low Birth Weight Infants (Days 0–27)

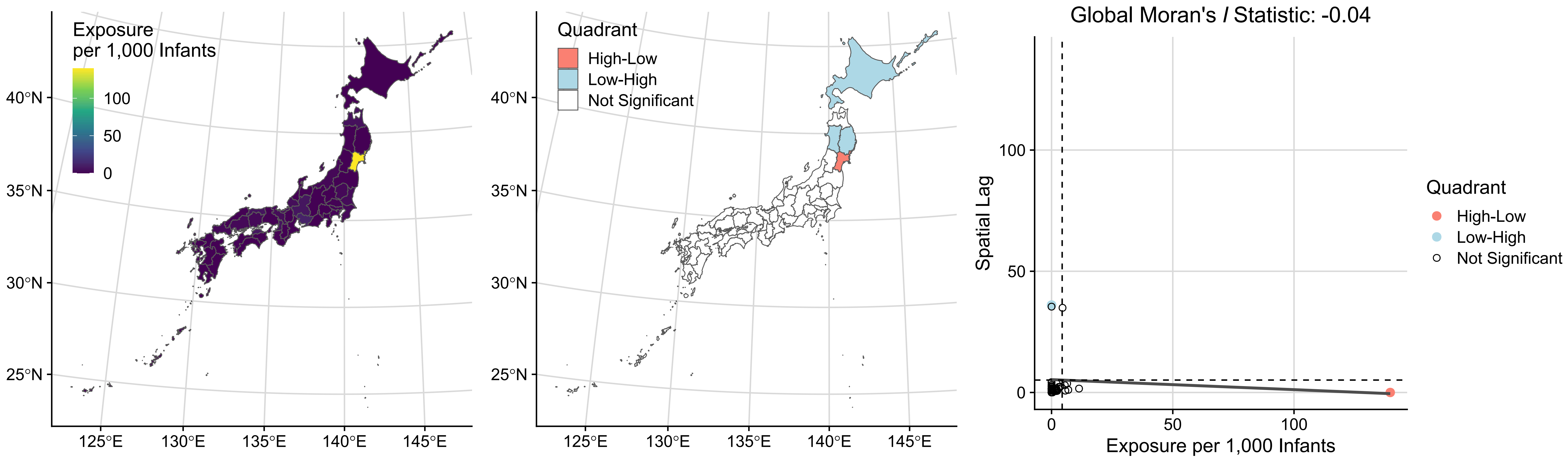

J01XX09. Daptomycin

Early Neonatal Exposure among Very Preterm and Very Low Birth Weight Infants (Days 0–6)

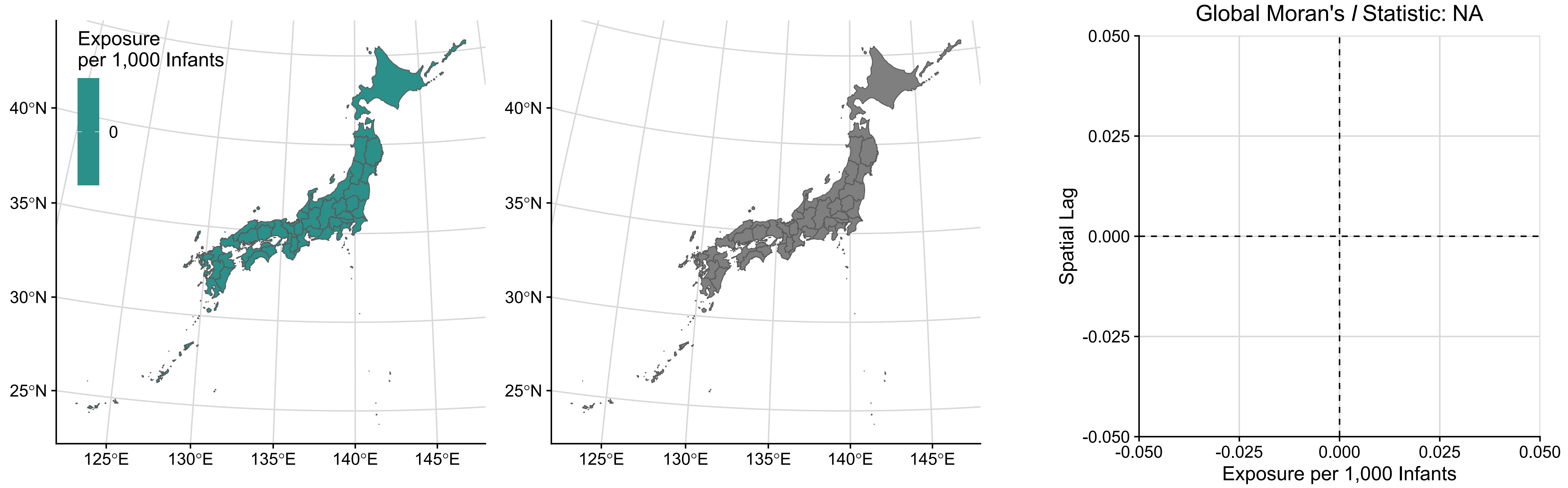

Neonatal Exposure among Very Preterm and Very Low Birth Weight Infants (Days 0–27)

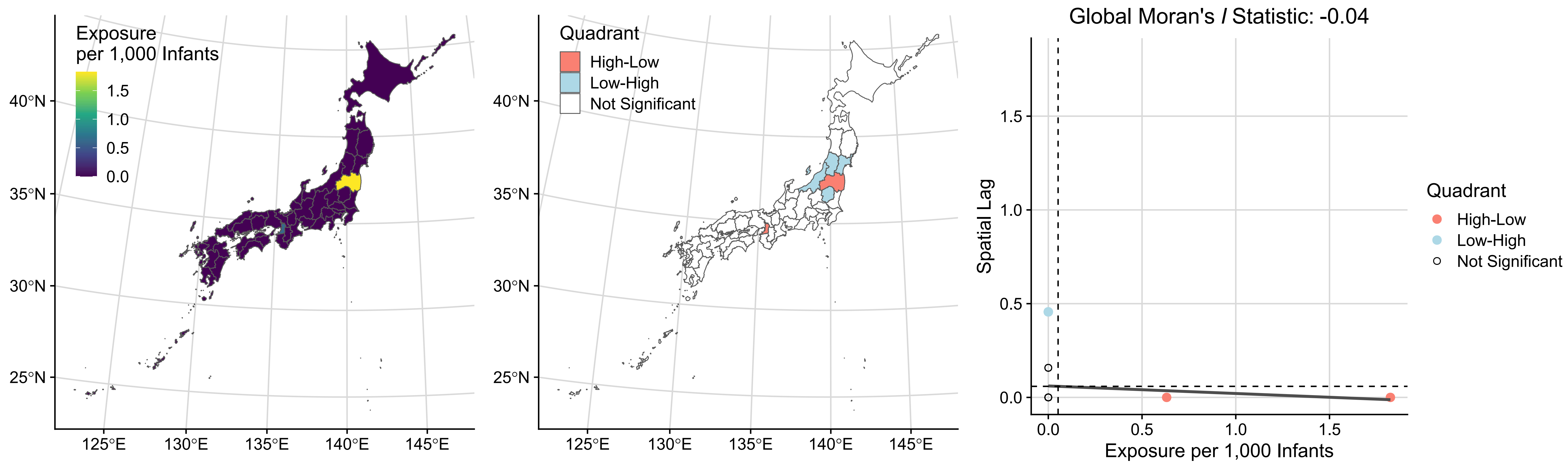

J02AA01. Amphotericin B

Early Neonatal Exposure among Very Preterm and Very Low Birth Weight Infants (Days 0–6)

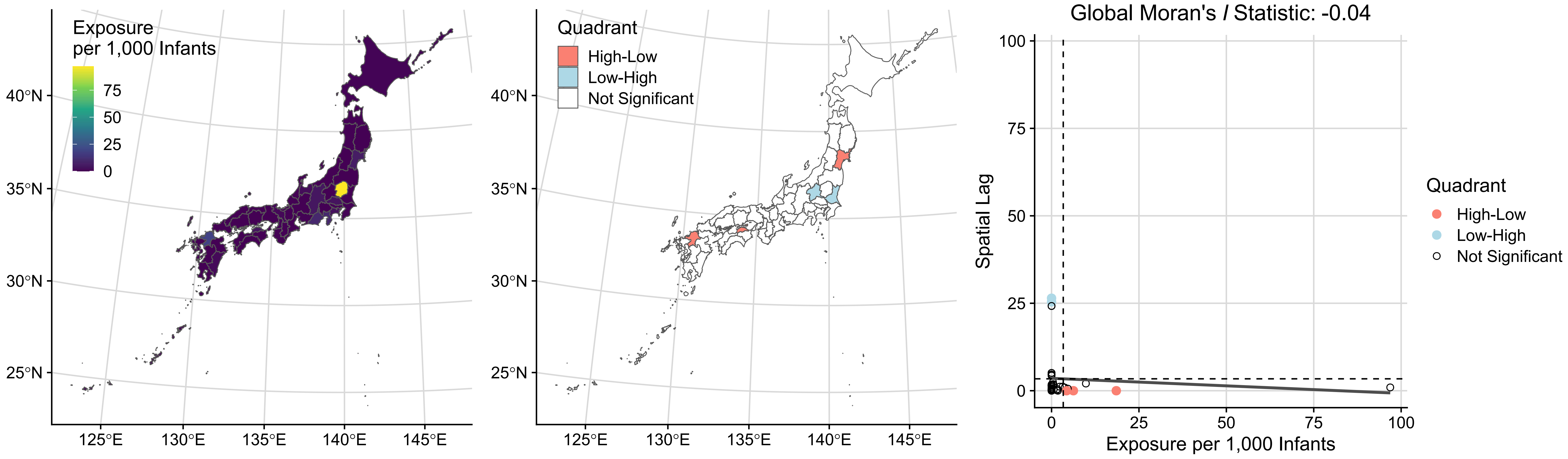

Neonatal Exposure among Very Preterm and Very Low Birth Weight Infants (Days 0–27)

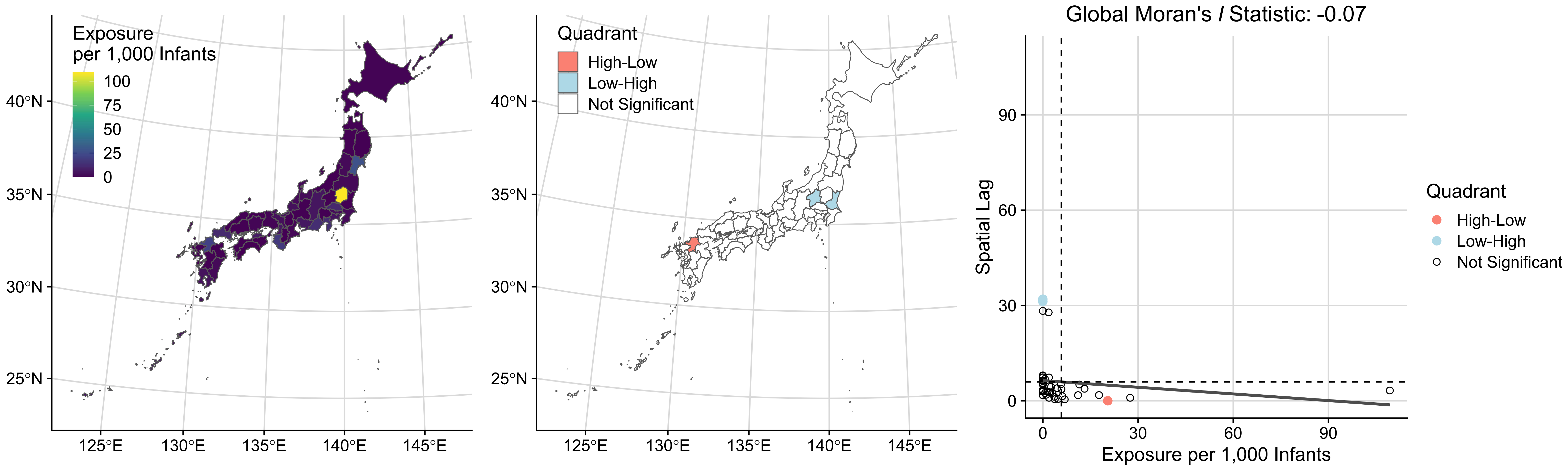

J02AB01. Miconazole

Early Neonatal Exposure among Very Preterm and Very Low Birth Weight Infants (Days 0–6)

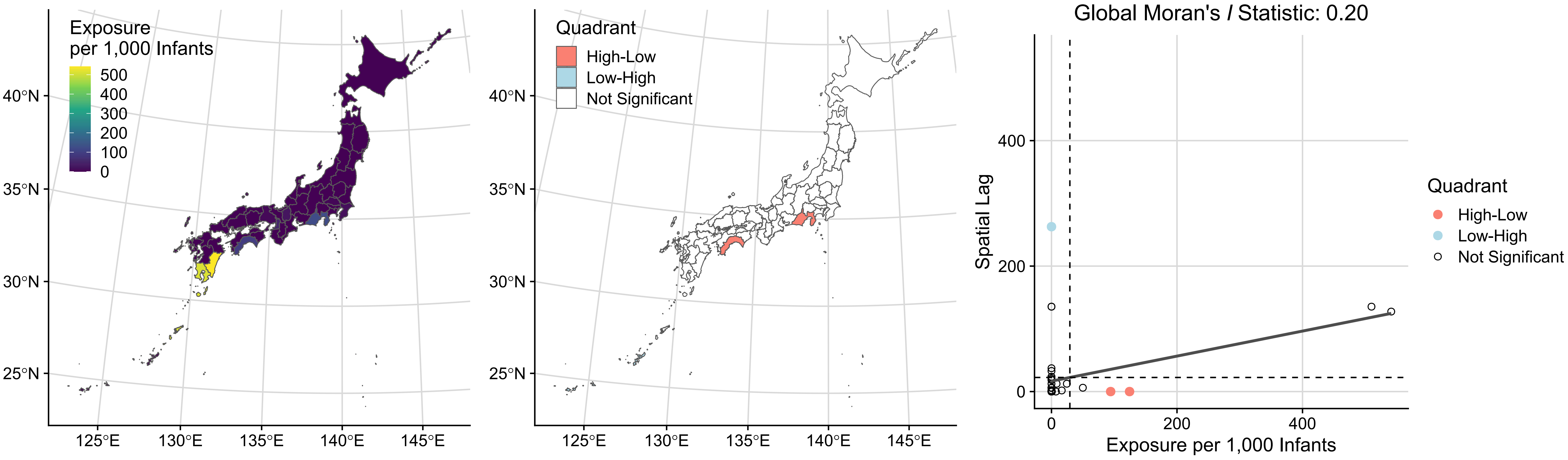

Neonatal Exposure among Very Preterm and Very Low Birth Weight Infants (Days 0–27)

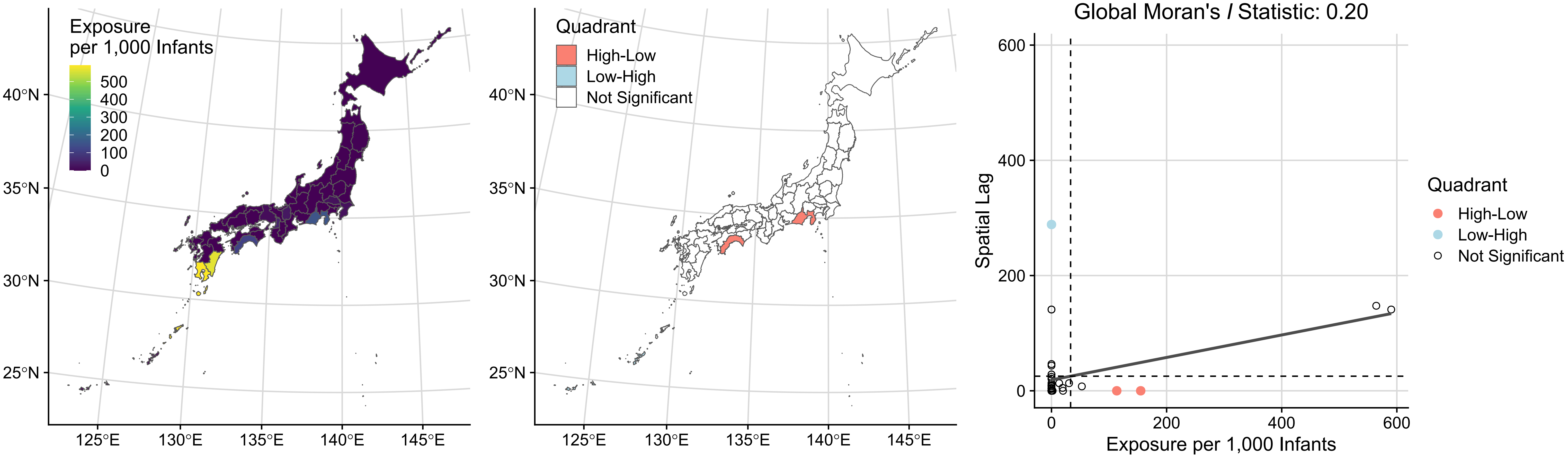

J02AC01. Fluconazole

Early Neonatal Exposure among Very Preterm and Very Low Birth Weight Infants (Days 0–6)

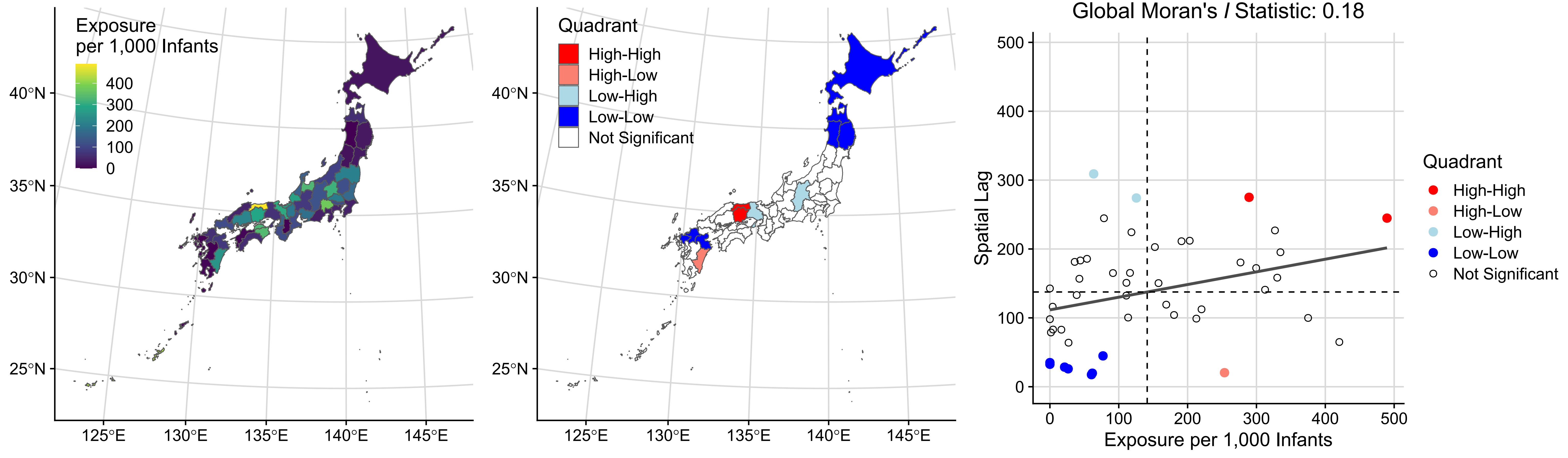

Neonatal Exposure among Very Preterm and Very Low Birth Weight Infants (Days 0–27)

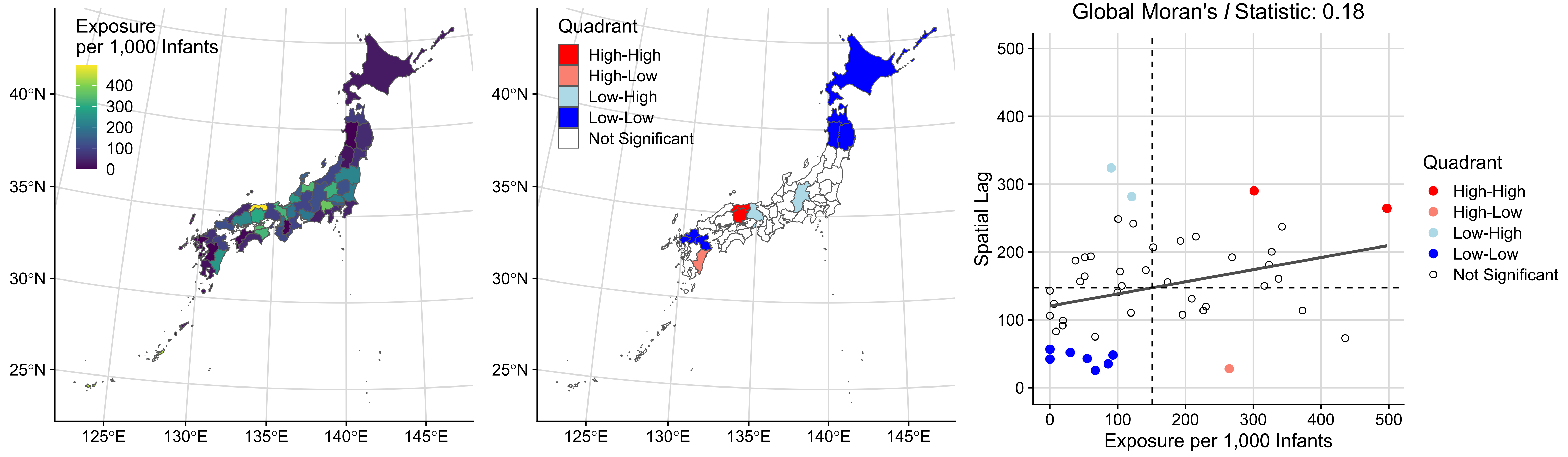

J02AC03. Voriconazole

Early Neonatal Exposure among Very Preterm and Very Low Birth Weight Infants (Days 0–6)

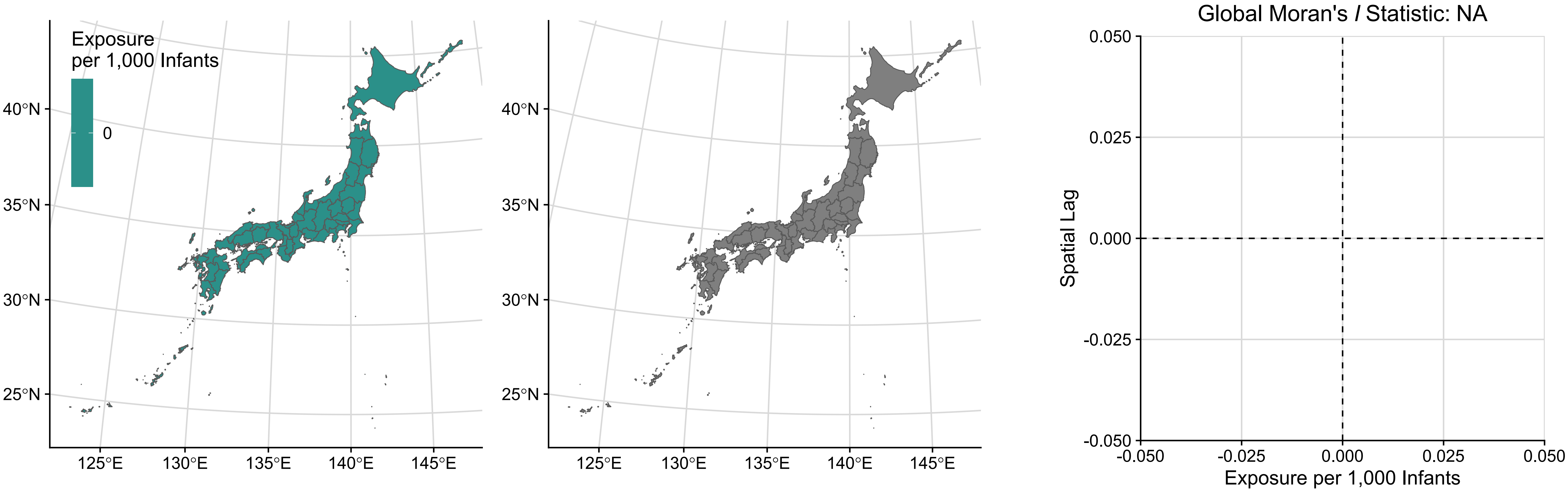

Neonatal Exposure among Very Preterm and Very Low Birth Weight Infants (Days 0–27)

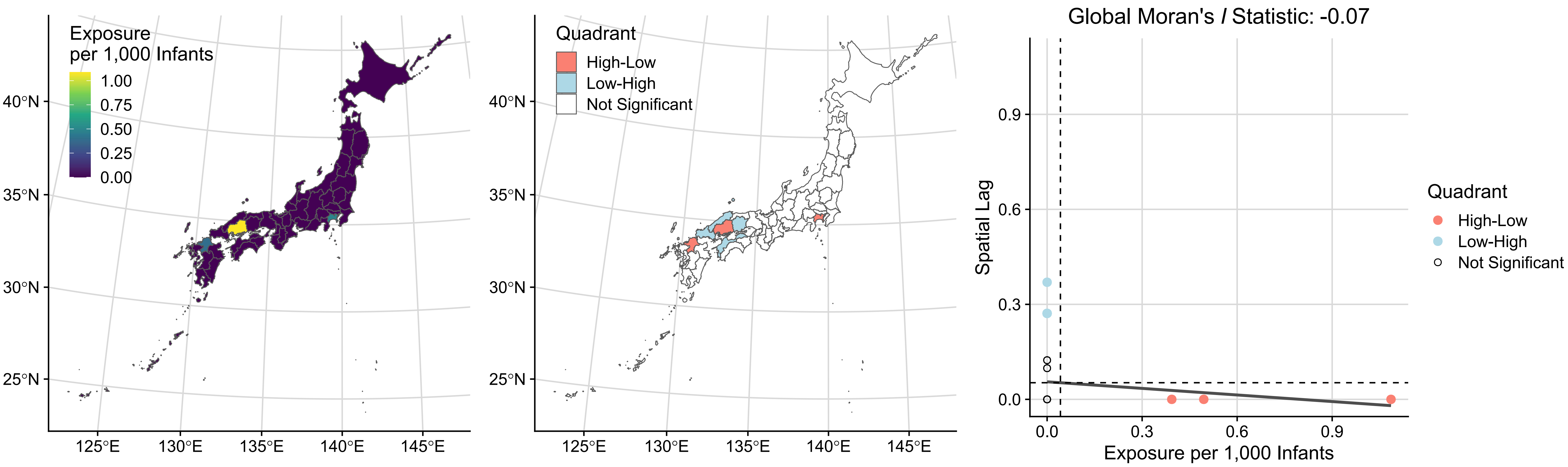

J02AX04. Caspofungin

Early Neonatal Exposure among Very Preterm and Very Low Birth Weight Infants (Days 0–6)

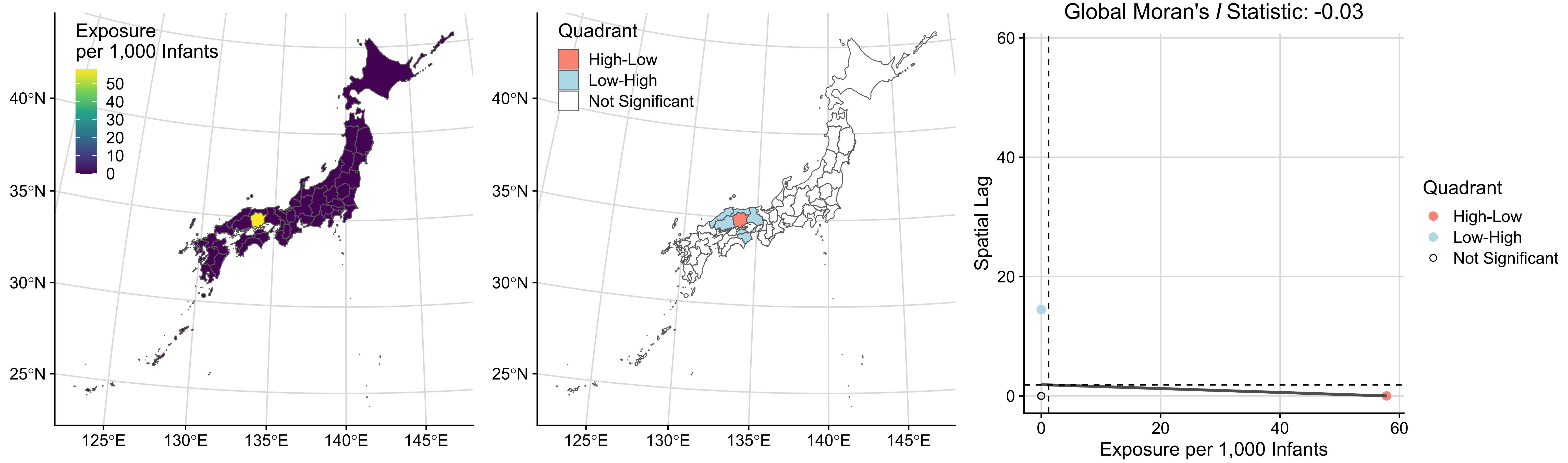

Neonatal Exposure among Very Preterm and Very Low Birth Weight Infants (Days 0–27)

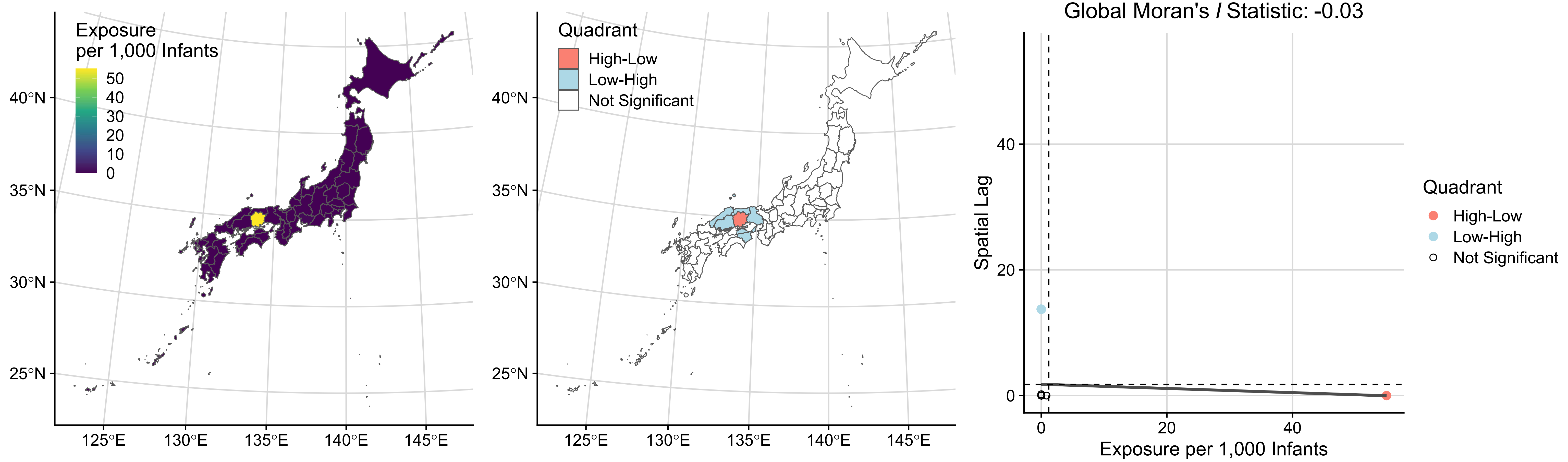

J02AX05. Micafungin

Early Neonatal Exposure among Very Preterm and Very Low Birth Weight Infants (Days 0–6)

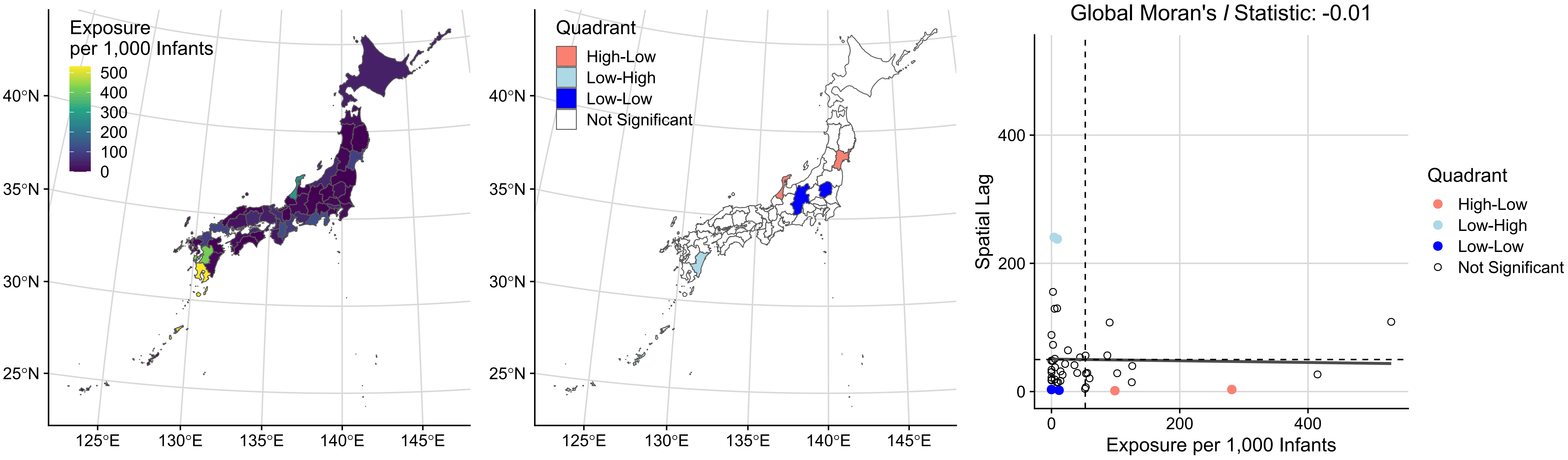

Neonatal Exposure among Very Preterm and Very Low Birth Weight Infants (Days 0–27)

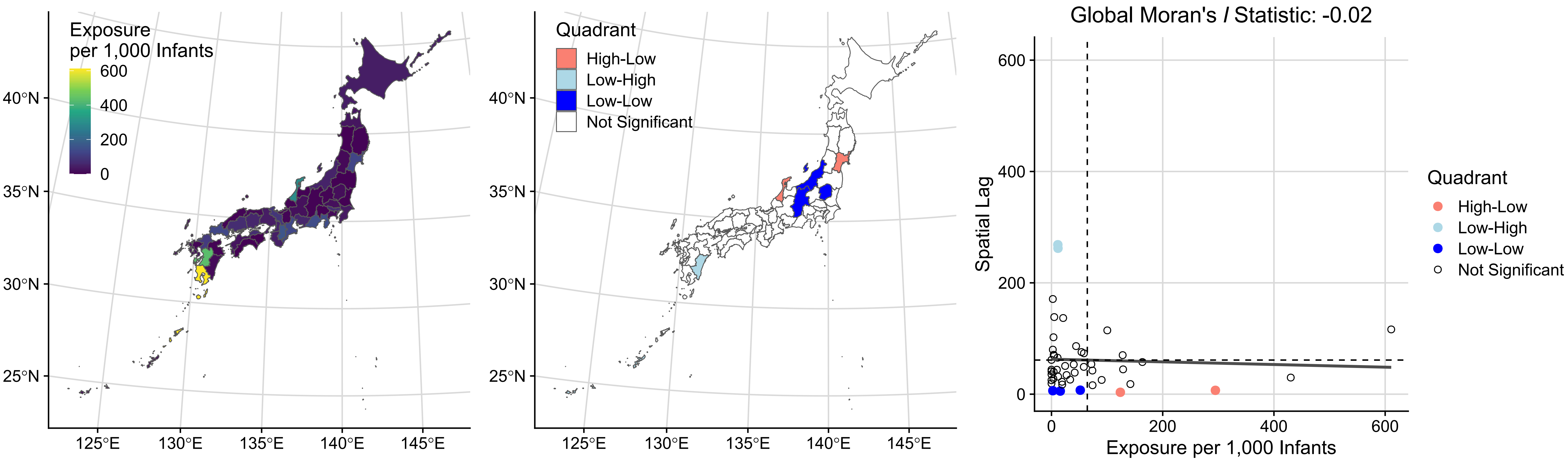

J04AC01. Isoniazid

Early Neonatal Exposure among Very Preterm and Very Low Birth Weight Infants (Days 0–6)

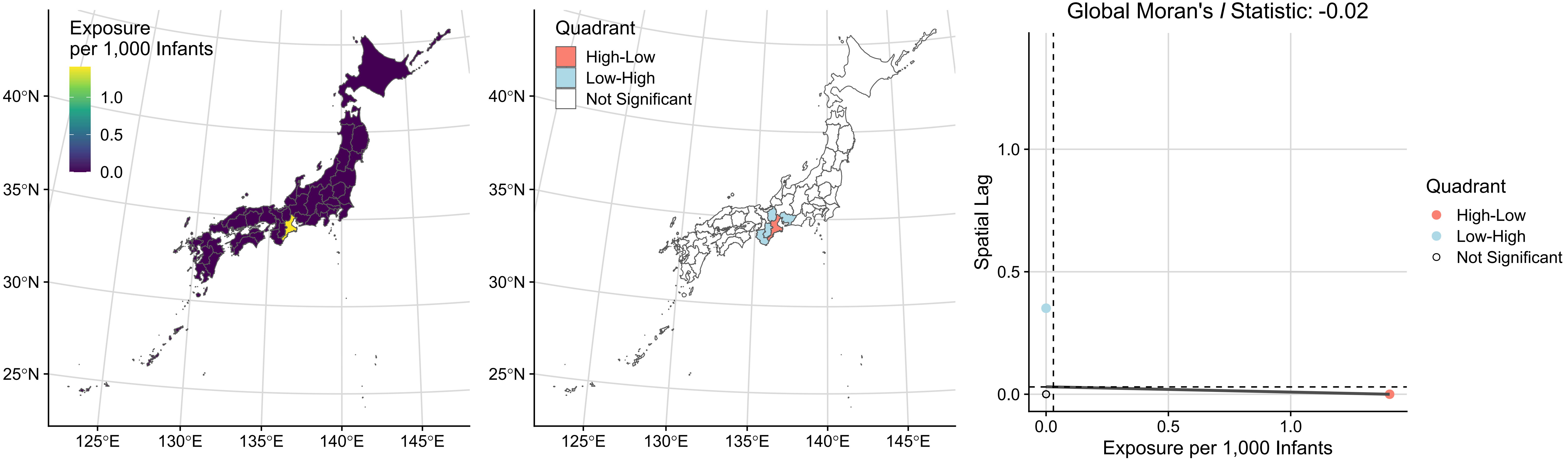

Neonatal Exposure among Very Preterm and Very Low Birth Weight Infants (Days 0–27)

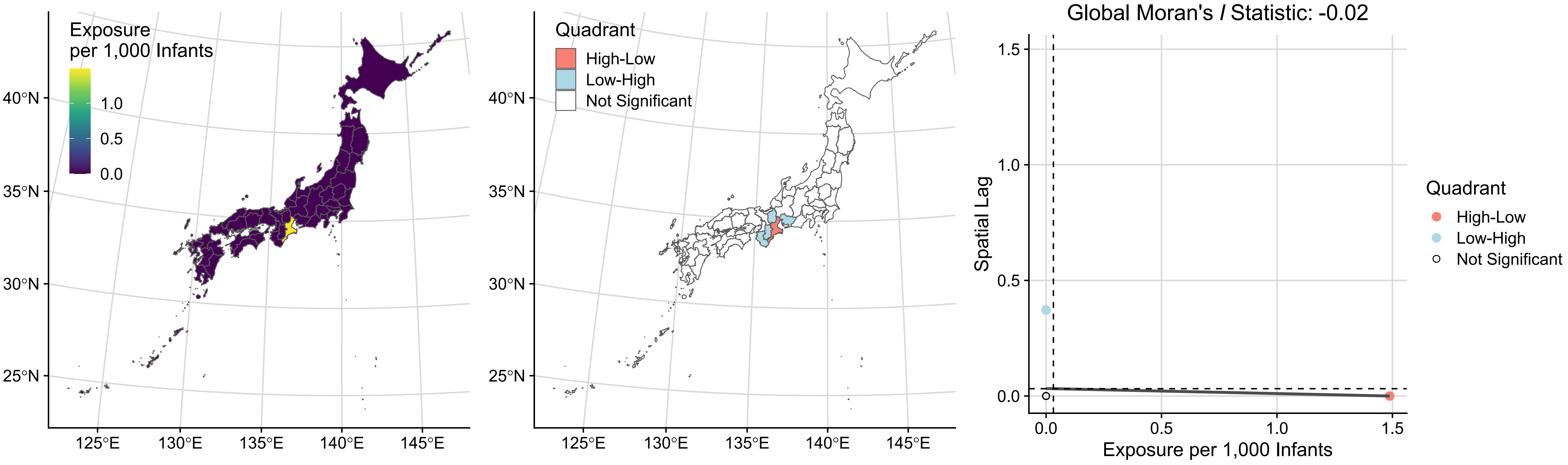

J05AB01. Aciclovir

Early Neonatal Exposure among Very Preterm and Very Low Birth Weight Infants (Days 0–6)

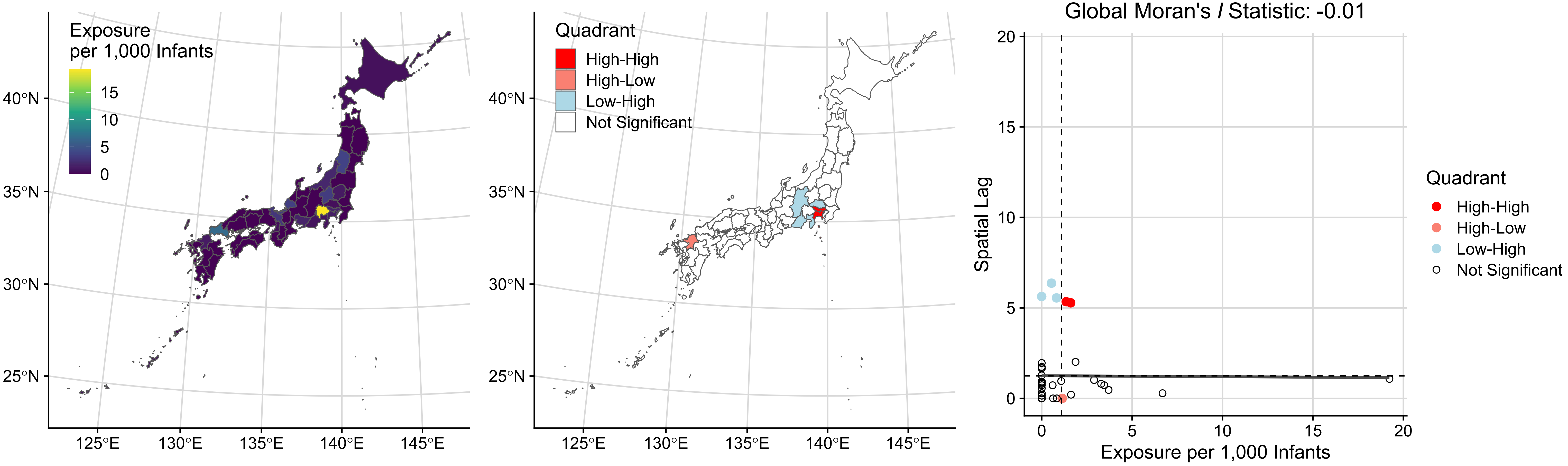

Neonatal Exposure among Very Preterm and Very Low Birth Weight Infants (Days 0–27)

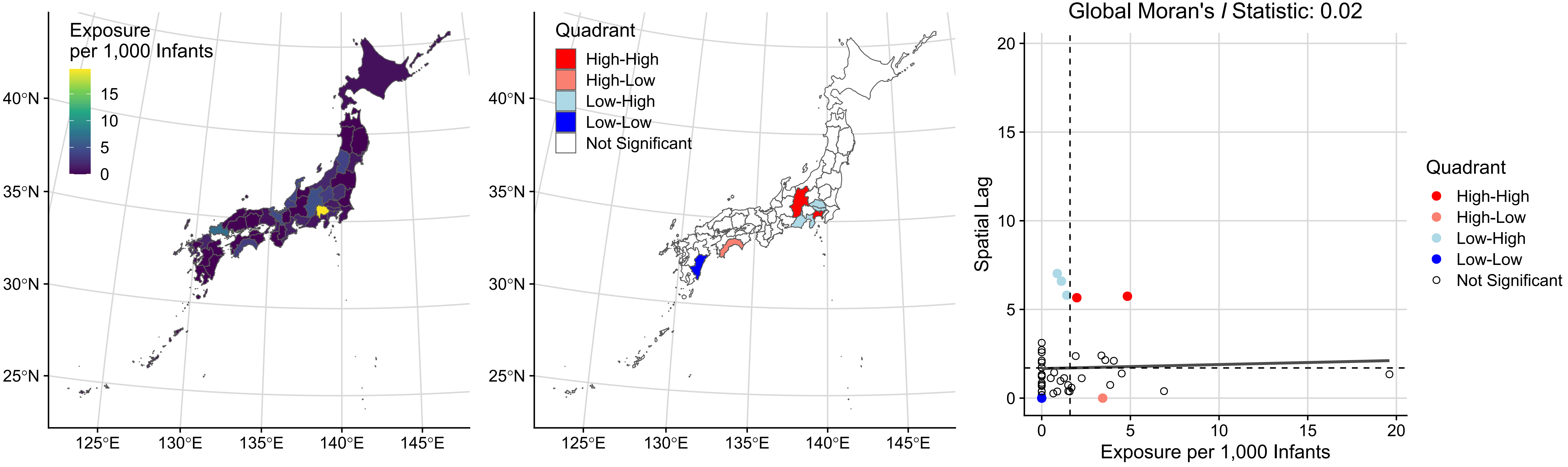

J05AB06. Ganciclovir

Early Neonatal Exposure among Very Preterm and Very Low Birth Weight Infants (Days 0–6)

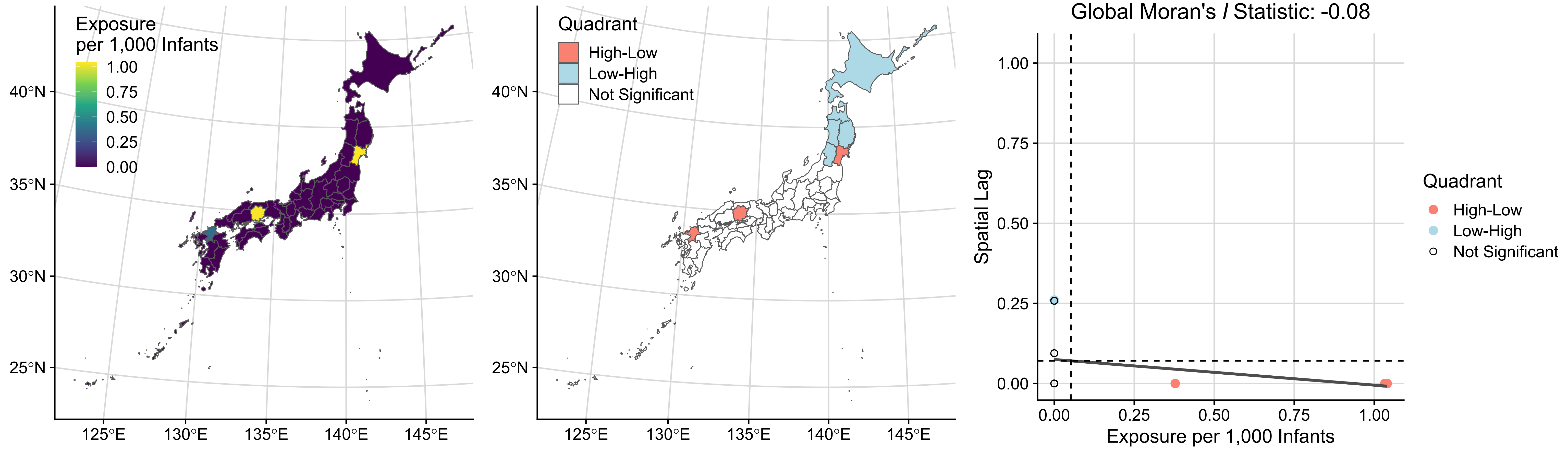

Neonatal Exposure among Very Preterm and Very Low Birth Weight Infants (Days 0–27)

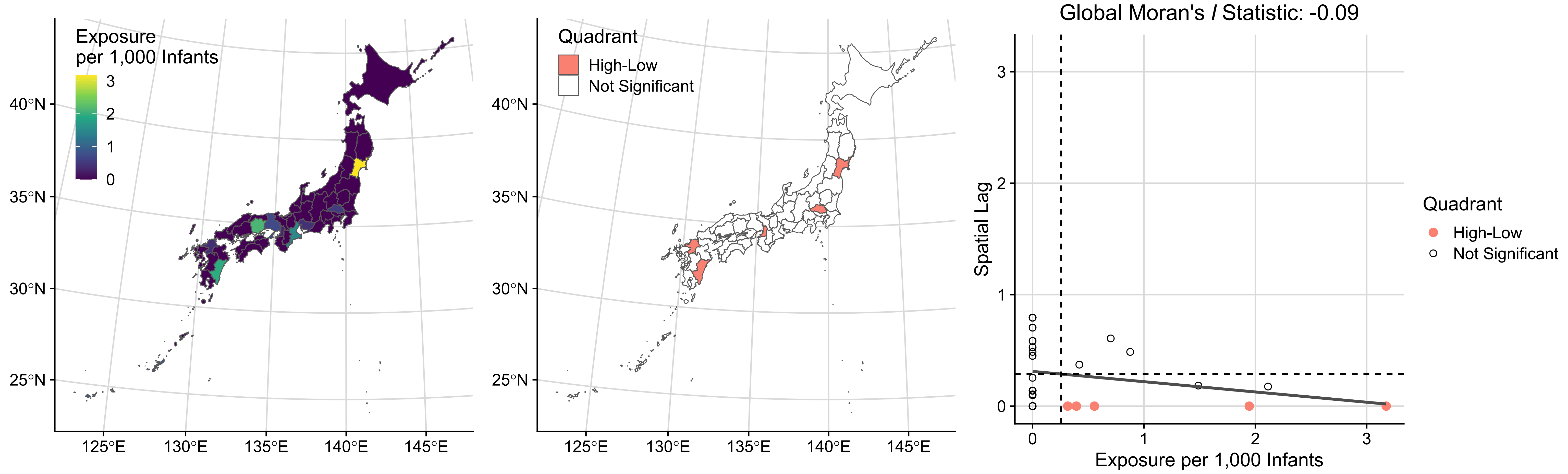

J05AH03. Peramivir

Early Neonatal Exposure among Very Preterm and Very Low Birth Weight Infants (Days 0–6)

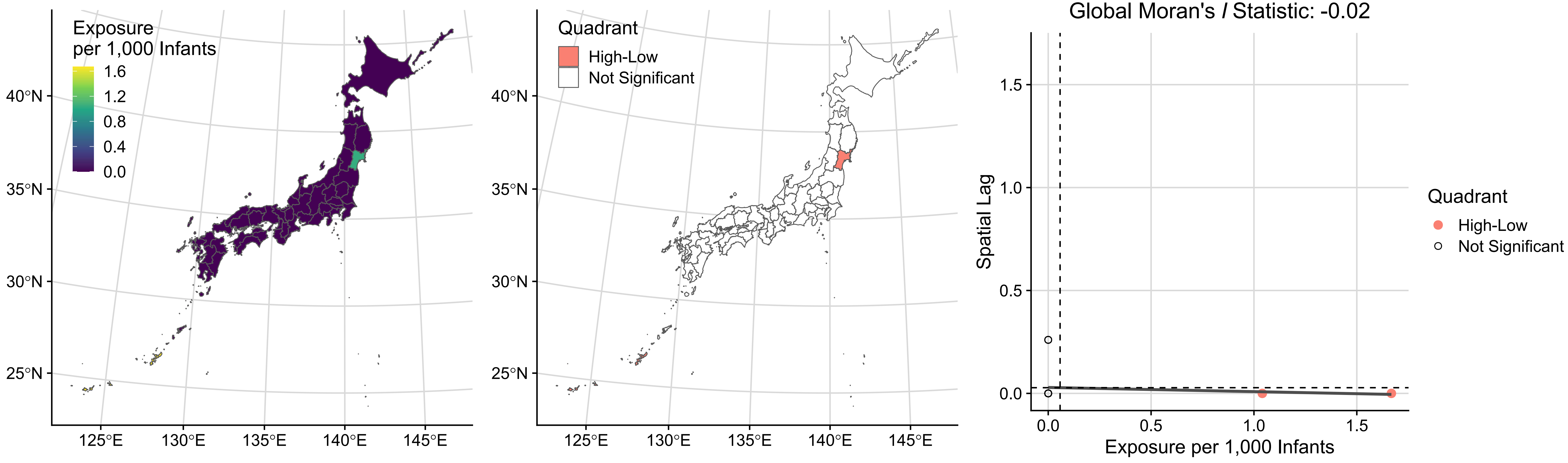

Neonatal Exposure among Very Preterm and Very Low Birth Weight Infants (Days 0–27)

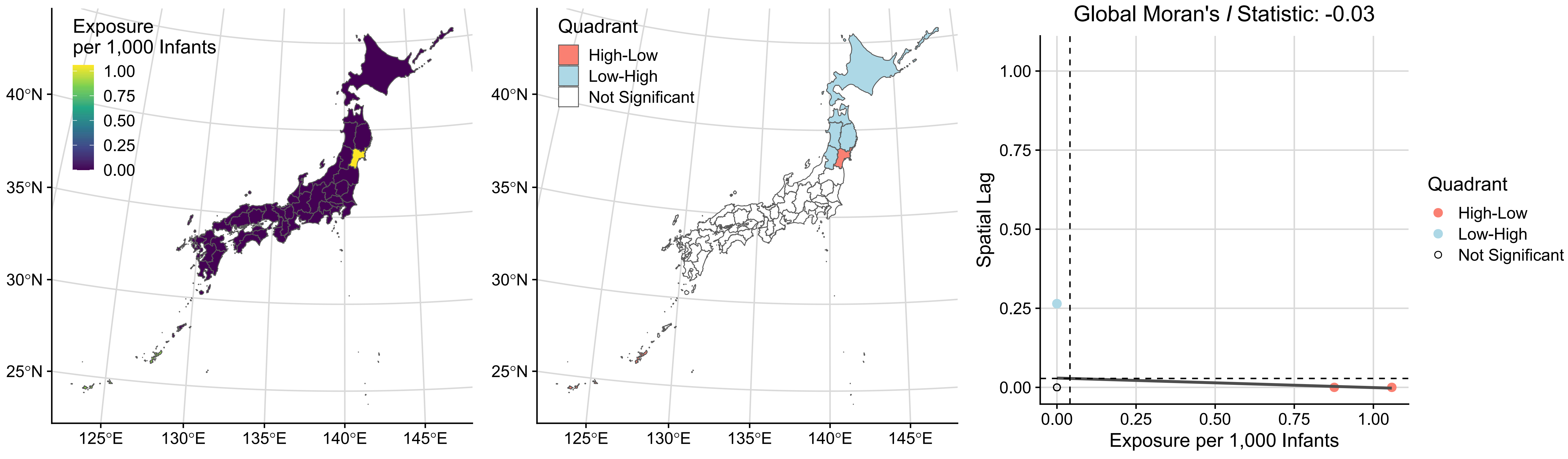

Supplement: S1 Fig — (PDF) [file pone.0295528.s002.pdf]
