# Supplementary material for: Geographical distribution of antimicrobial exposure among very preterm and very low birth weight infants: A nationwide database study in Japan
Source: PLoS One. 2024 Jan 25;19(1):e0295528. doi: 10.1371/journal.pone.0295528 (PMC10810499; doi:10.1371/journal.pone.0295528)

J01AA. Tetracyclines

Early Neonatal Exposure among Very Preterm and Very Low Birth Weight Infants (Days 0–6)

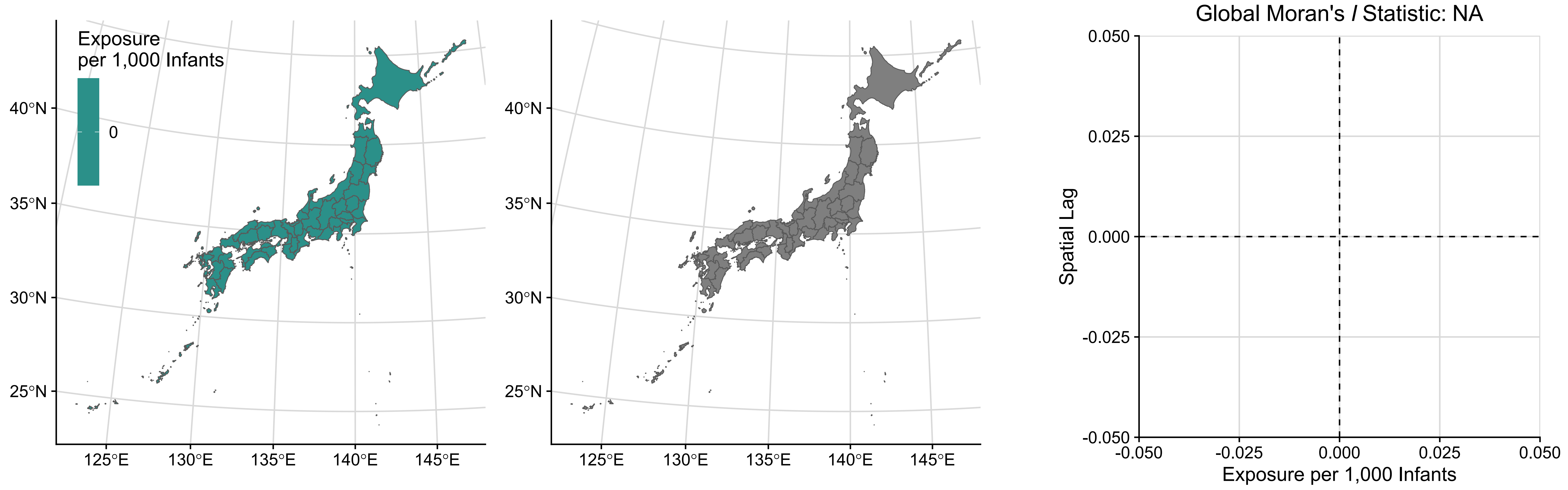

Neonatal Exposure among Very Preterm and Very Low Birth Weight Infants (Days 0–27)

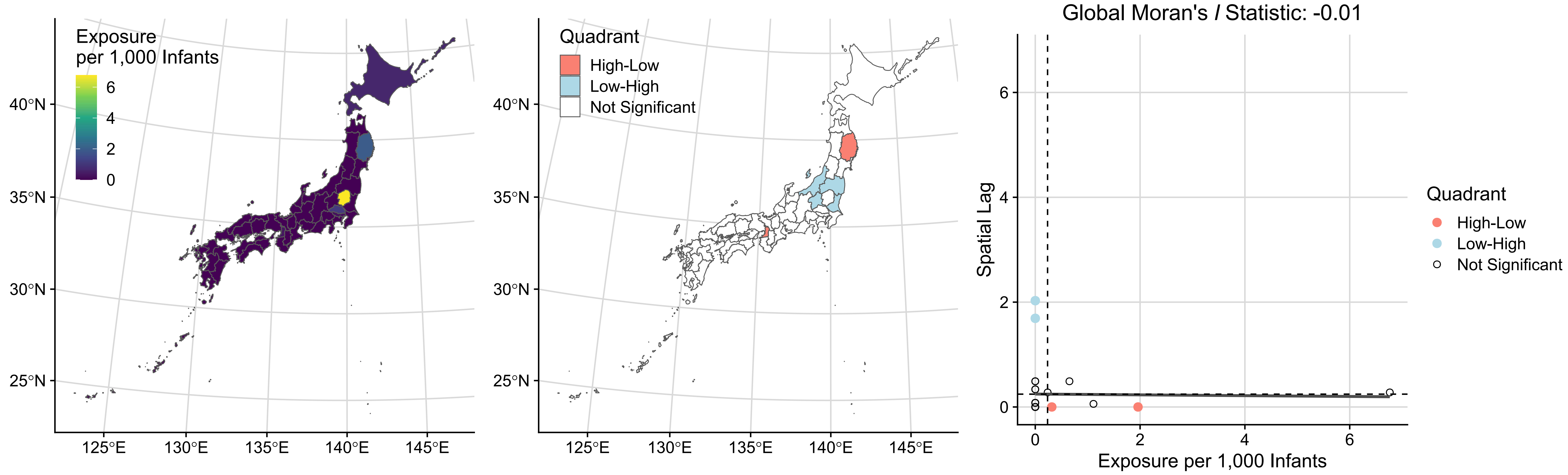

J01BA. Amphenicols

Early Neonatal Exposure among Very Preterm and Very Low Birth Weight Infants (Days 0–6)

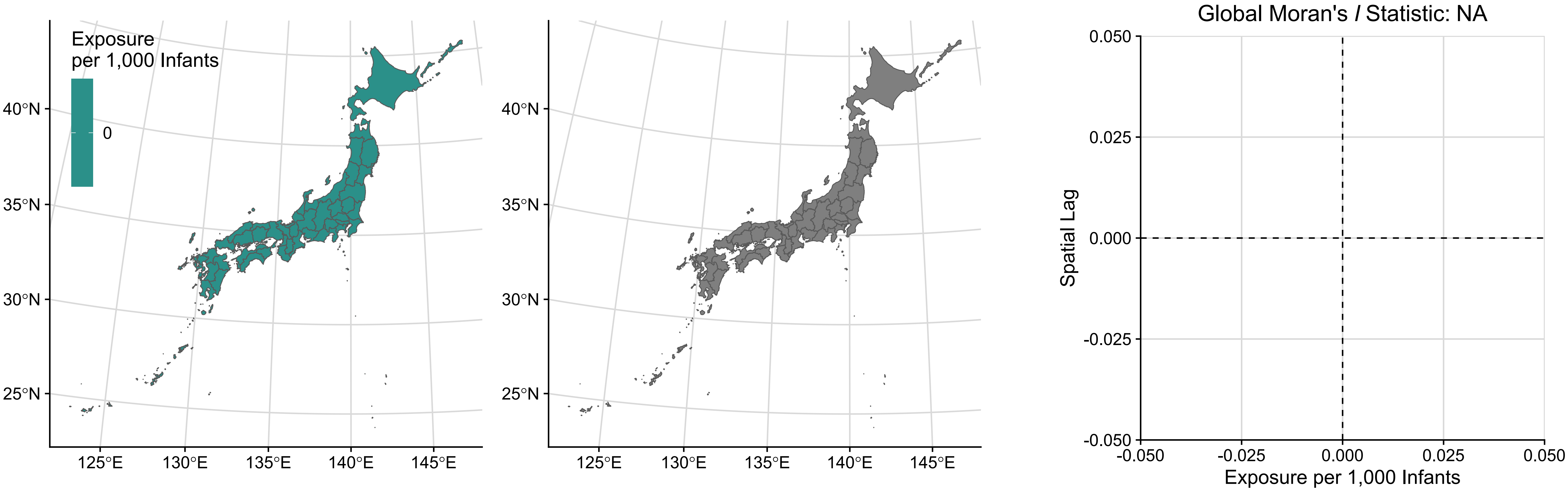

Neonatal Exposure among Very Preterm and Very Low Birth Weight Infants (Days 0–27)

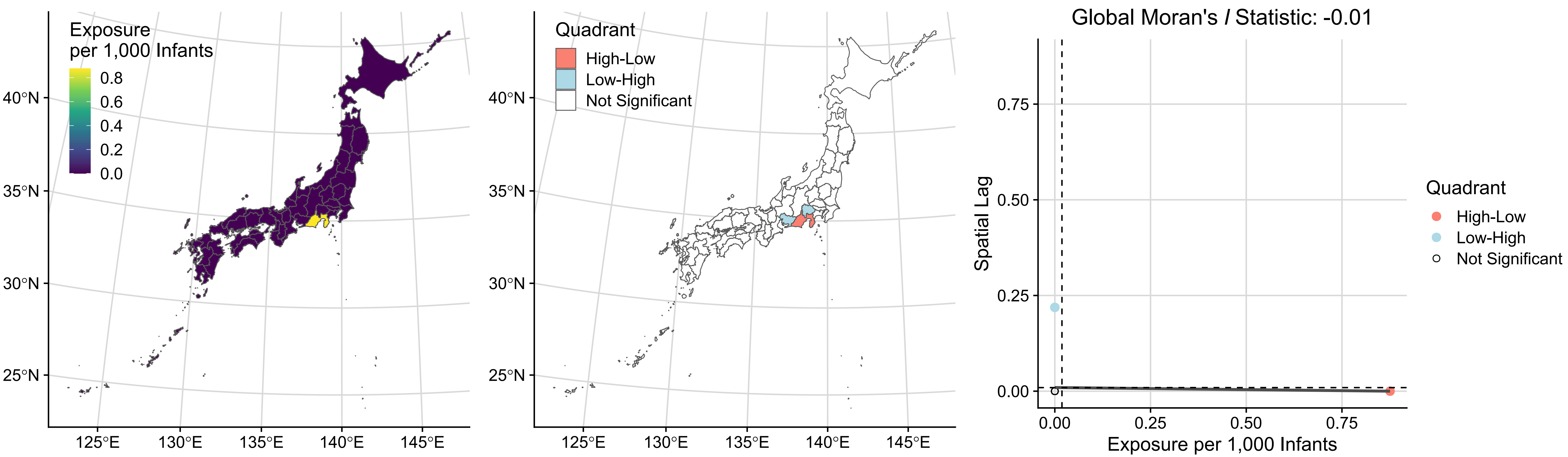

J01CA. Penicillins with Extended Spectrum

Early Neonatal Exposure among Very Preterm and Very Low Birth Weight Infants (Days 0–6)

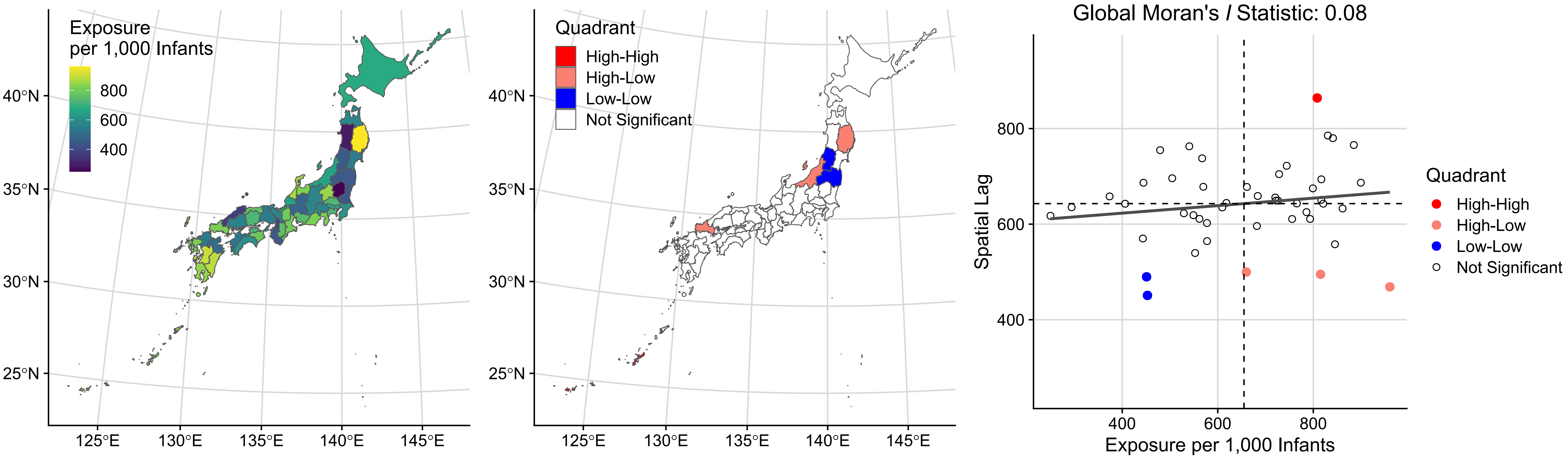

Neonatal Exposure among Very Preterm and Very Low Birth Weight Infants (Days 0–27)

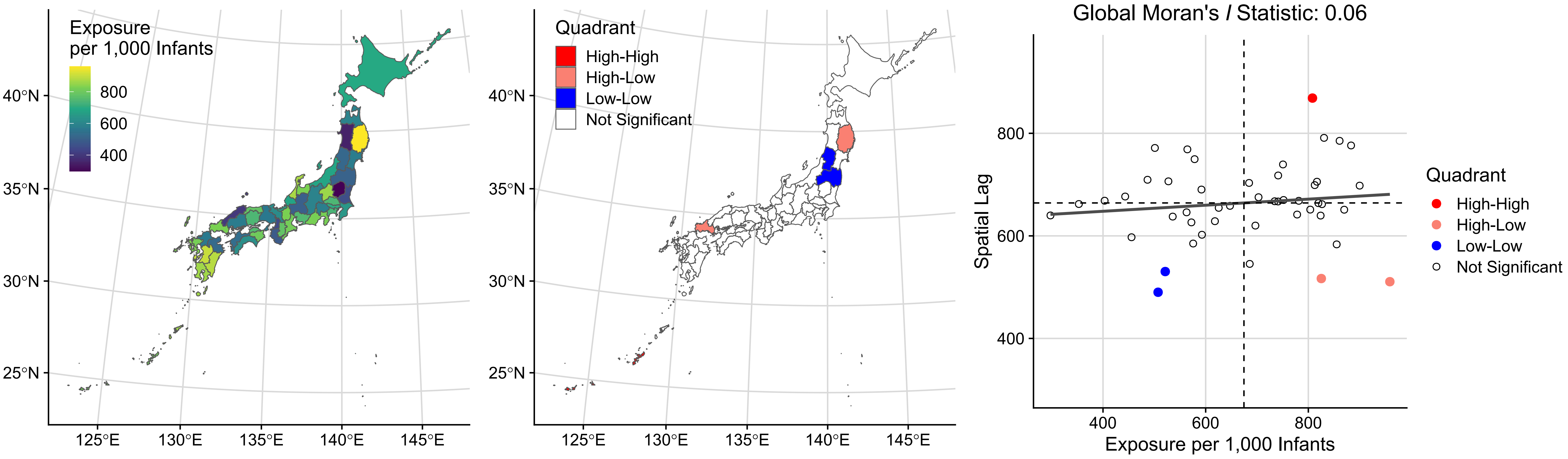

J01CE. Beta-Lactamase Sensitive Penicillins

Early Neonatal Exposure among Very Preterm and Very Low Birth Weight Infants (Days 0–6)

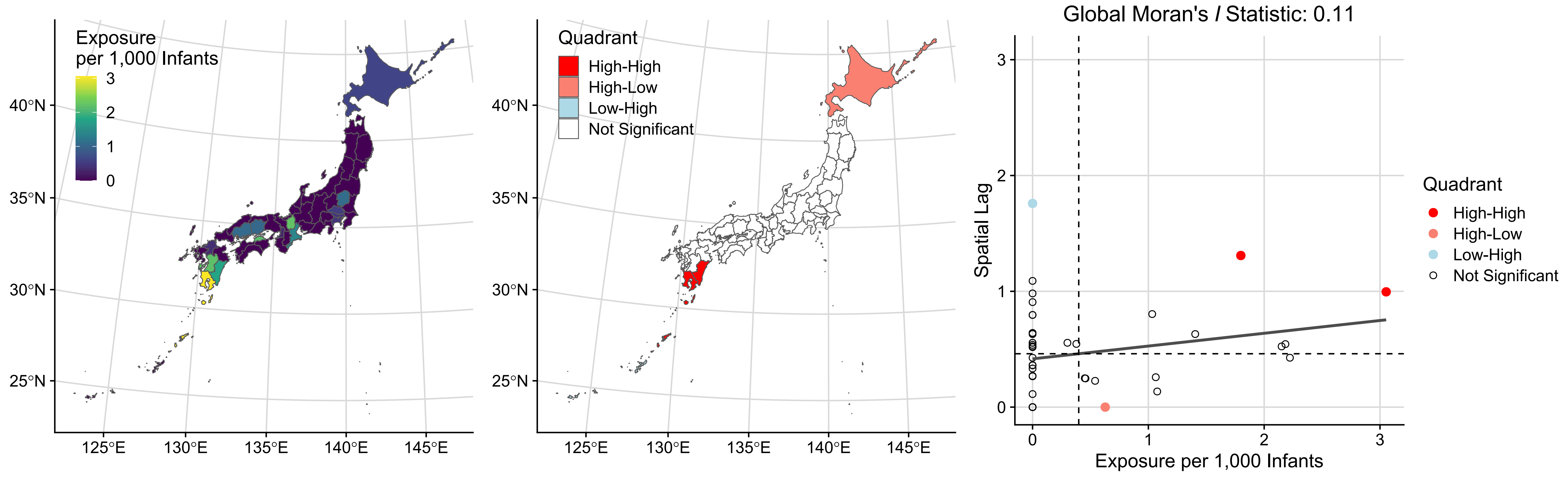

Neonatal Exposure among Very Preterm and Very Low Birth Weight Infants (Days 0–27)

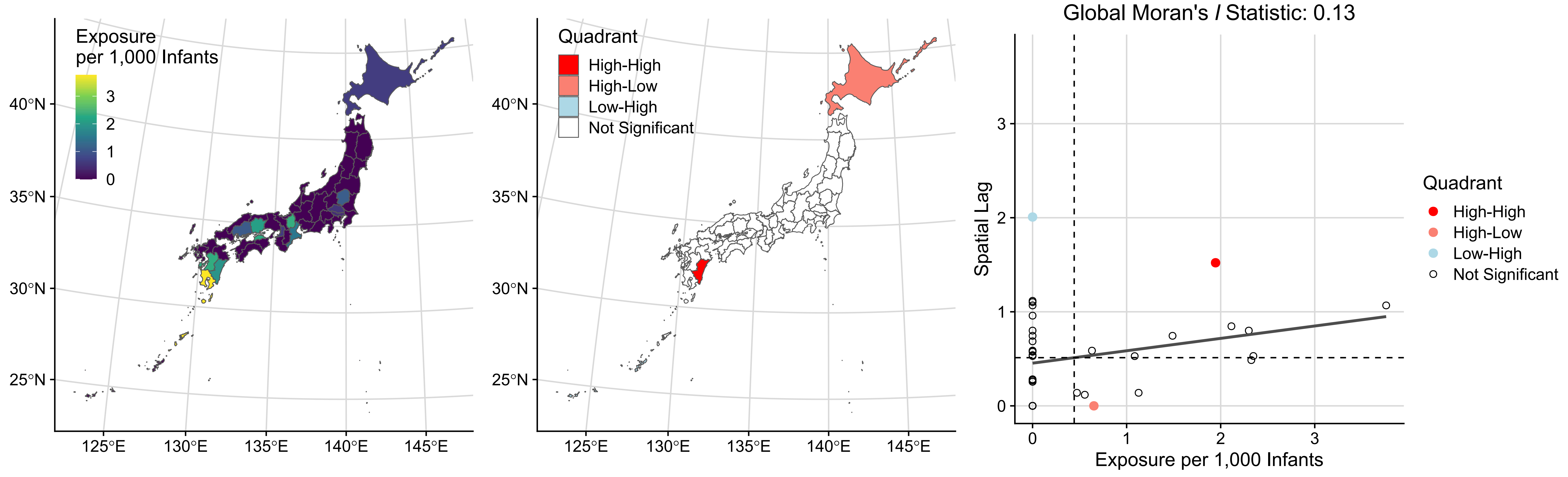

J01CR. Combinations of Penicillins, Including Beta-Lactamase Inhibitors

Early Neonatal Exposure among Very Preterm and Very Low Birth Weight Infants (Days 0–6)

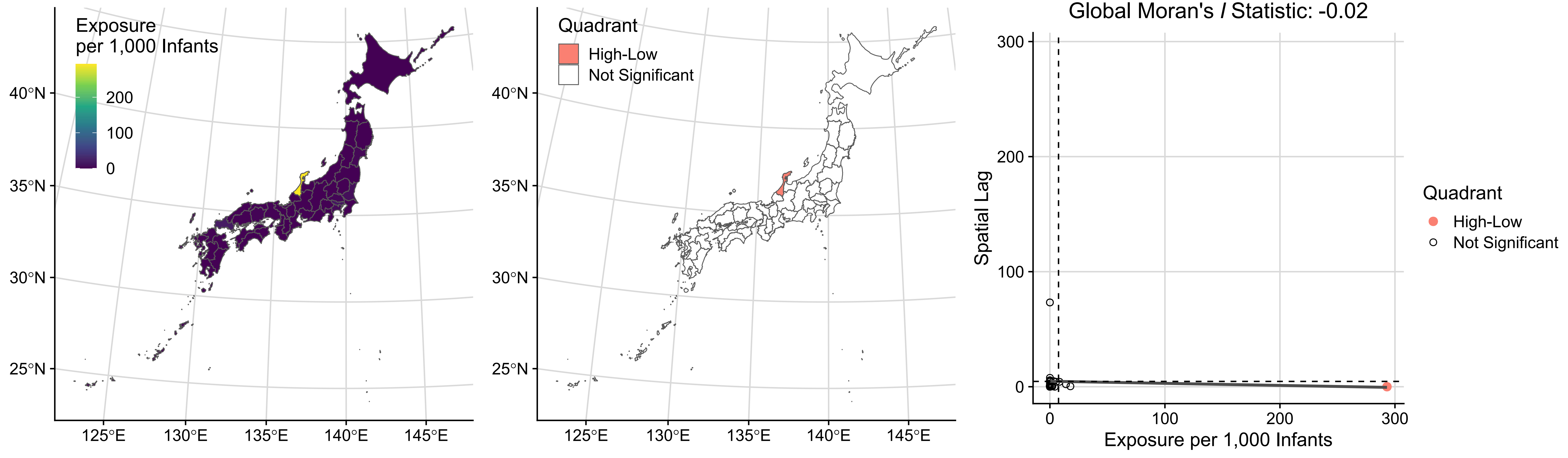

Neonatal Exposure among Very Preterm and Very Low Birth Weight Infants (Days 0–27)

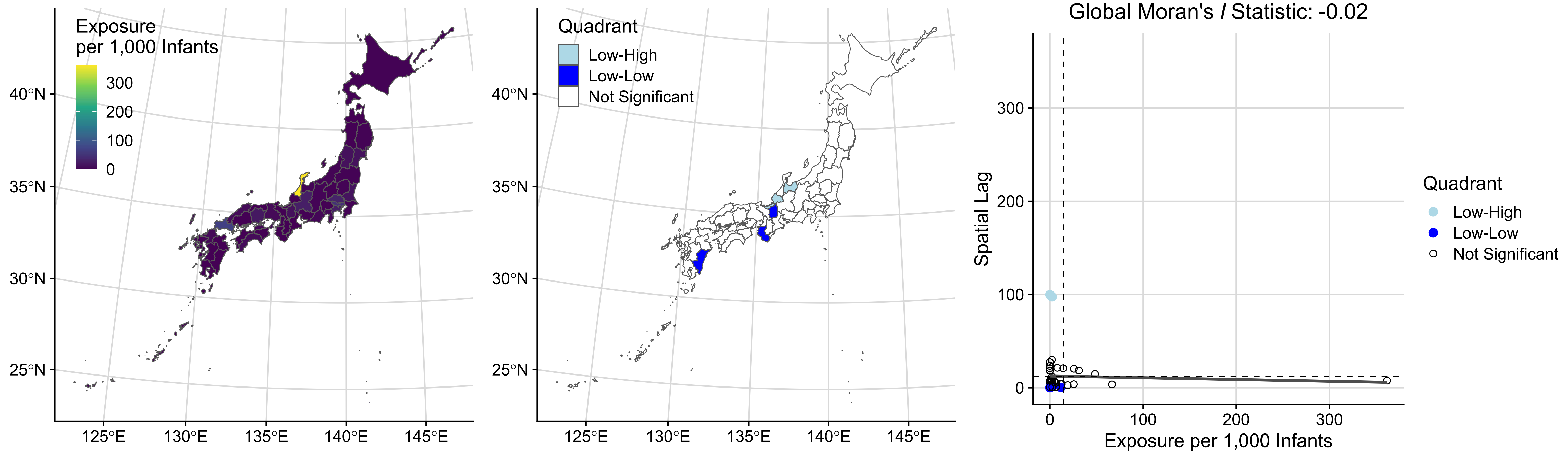

J01DB. First-Generation Cephalosporins

Early Neonatal Exposure among Very Preterm and Very Low Birth Weight Infants (Days 0–6)

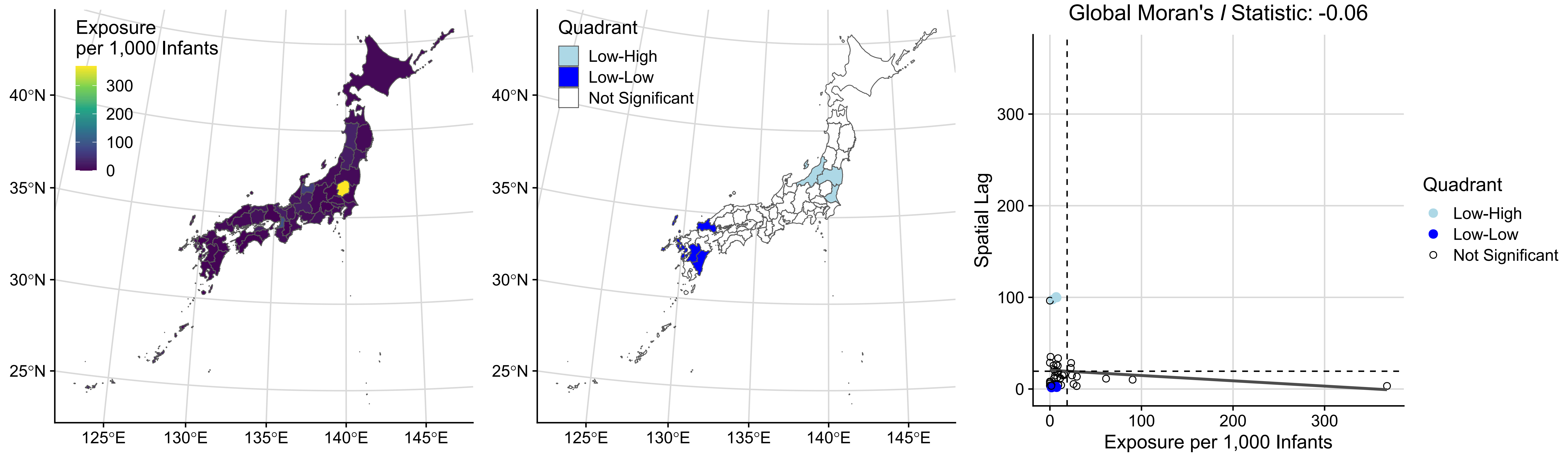

Neonatal Exposure among Very Preterm and Very Low Birth Weight Infants (Days 0–27)

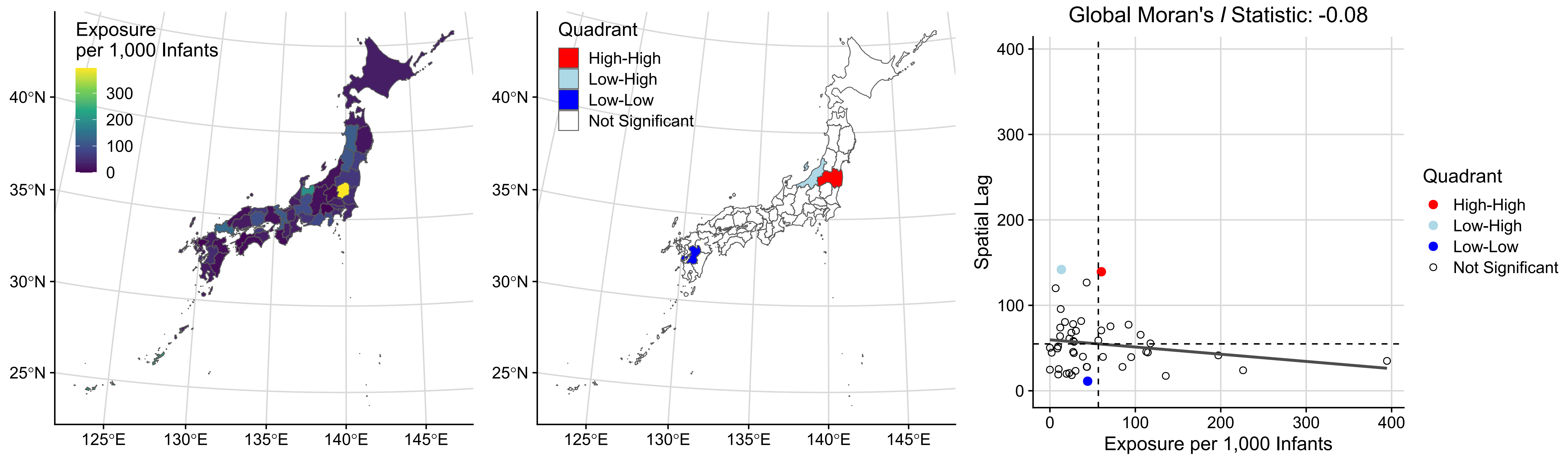

J01DC. Second-Generation Cephalosporins

Early Neonatal Exposure among Very Preterm and Very Low Birth Weight Infants (Days 0–6)

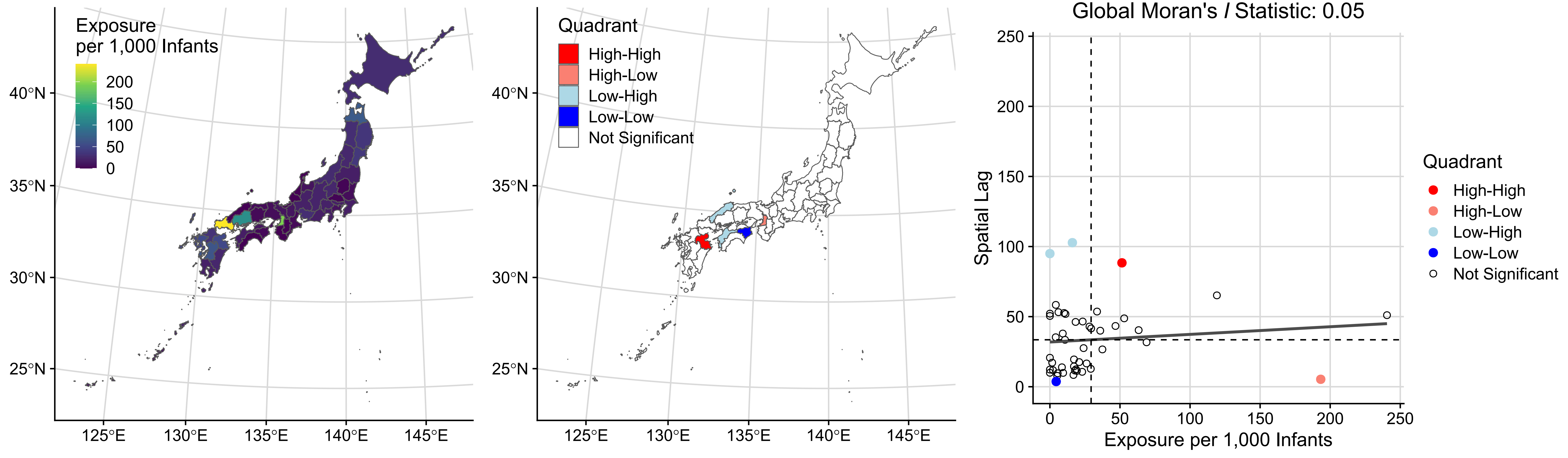

Neonatal Exposure among Very Preterm and Very Low Birth Weight Infants (Days 0–27)

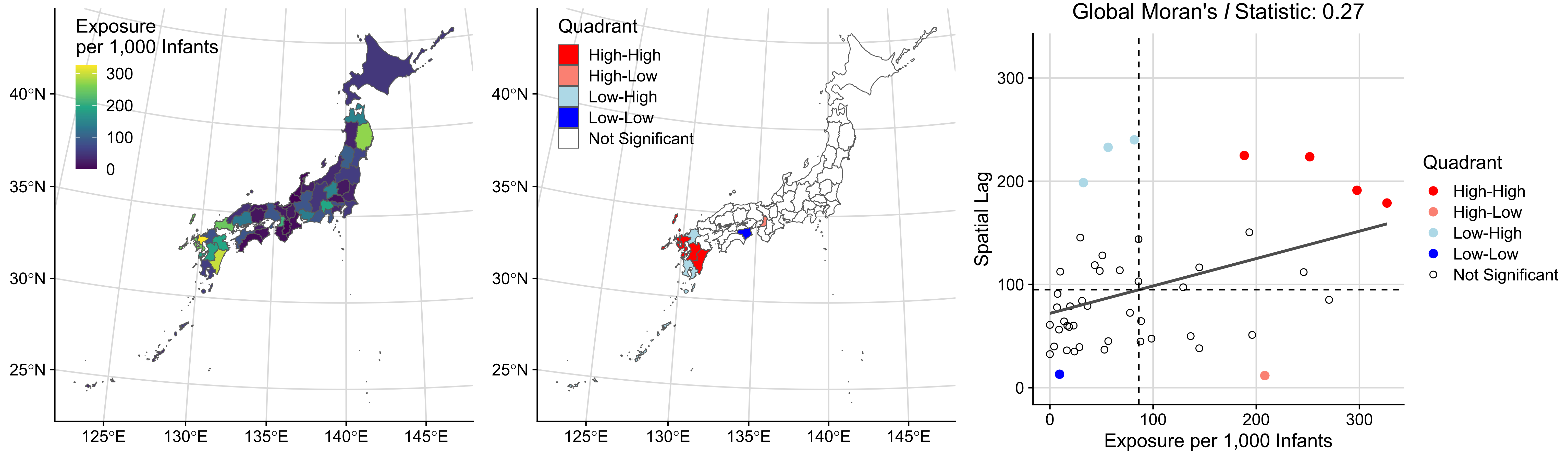

J01DD. Third-Generation Cephalosporins

Early Neonatal Exposure among Very Preterm and Very Low Birth Weight Infants (Days 0–6)

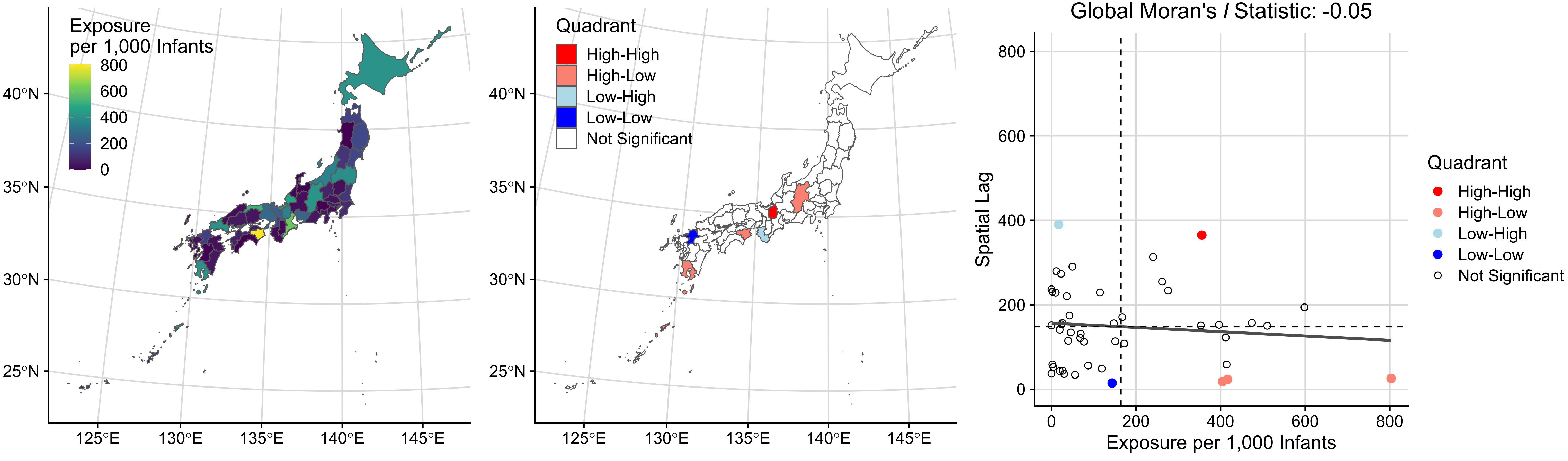

Neonatal Exposure among Very Preterm and Very Low Birth Weight Infants (Days 0–27)

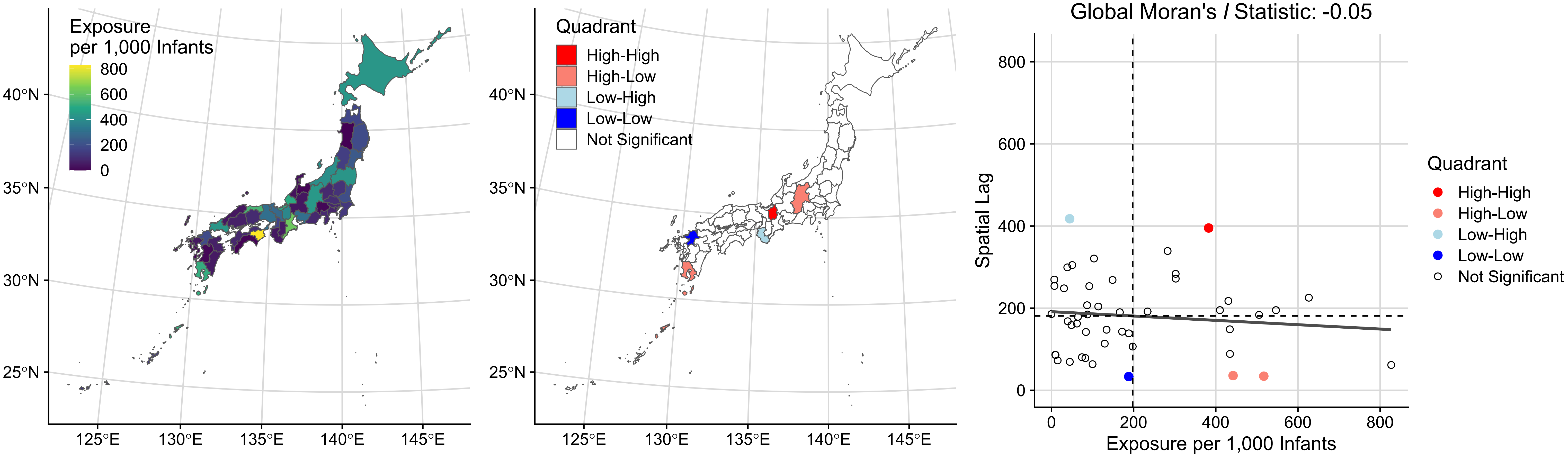

J01DE. Fourth-Generation Cephalosporins

Early Neonatal Exposure among Very Preterm and Very Low Birth Weight Infants (Days 0–6)

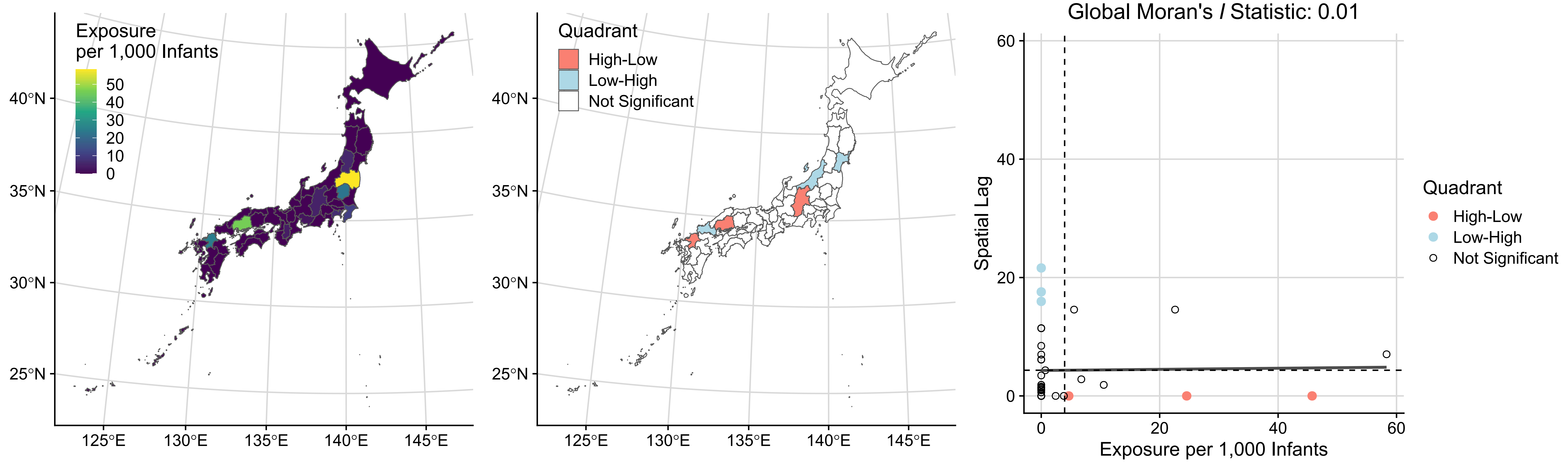

Neonatal Exposure among Very Preterm and Very Low Birth Weight Infants (Days 0–27)

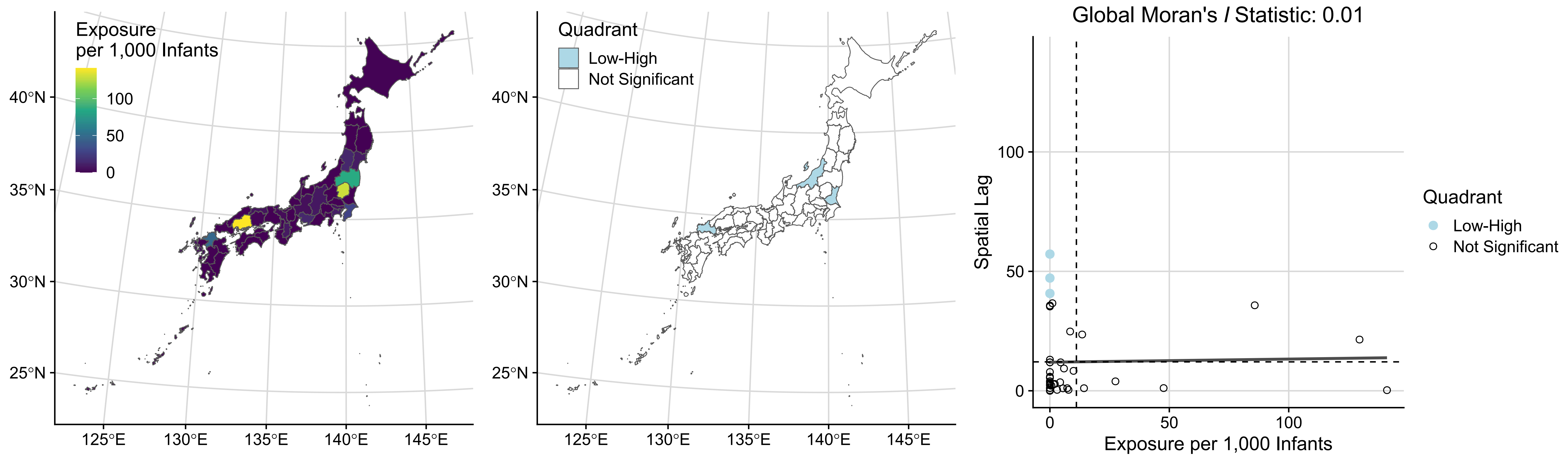

J01DF. Monobactams

Early Neonatal Exposure among Very Preterm and Very Low Birth Weight Infants (Days 0–6)

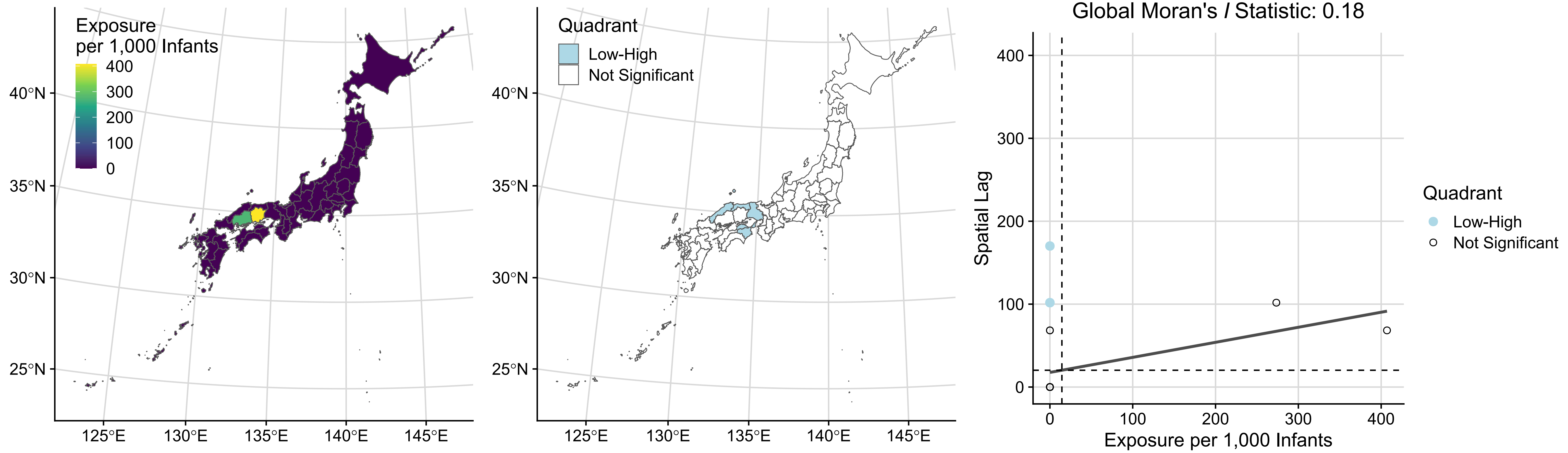

Neonatal Exposure among Very Preterm and Very Low Birth Weight Infants (Days 0–27)

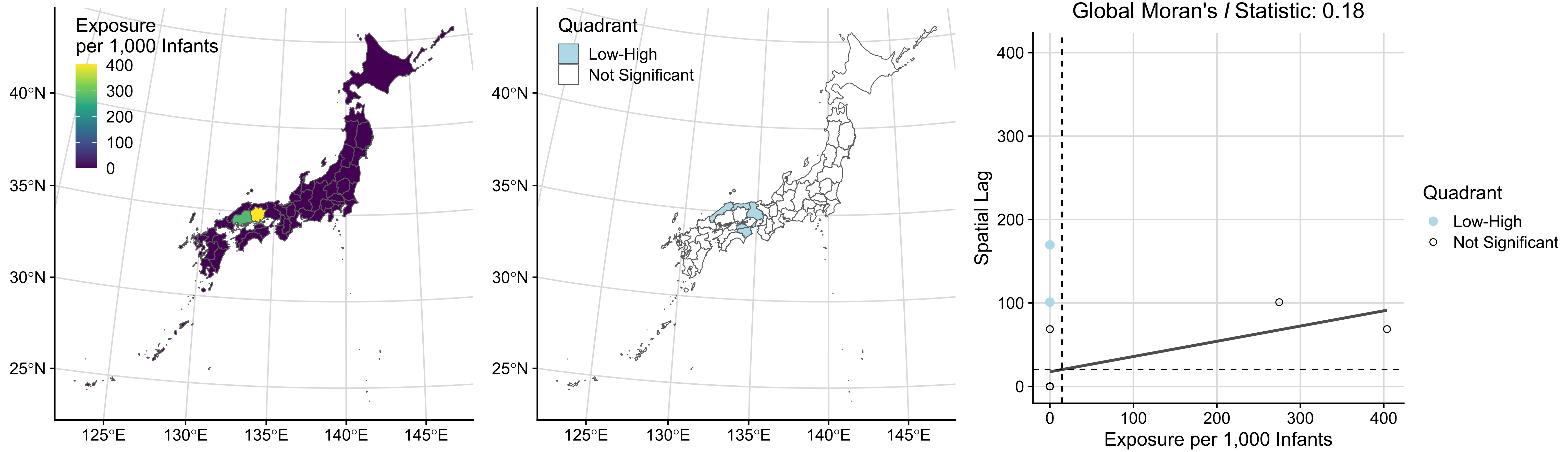

J01DH. Carbapenems

Early Neonatal Exposure among Very Preterm and Very Low Birth Weight Infants (Days 0–6)

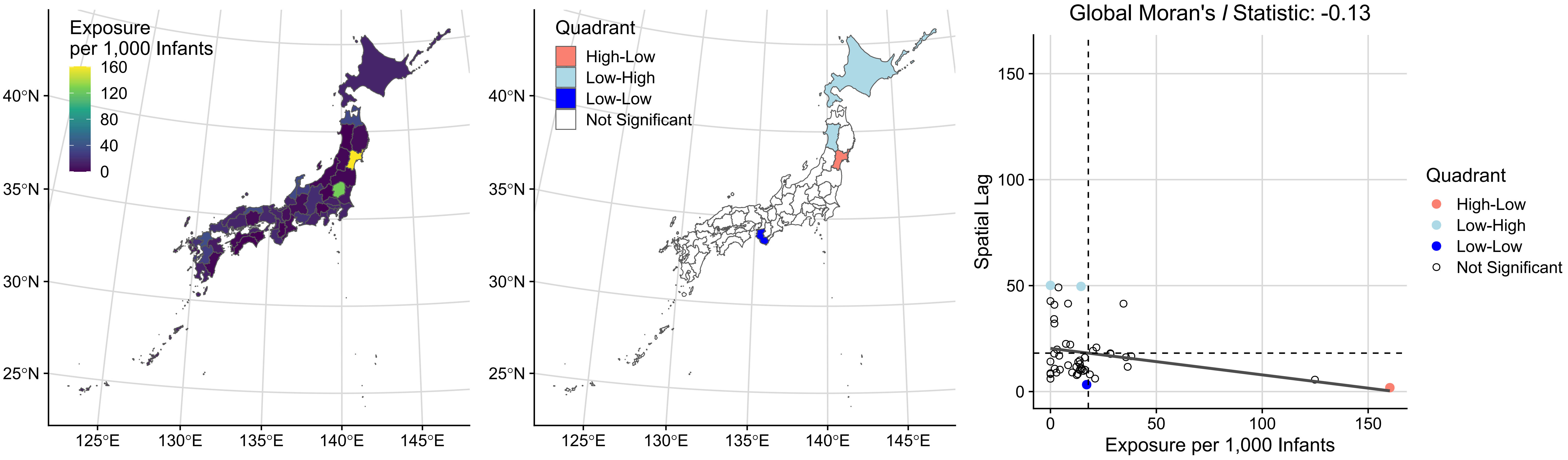

Neonatal Exposure among Very Preterm and Very Low Birth Weight Infants (Days 0–27)

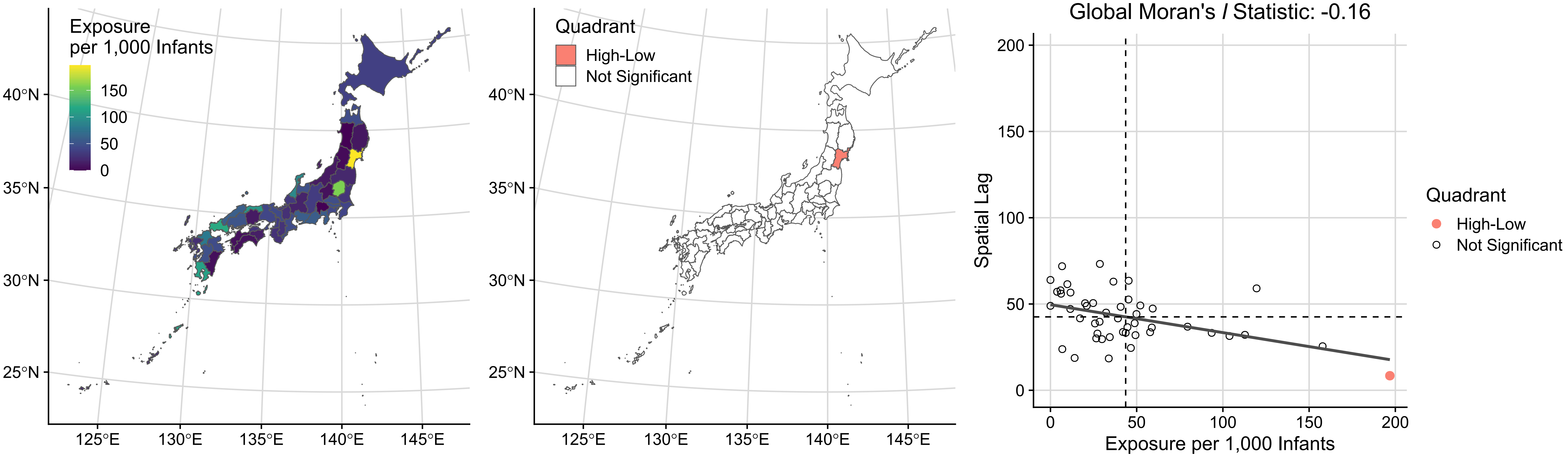

J01EE. Combinations of Sulfonamides and Trimethoprim, Including Derivatives

Early Neonatal Exposure among Very Preterm and Very Low Birth Weight Infants (Days 0–6)

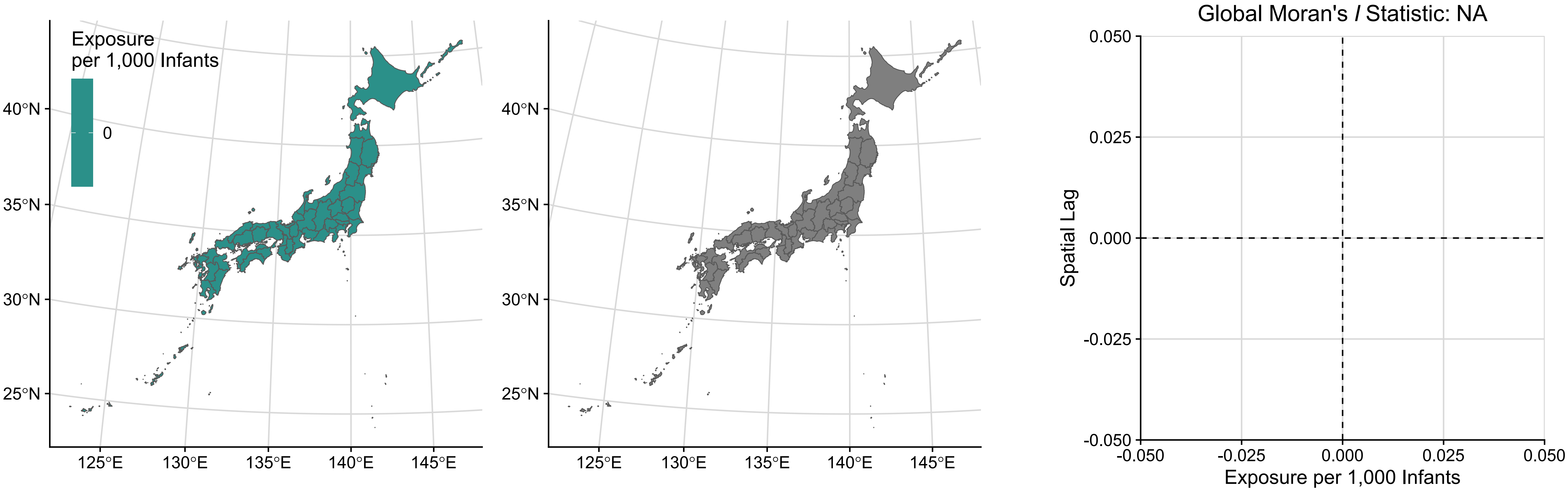

Neonatal Exposure among Very Preterm and Very Low Birth Weight Infants (Days 0–27)

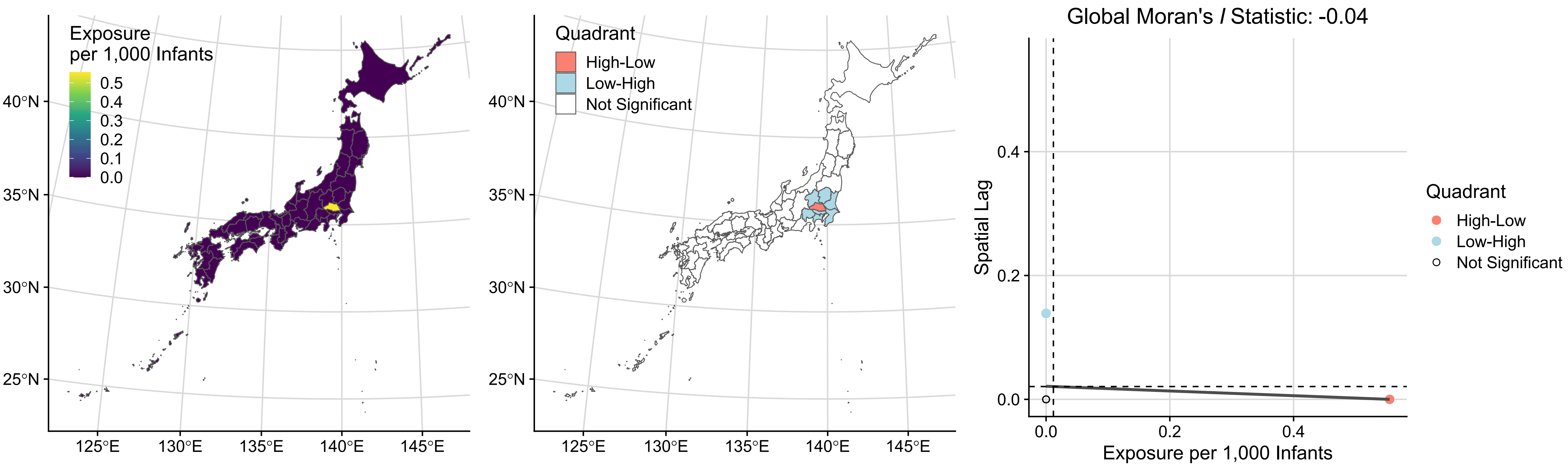

J01FA. Macrolides

Early Neonatal Exposure among Very Preterm and Very Low Birth Weight Infants (Days 0–6)

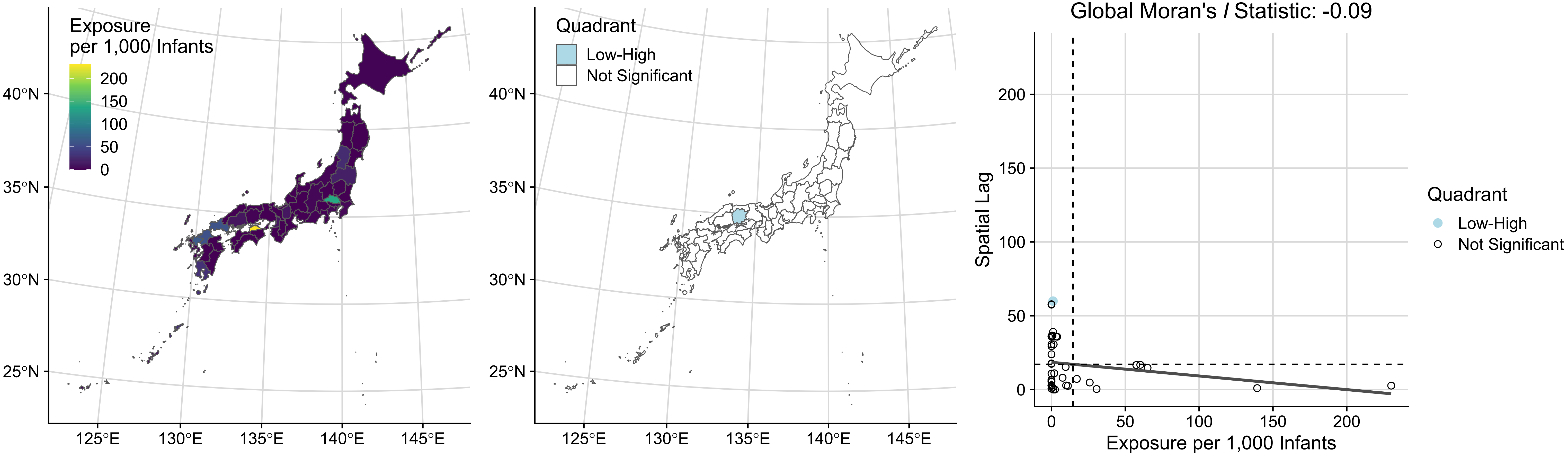

Neonatal Exposure among Very Preterm and Very Low Birth Weight Infants (Days 0–27)

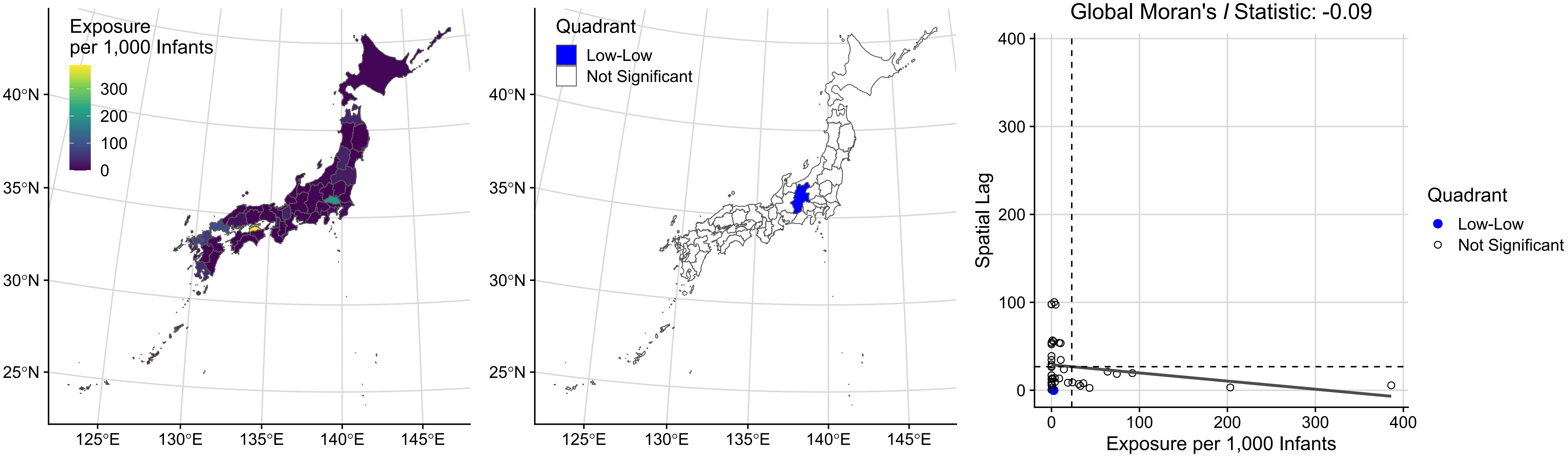

J01FF. Lincosamides

Early Neonatal Exposure among Very Preterm and Very Low Birth Weight Infants (Days 0–6)

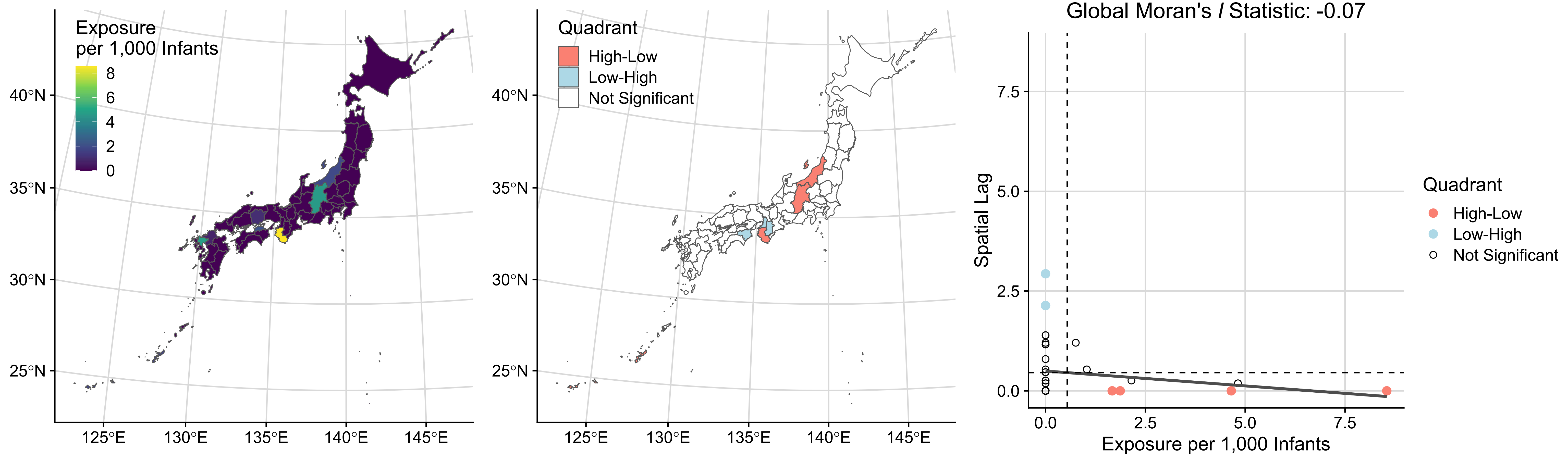

Neonatal Exposure among Very Preterm and Very Low Birth Weight Infants (Days 0–27)

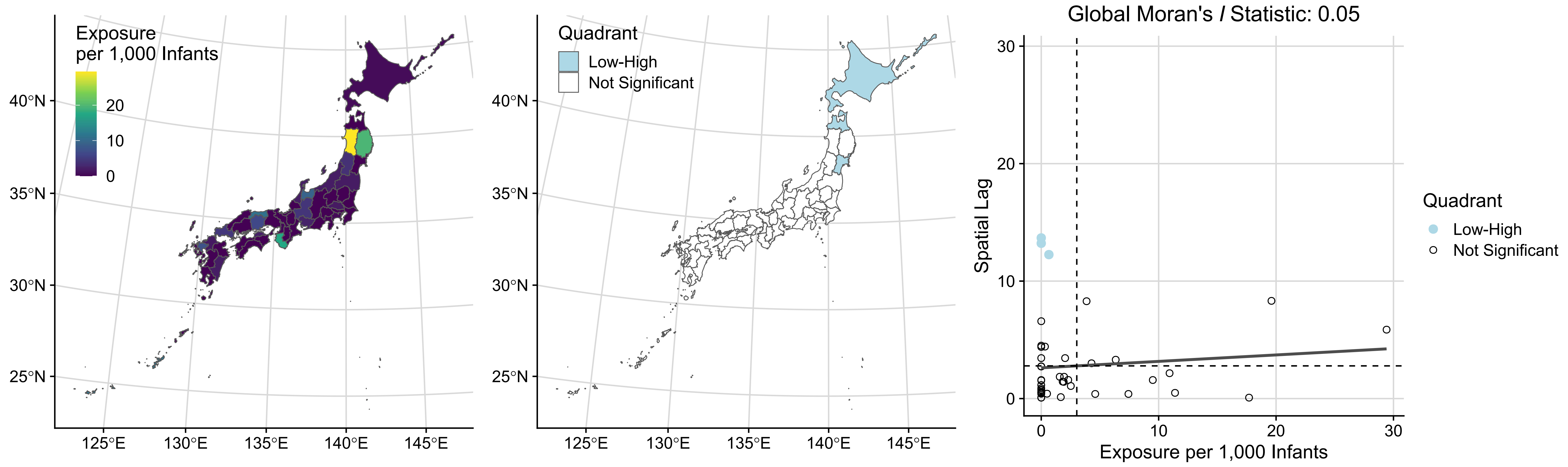

J01GB. Aminoglycosides Other than Streptomycins

Early Neonatal Exposure among Very Preterm and Very Low Birth Weight Infants (Days 0–6)

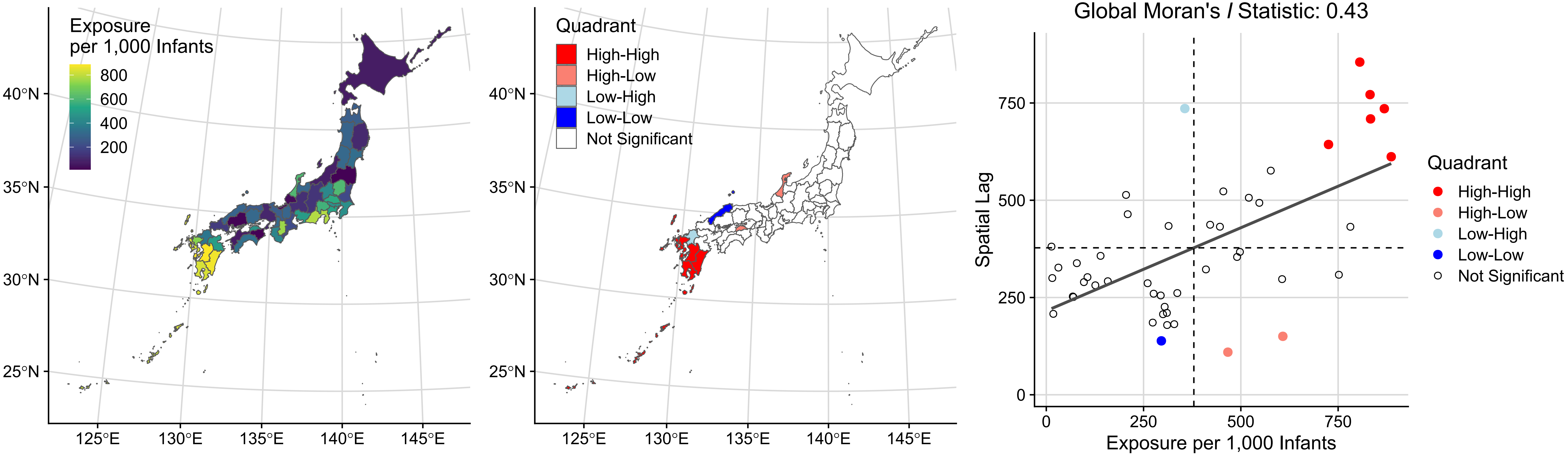

Neonatal Exposure among Very Preterm and Very Low Birth Weight Infants (Days 0–27)

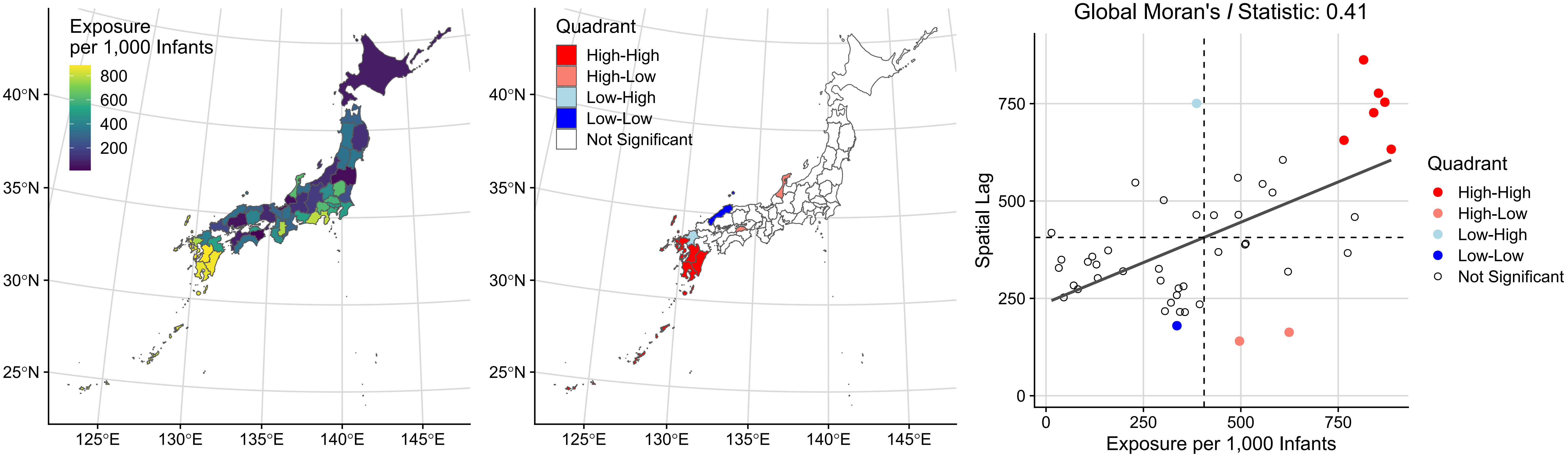

J01MA. Fluoroquinolones

Early Neonatal Exposure among Very Preterm and Very Low Birth Weight Infants (Days 0–6)

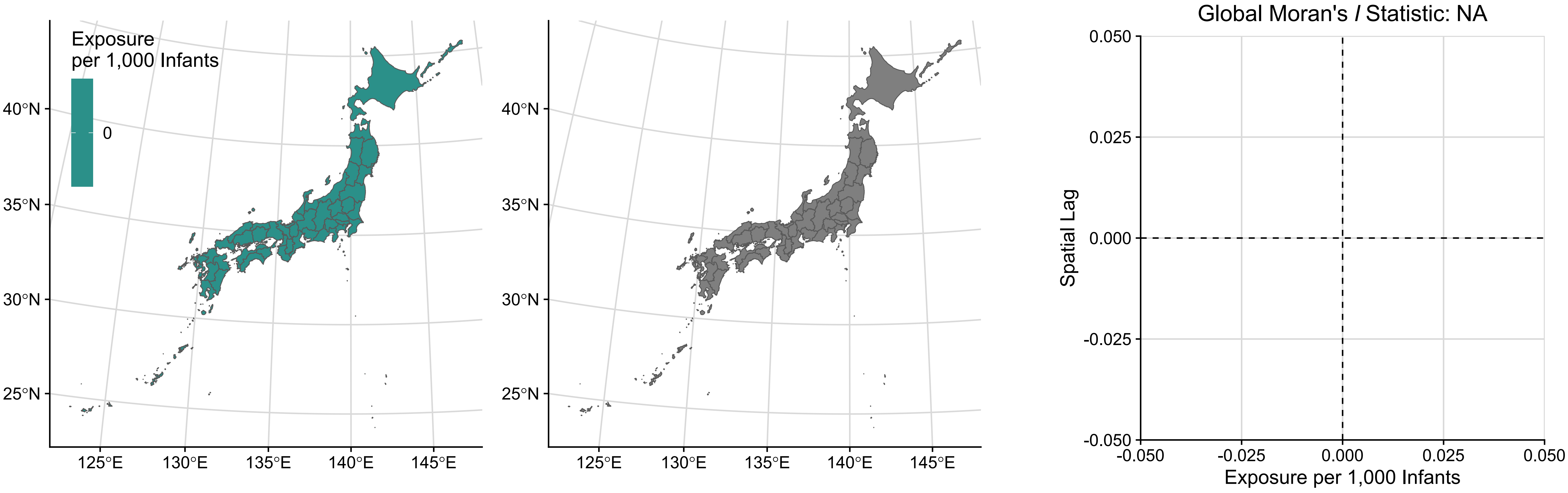

Neonatal Exposure among Very Preterm and Very Low Birth Weight Infants (Days 0–27)

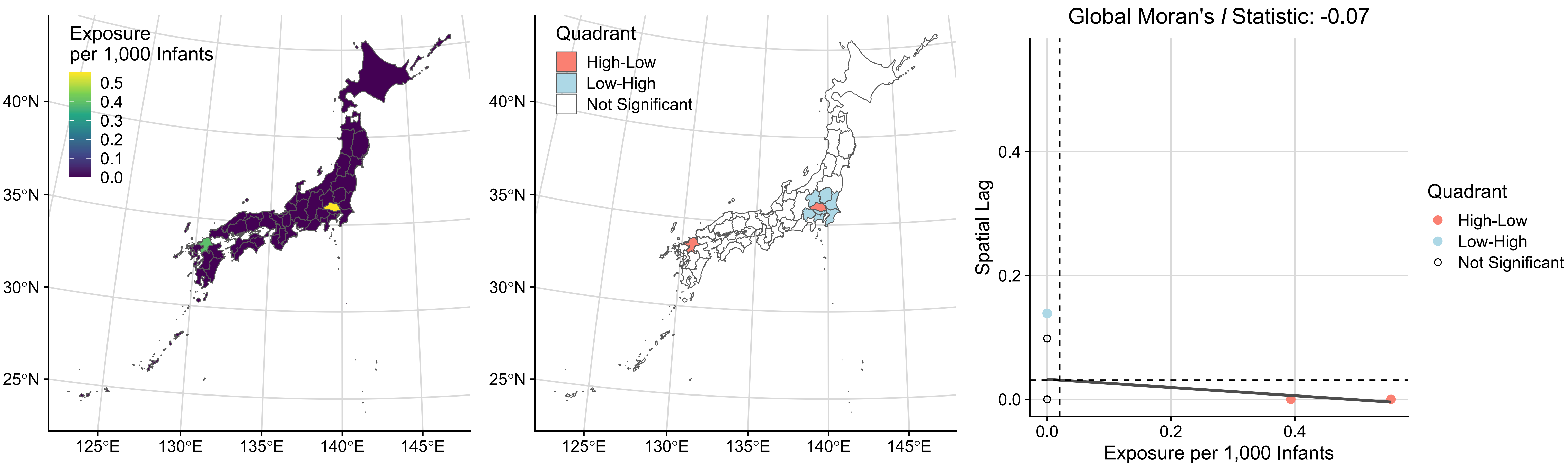

J01XA. Glycopeptide Antibacterials

Early Neonatal Exposure among Very Preterm and Very Low Birth Weight Infants (Days 0–6)

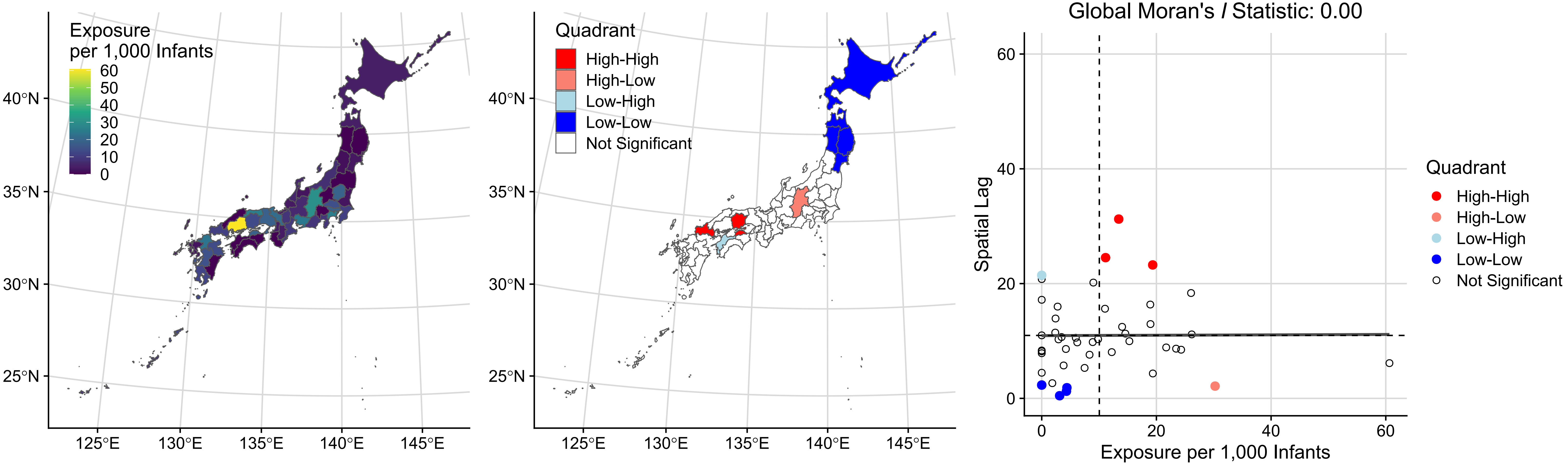

Neonatal Exposure among Very Preterm and Very Low Birth Weight Infants (Days 0–27)

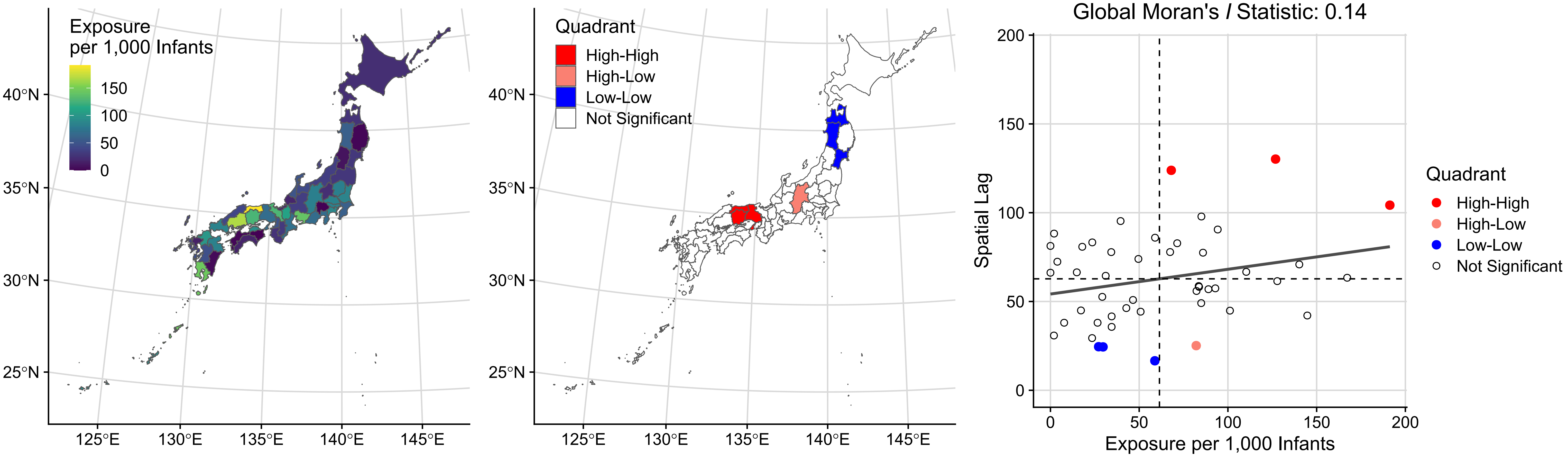

J01XD. Imidazole Derivatives

Early Neonatal Exposure among Very Preterm and Very Low Birth Weight Infants (Days 0–6)

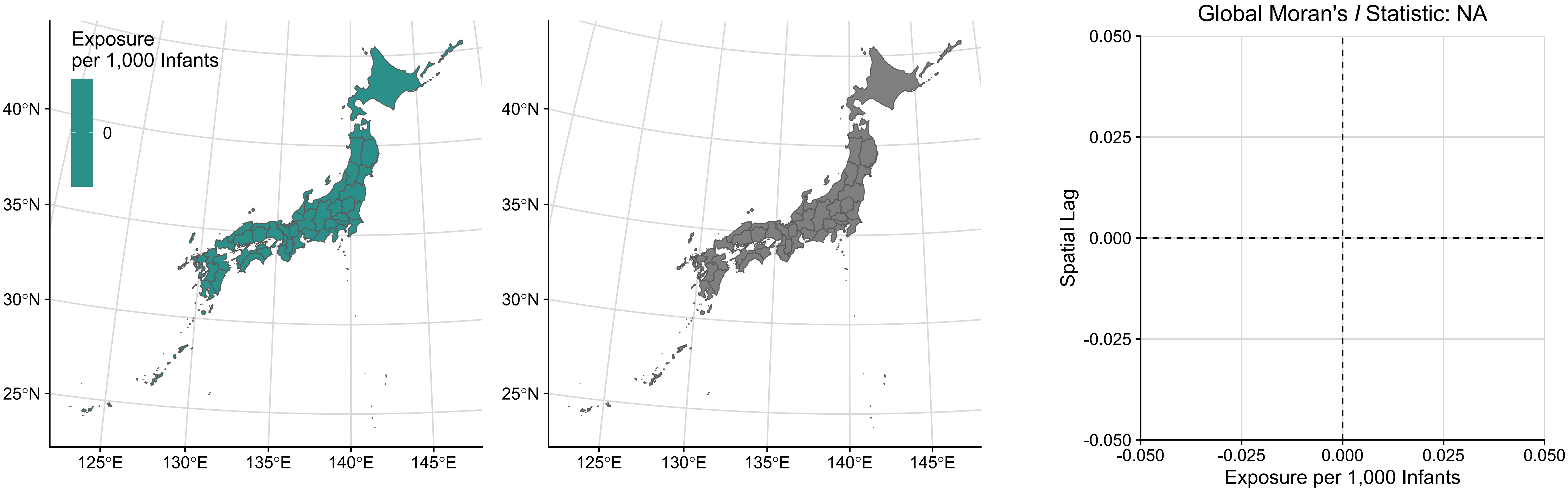

Neonatal Exposure among Very Preterm and Very Low Birth Weight Infants (Days 0–27)

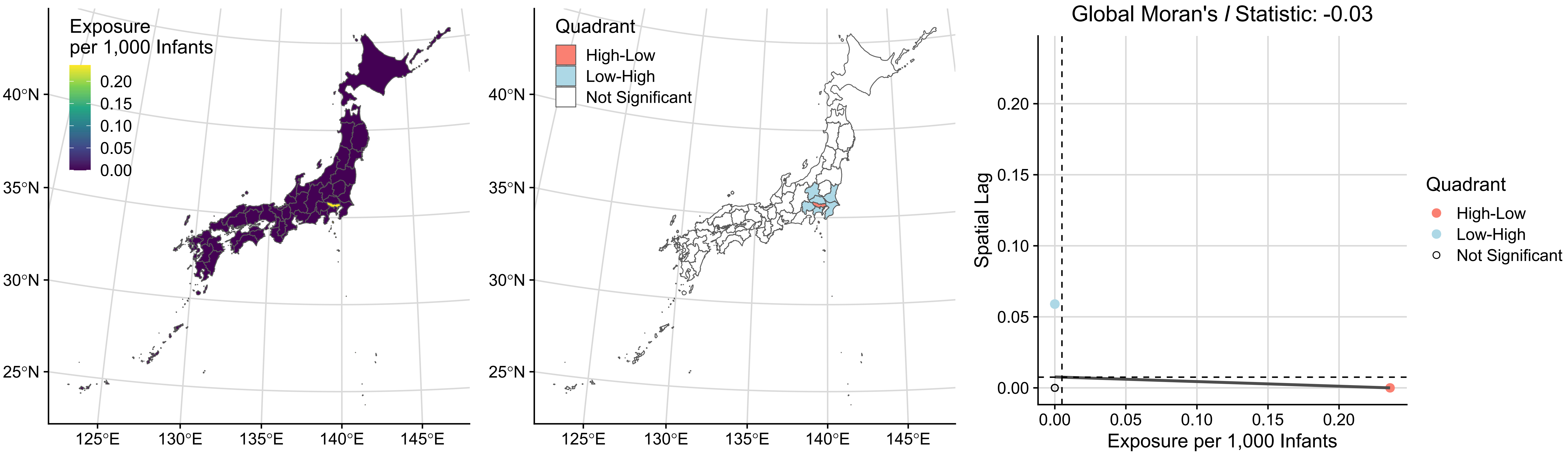

J01XX. Other Antibacterials

Early Neonatal Exposure among Very Preterm and Very Low Birth Weight Infants (Days 0–6)

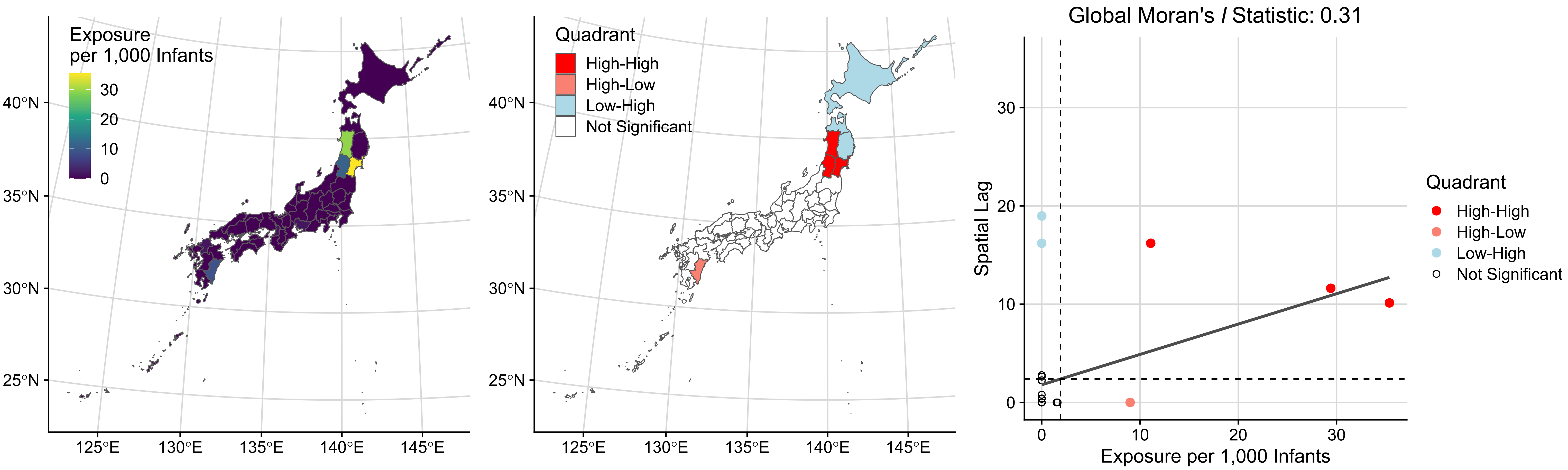

Neonatal Exposure among Very Preterm and Very Low Birth Weight Infants (Days 0–27)

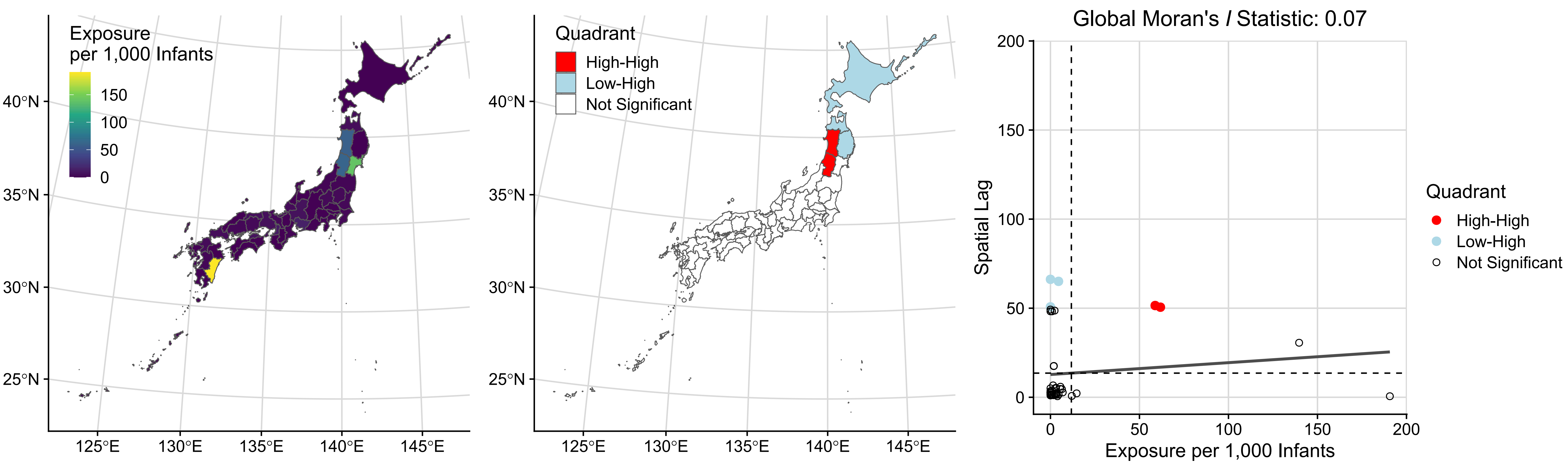

J02AA. Antibiotics

Early Neonatal Exposure among Very Preterm and Very Low Birth Weight Infants (Days 0–6)

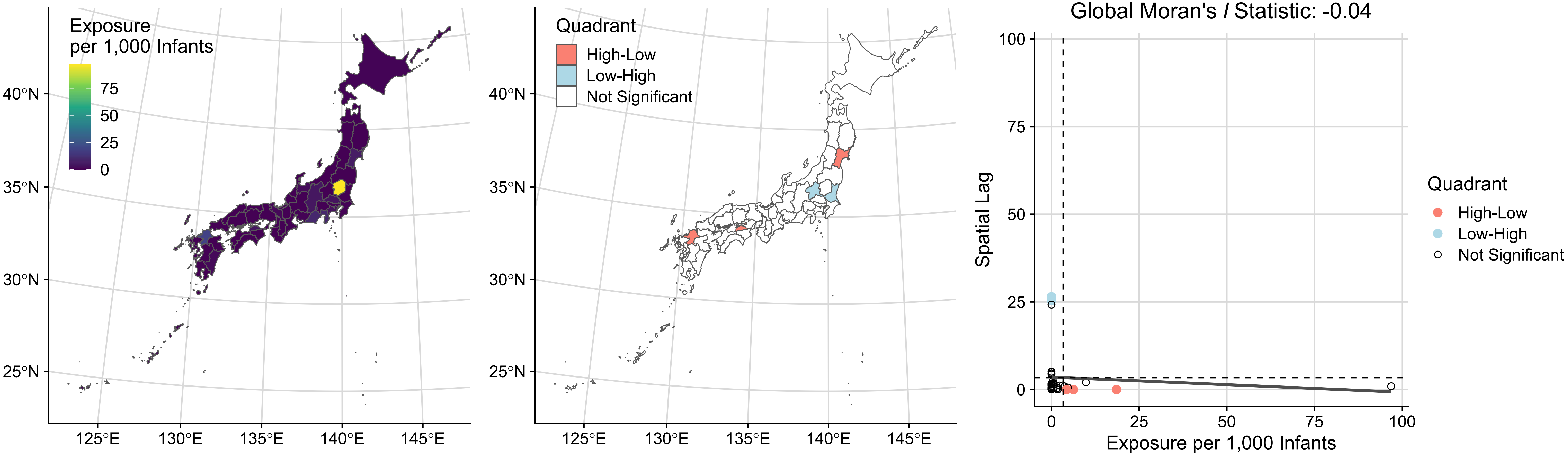

Neonatal Exposure among Very Preterm and Very Low Birth Weight Infants (Days 0–27)

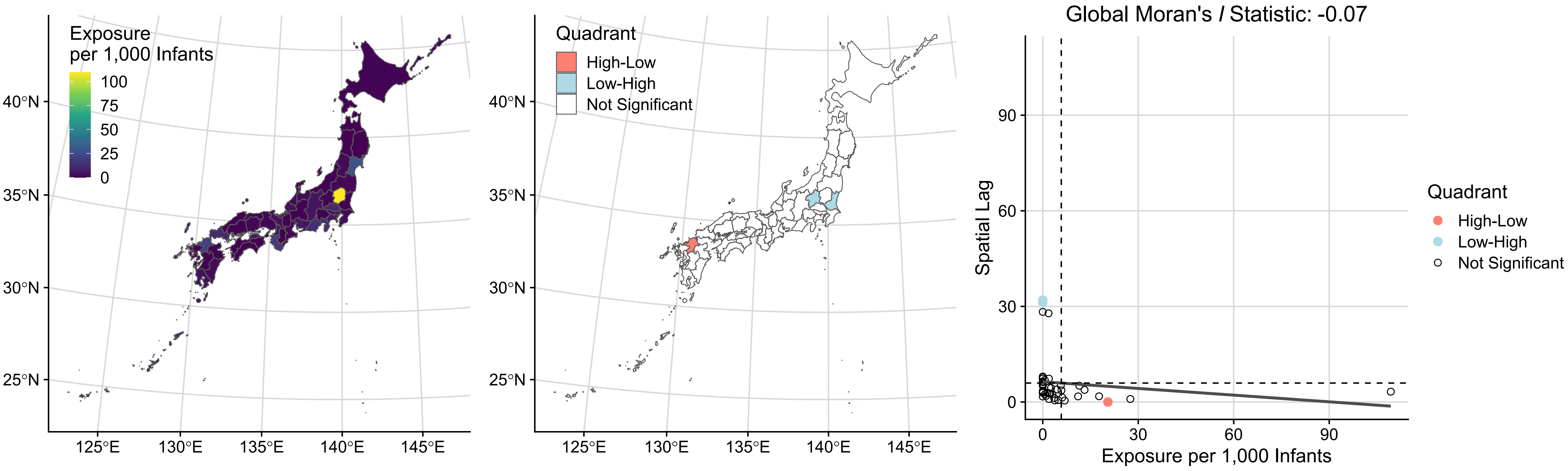

J02AB. Imidazole Derivatives

Early Neonatal Exposure among Very Preterm and Very Low Birth Weight Infants (Days 0–6)

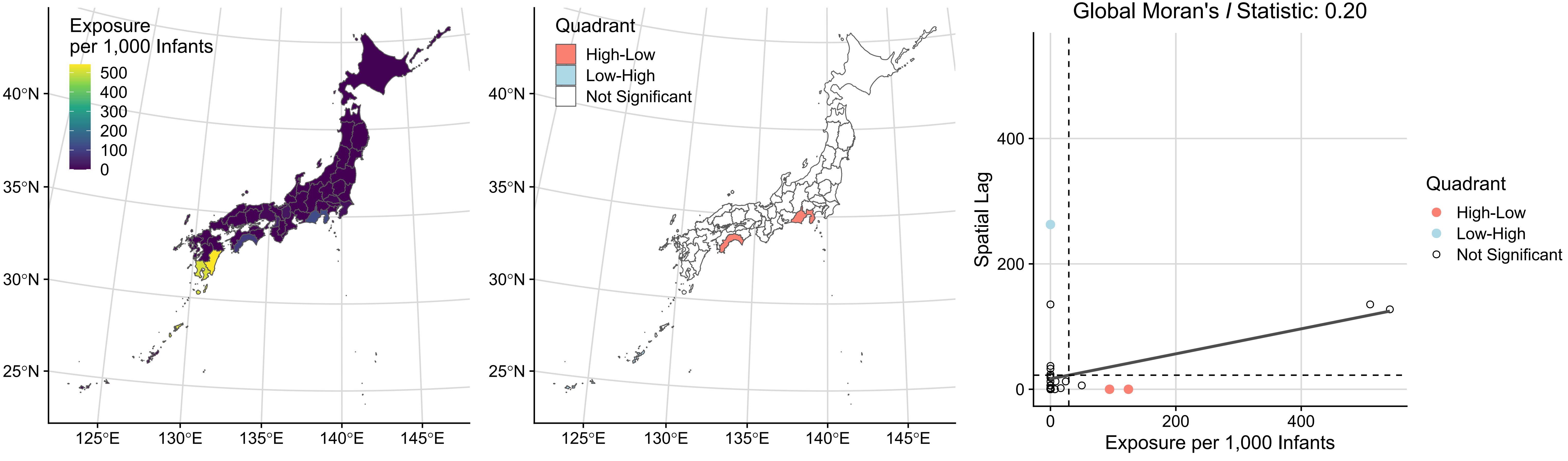

Neonatal Exposure among Very Preterm and Very Low Birth Weight Infants (Days 0–27)

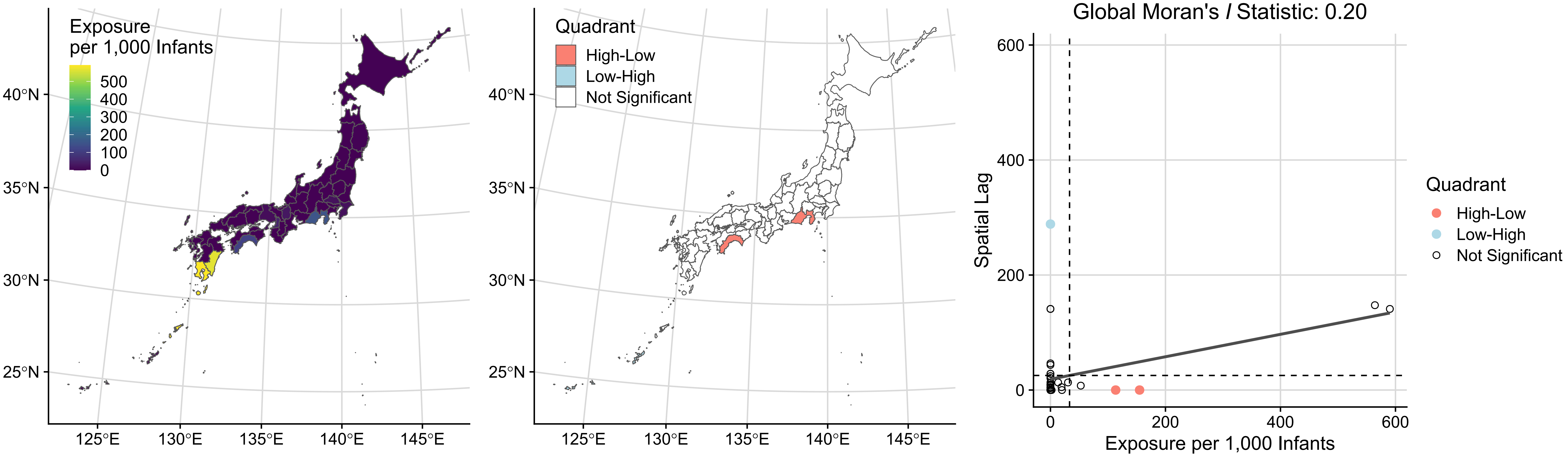

J02AC. Triazole and Tetrazole Derivatives

Early Neonatal Exposure among Very Preterm and Very Low Birth Weight Infants (Days 0–6)

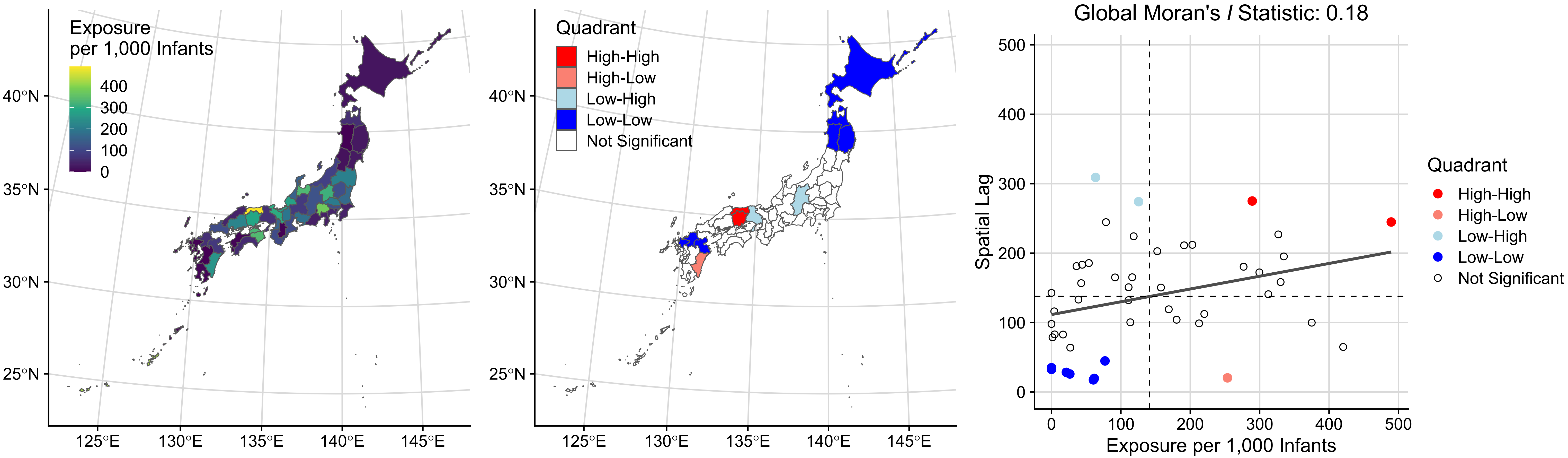

Neonatal Exposure among Very Preterm and Very Low Birth Weight Infants (Days 0–27)

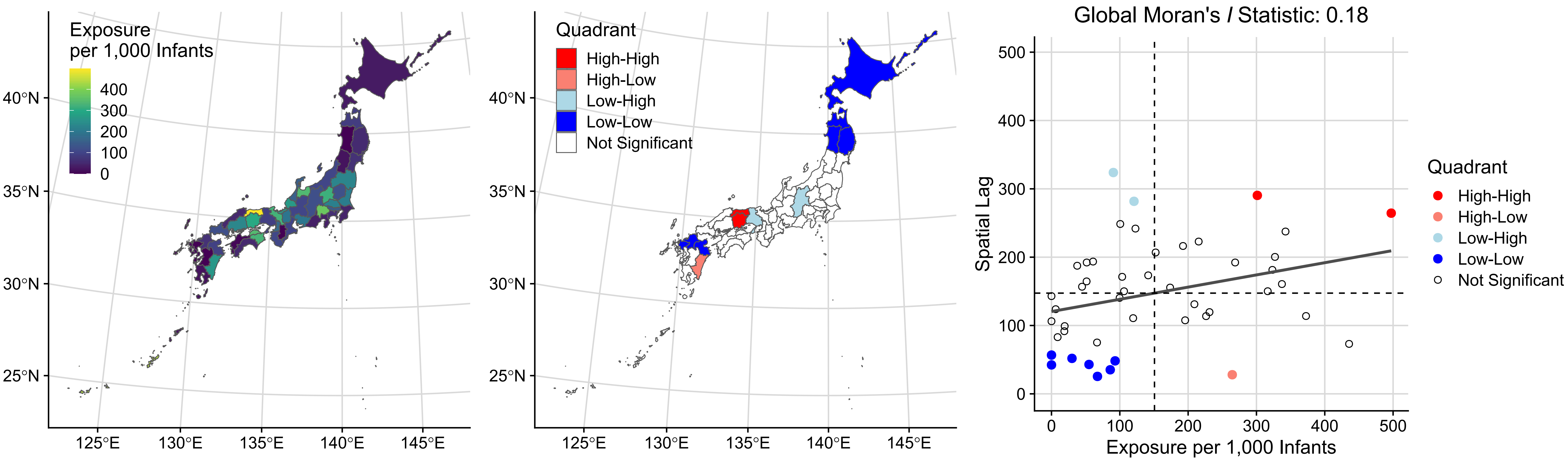

J02AX. Other Antimycotics for Systemic Use

Early Neonatal Exposure among Very Preterm and Very Low Birth Weight Infants (Days 0–6)

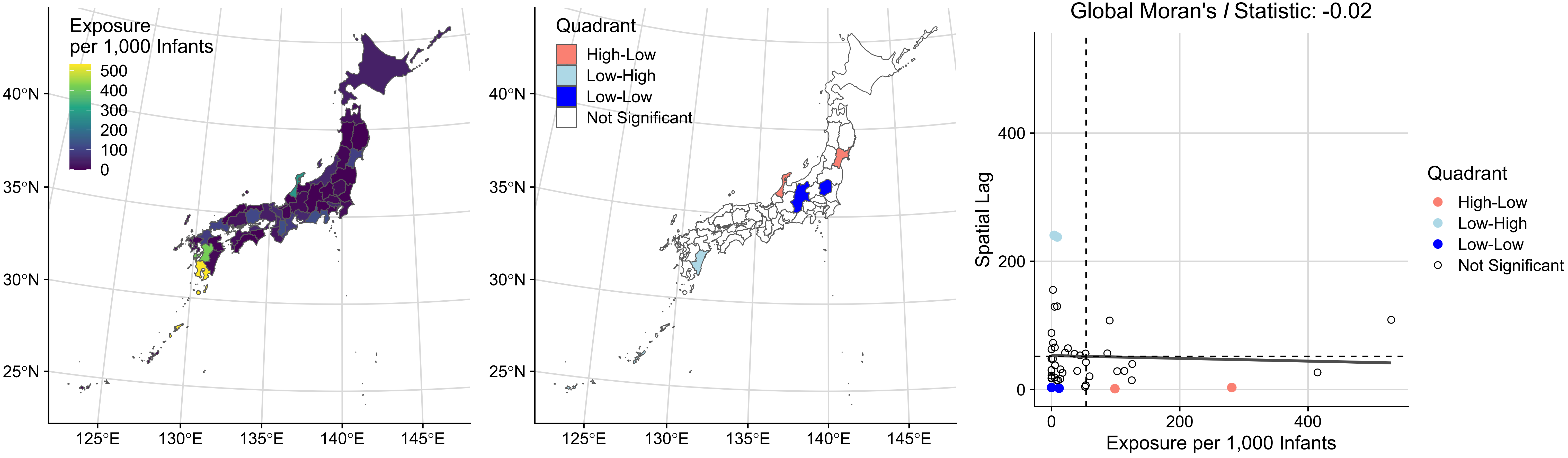

Neonatal Exposure among Very Preterm and Very Low Birth Weight Infants (Days 0–27)

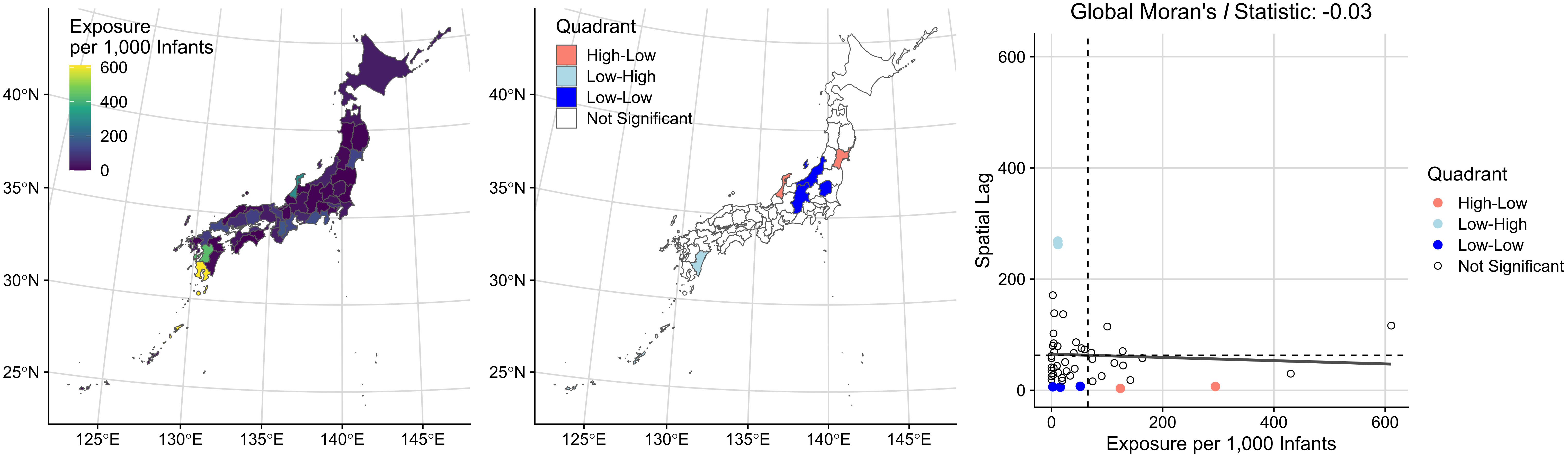

J04AC. Hydrazides

Early Neonatal Exposure among Very Preterm and Very Low Birth Weight Infants (Days 0–6)

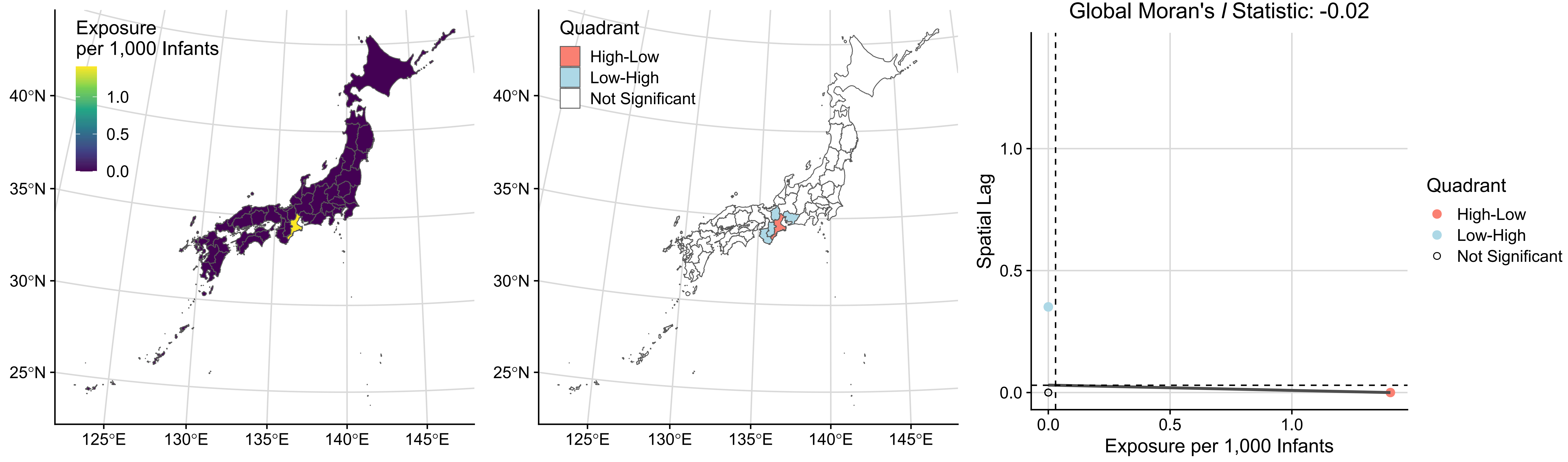

Neonatal Exposure among Very Preterm and Very Low Birth Weight Infants (Days 0–27)

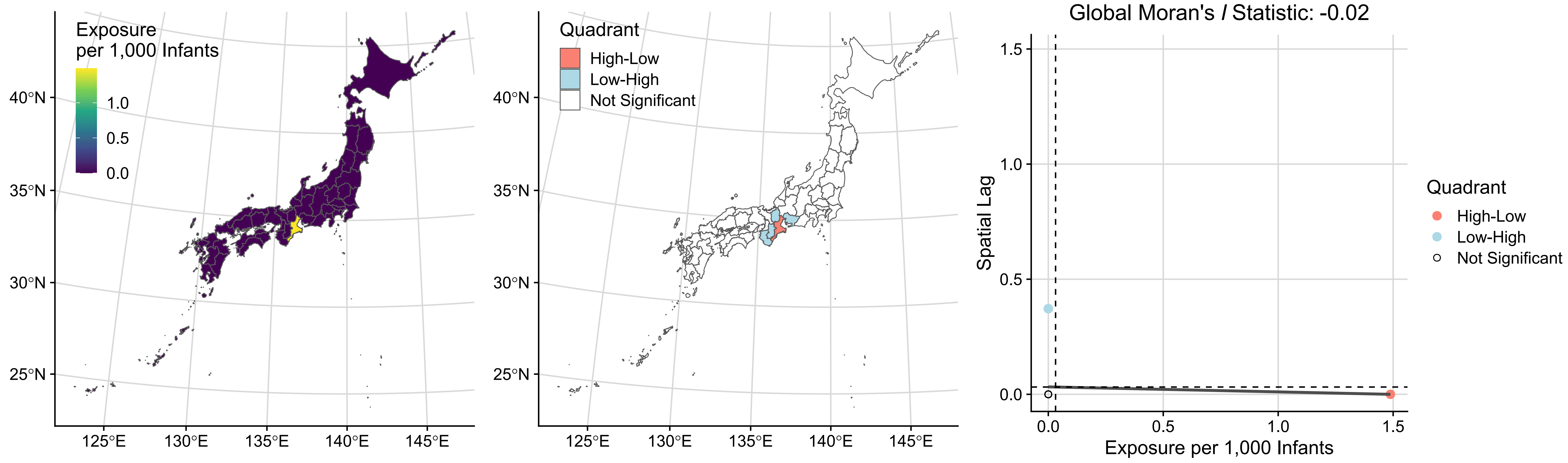

J05AB. Nucleosides and Nucleotides Excluding Reverse Transcriptase Inhibitors

Early Neonatal Exposure among Very Preterm and Very Low Birth Weight Infants (Days 0–6)

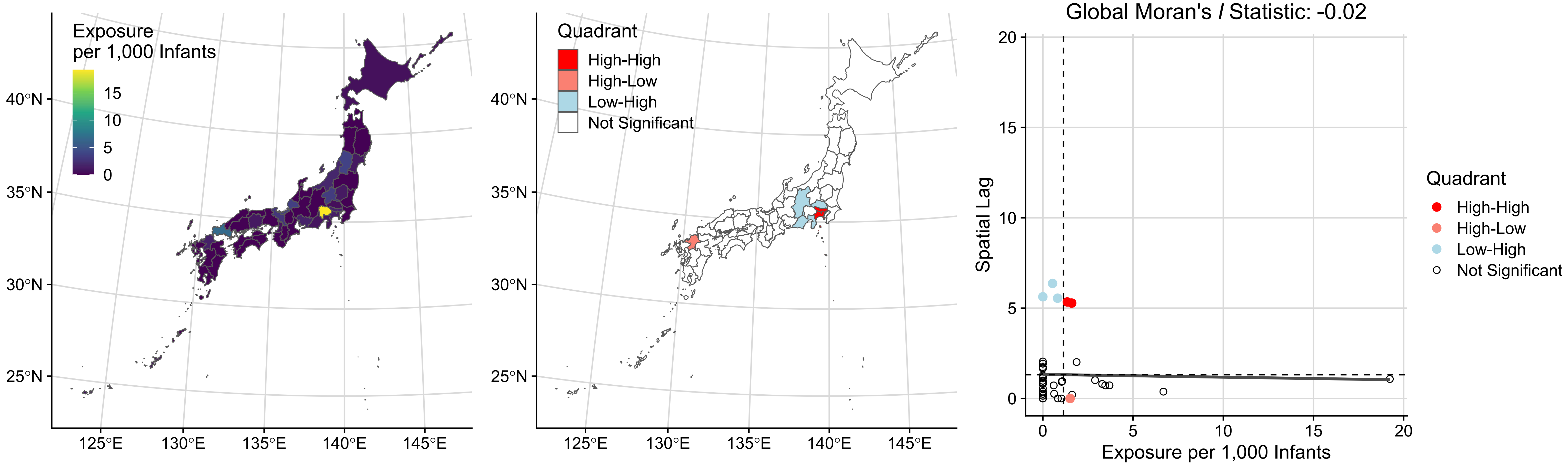

Neonatal Exposure among Very Preterm and Very Low Birth Weight Infants (Days 0–27)

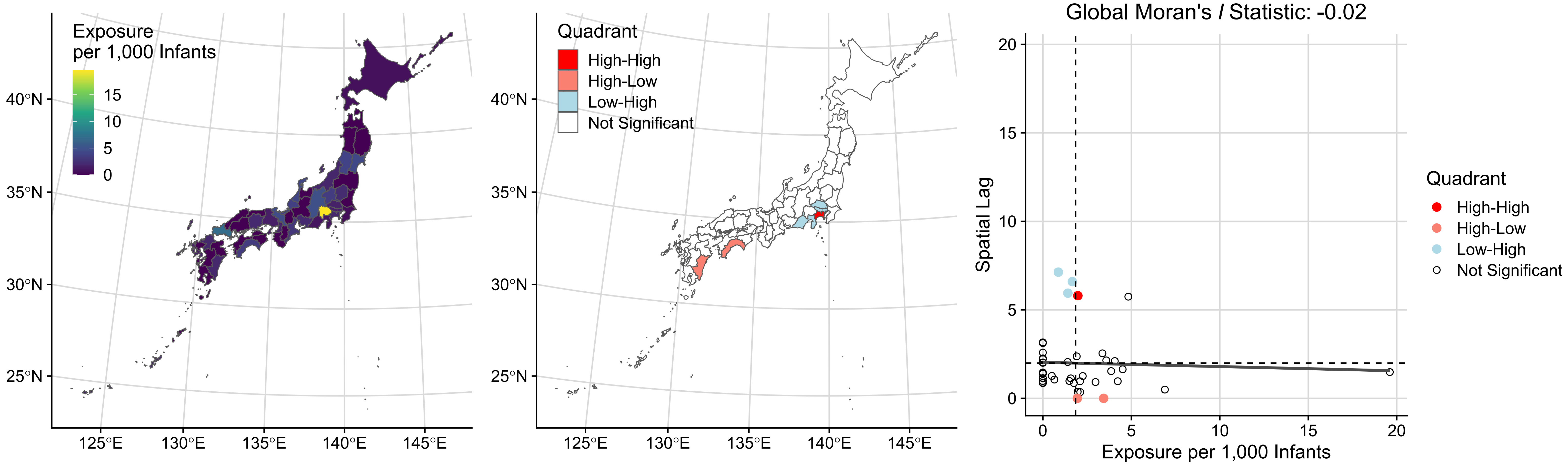

J05AH. Neuraminidase Inhibitors

Early Neonatal Exposure among Very Preterm and Very Low Birth Weight Infants (Days 0–6)

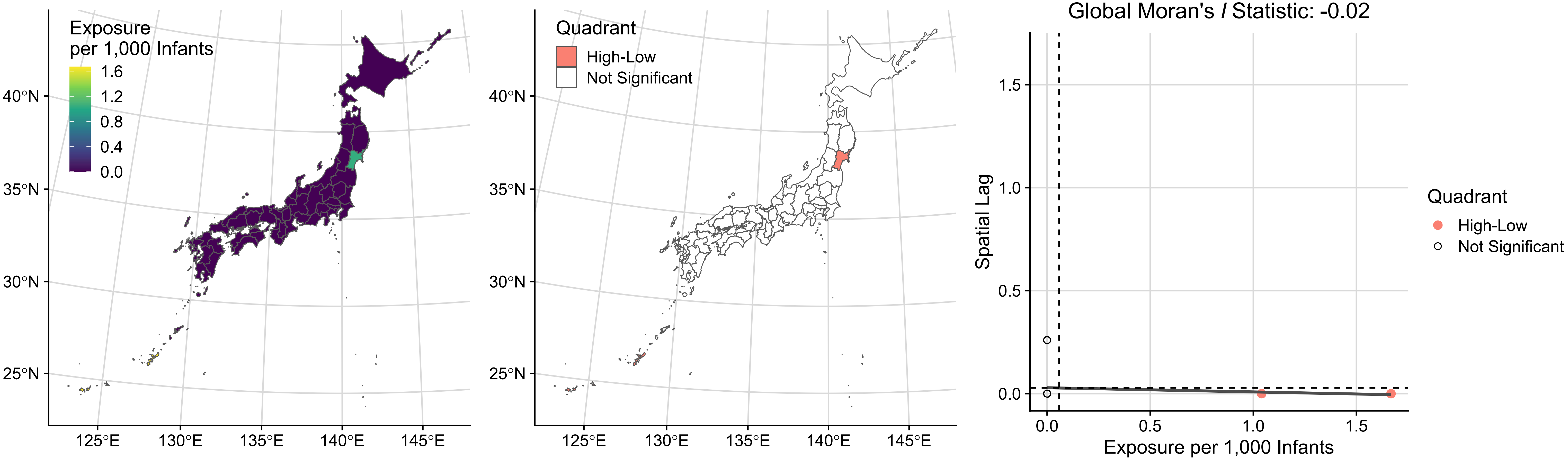

Neonatal Exposure among Very Preterm and Very Low Birth Weight Infants (Days 0–27)

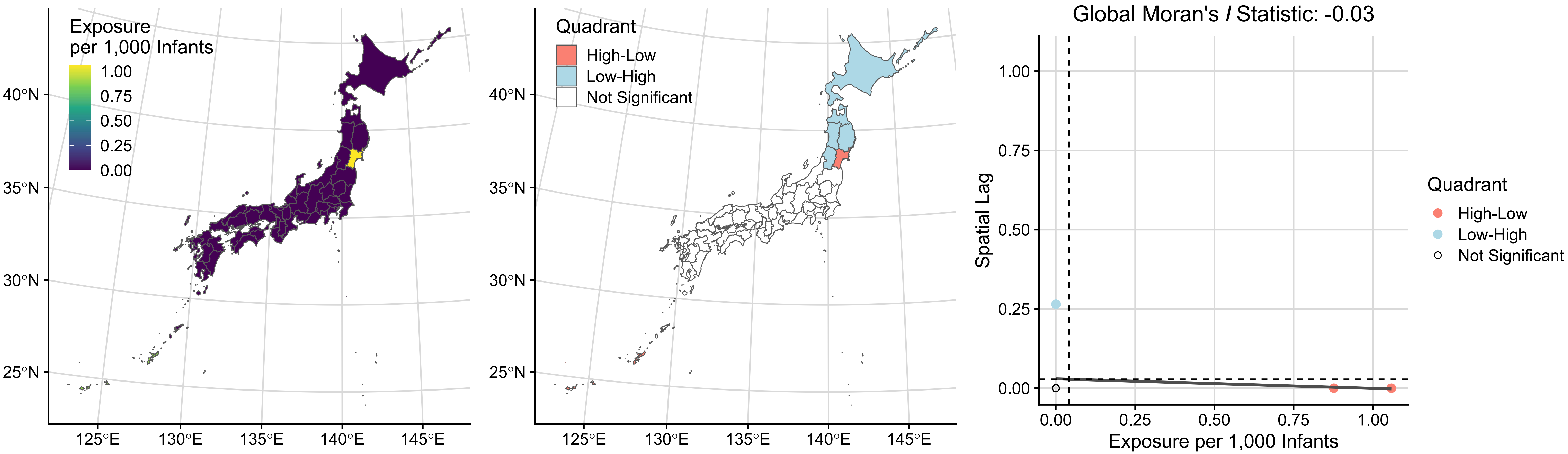

Supplement: S2 Fig — (PDF) [file pone.0295528.s003.pdf]
