# Supplementary material for: Geographical distribution of antimicrobial exposure among very preterm and very low birth weight infants: A nationwide database study in Japan
Source: PLoS One. 2024 Jan 25;19(1):e0295528. doi: 10.1371/journal.pone.0295528 (PMC10810499; doi:10.1371/journal.pone.0295528)

A. Early and Late Neonatal Episodes of Antibacterial Administration per 1,000 Infants

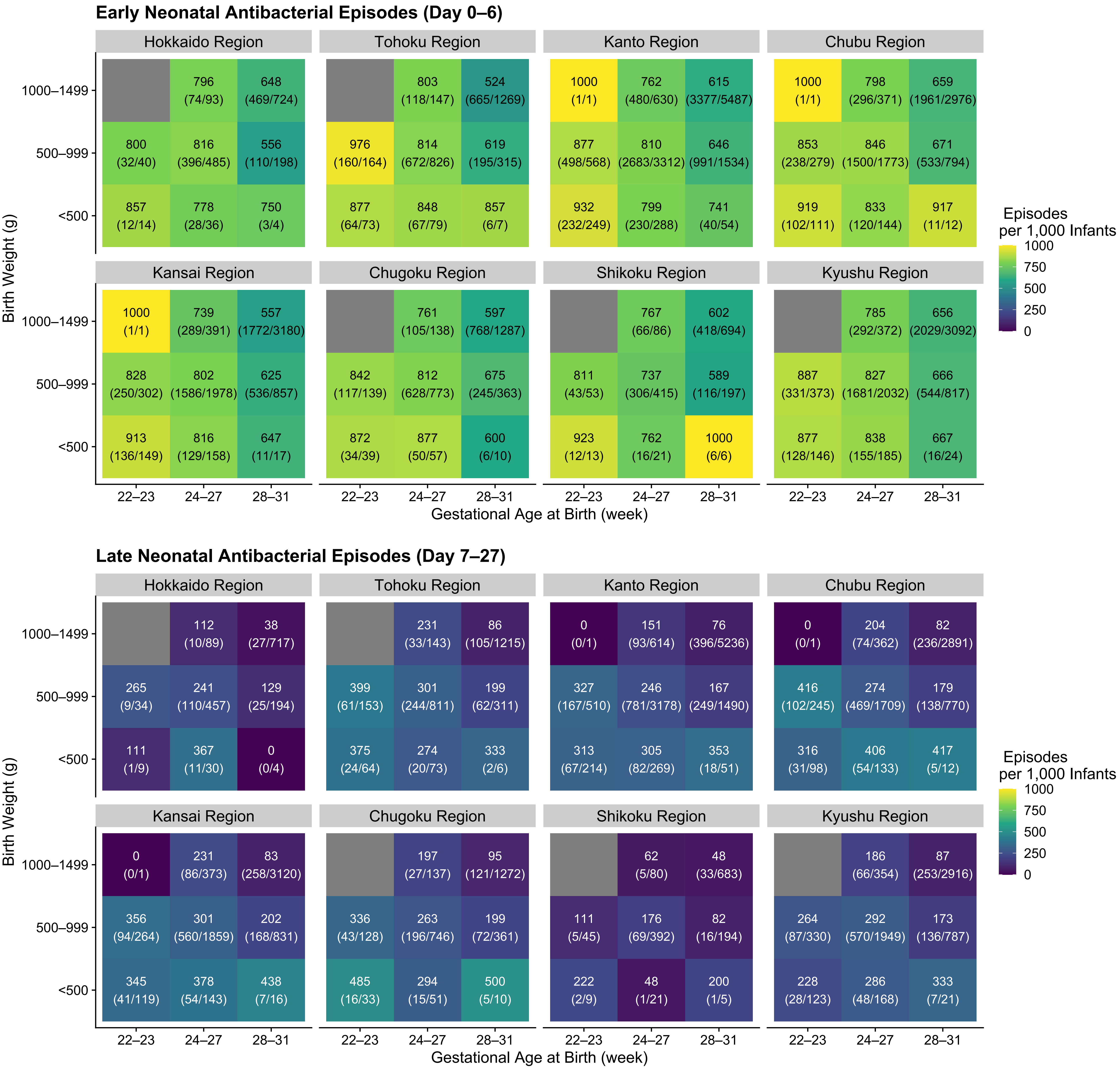

B. Drug Selection Rates on the First Day of Early Neonatal Courses of Antibacterial Administration (Day 0–6)

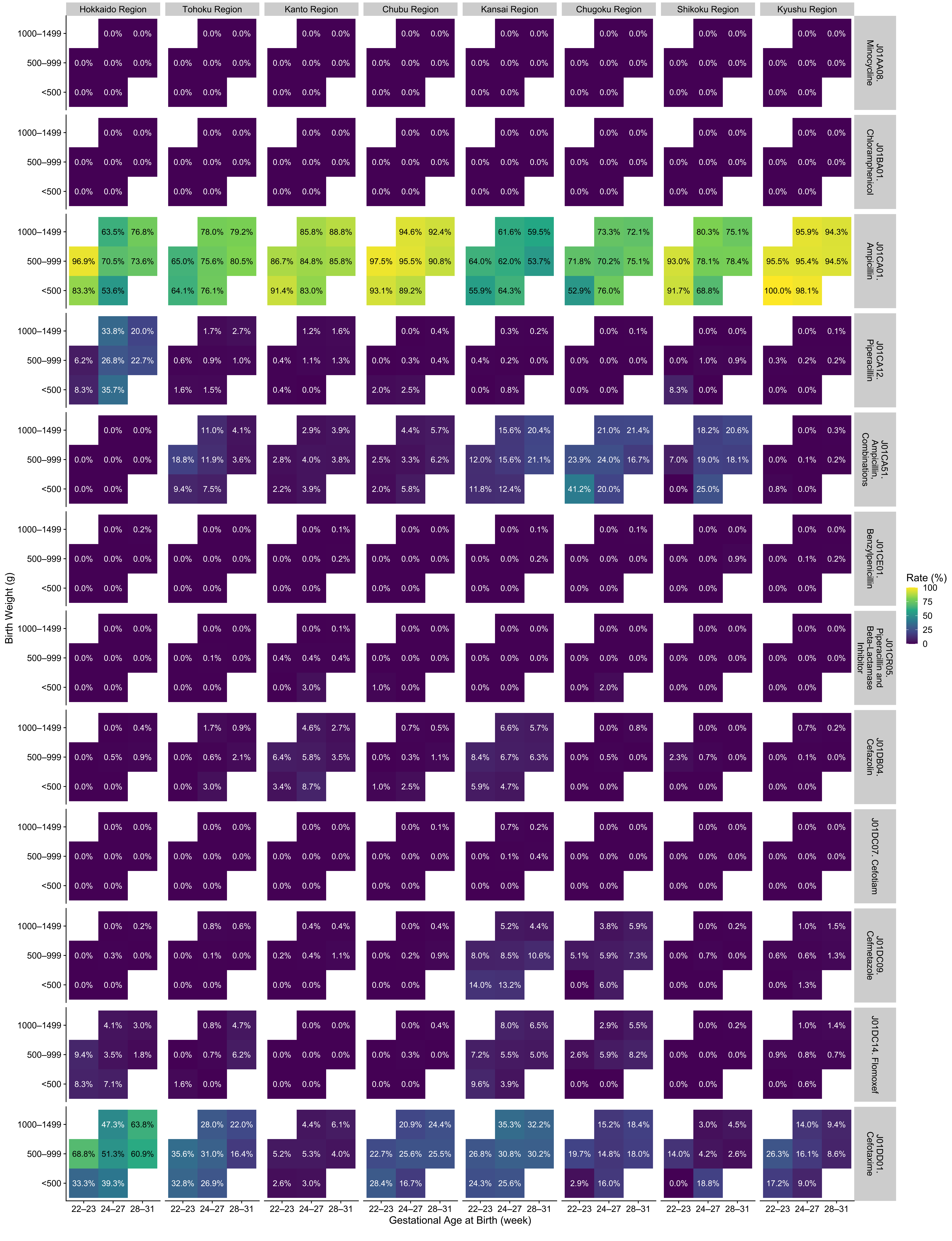

B. Drug Selection Rates on the First Day of Early Neonatal Courses of Antibacterial Administration (Day 0–6), Continued

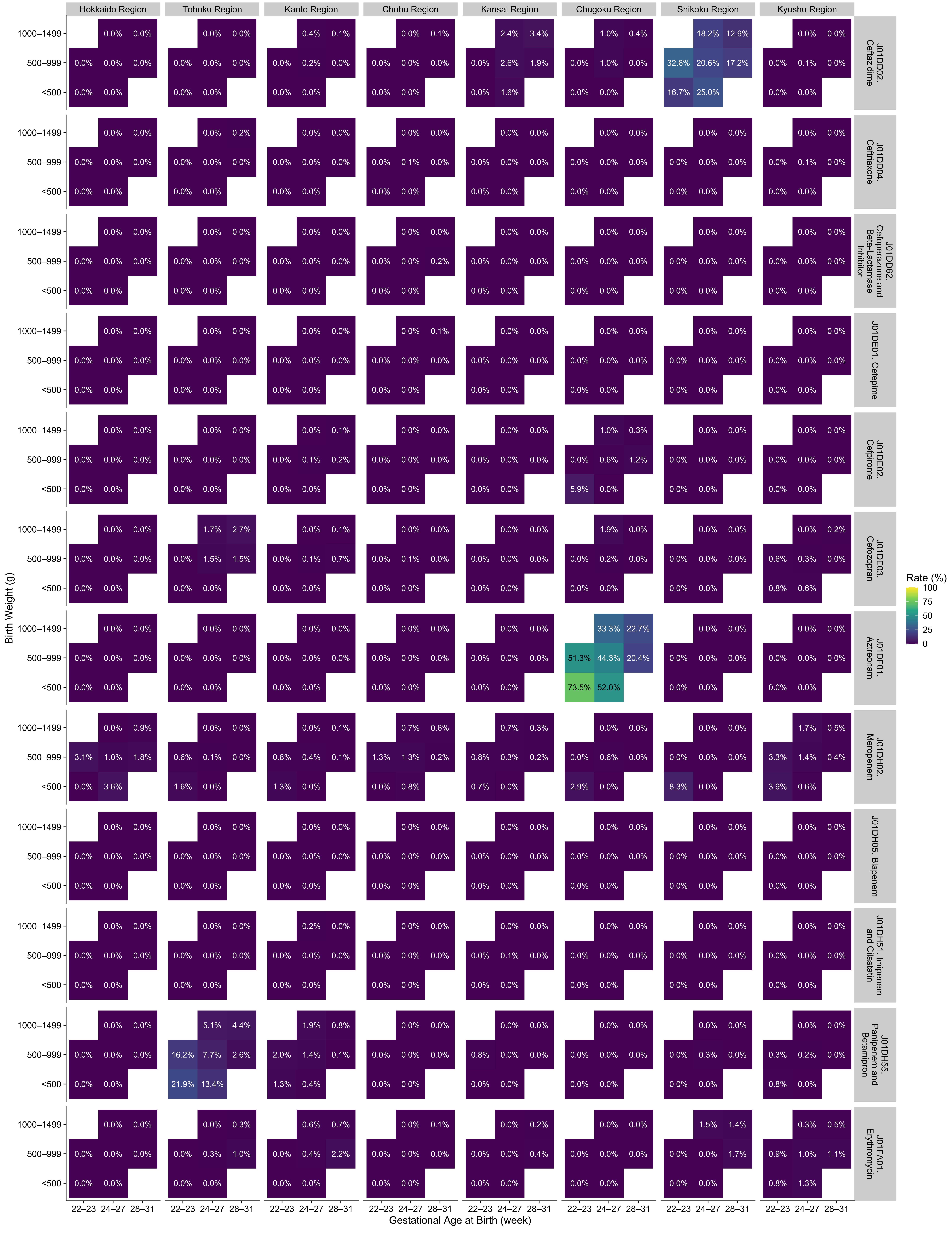

B. Drug Selection Rates on the First Day of Early Neonatal Courses of Antibacterial Administration (Day 0–6), Continued

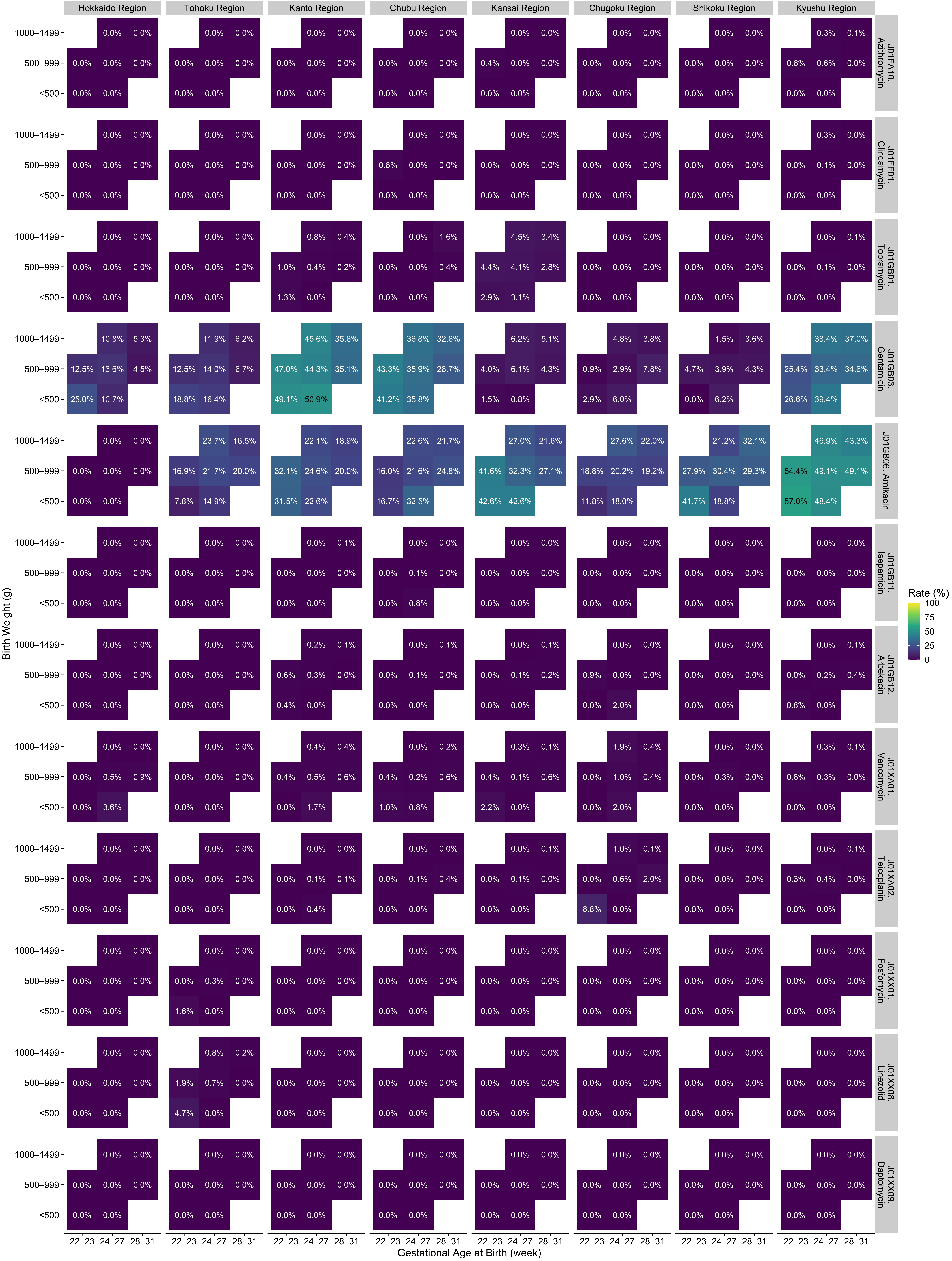

C. Drug Selection Rates on the First Day of Late Neonatal Courses of Antibacterial Administration (Day 7–27)

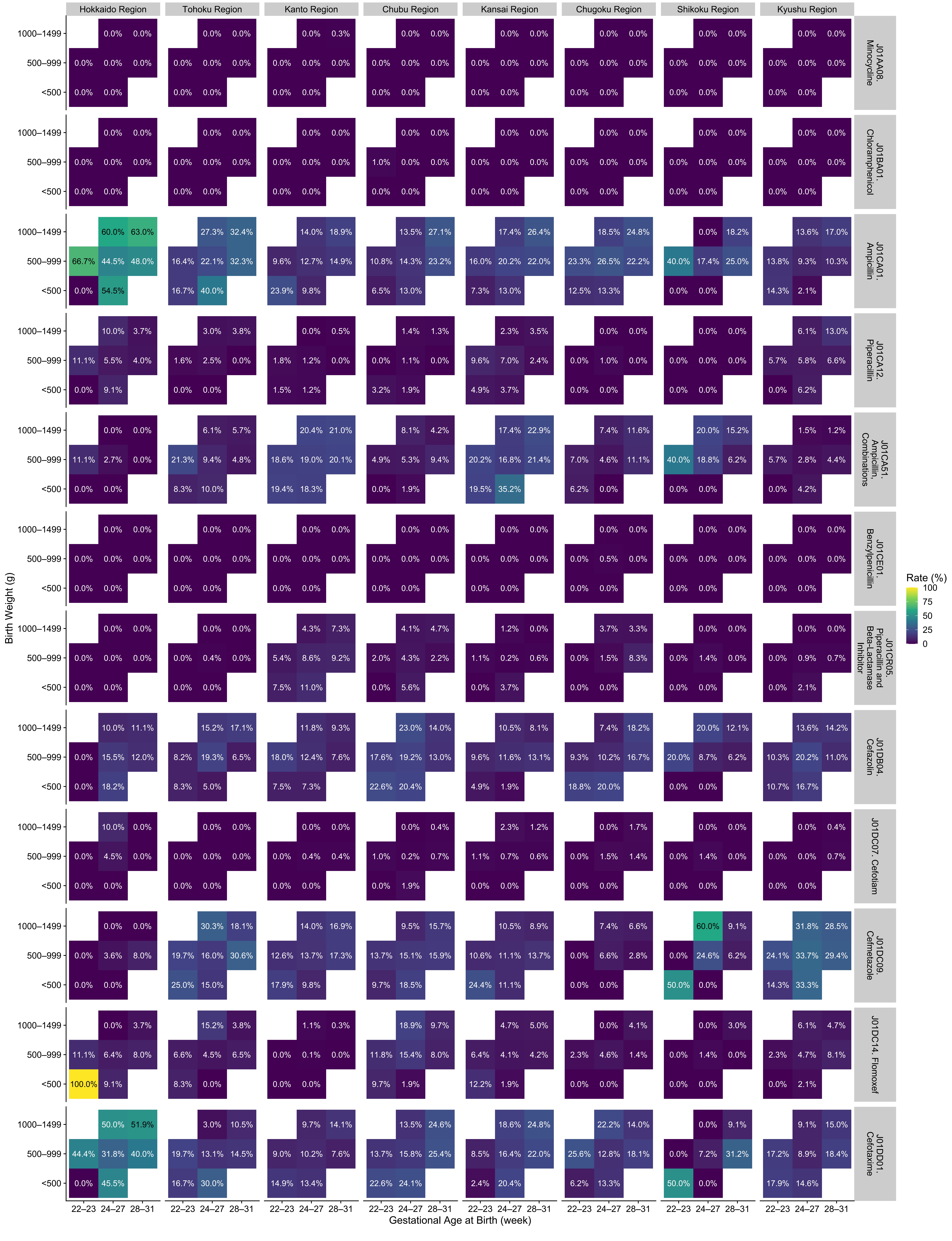

C. Drug Selection Rates on the First Day of Late Neonatal Courses of Antibacterial Administration (Day 7–27), Continued

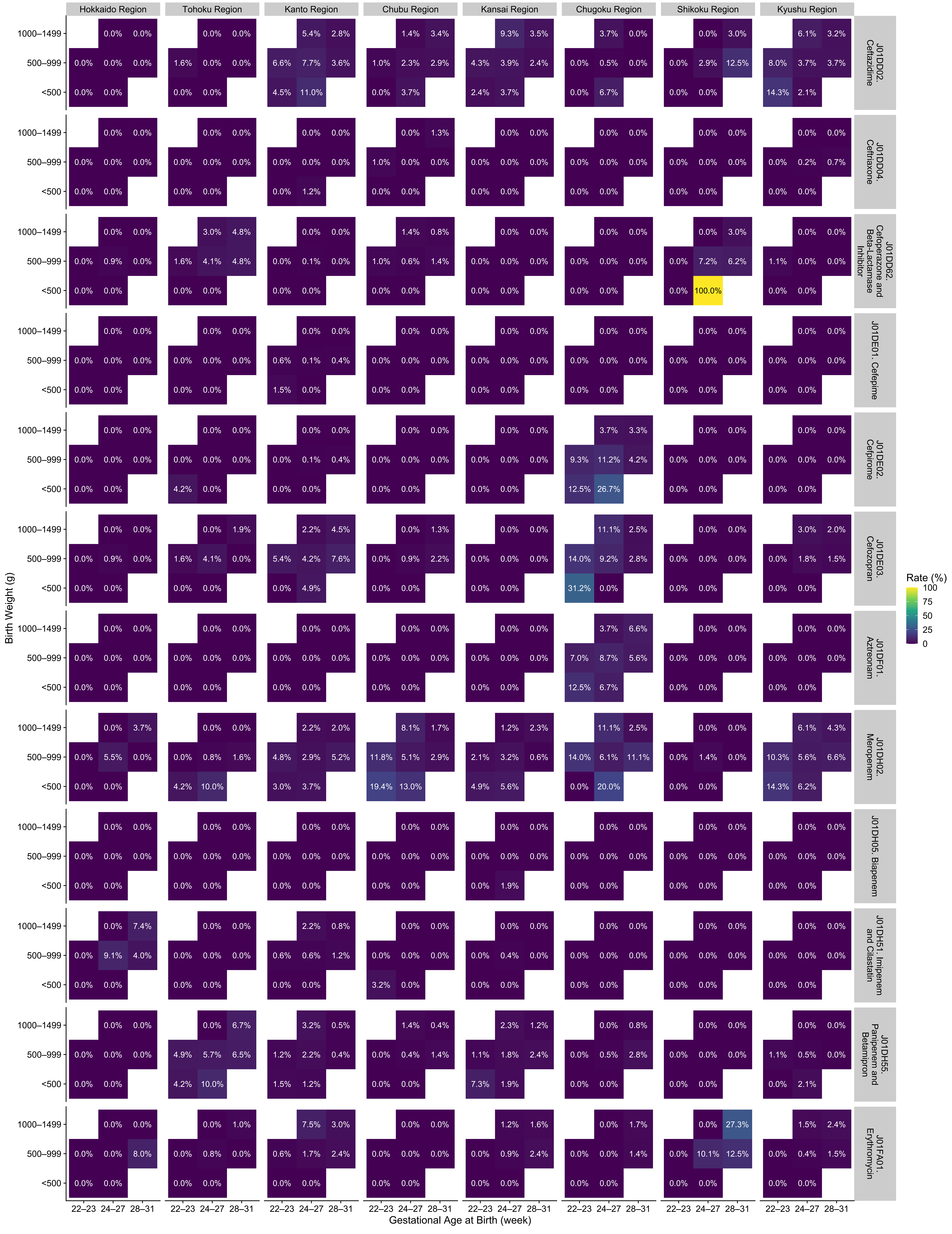

C. Drug Selection Rates on the First Day of Late Neonatal Courses of Antibacterial Administration (Day 7–27), Continued

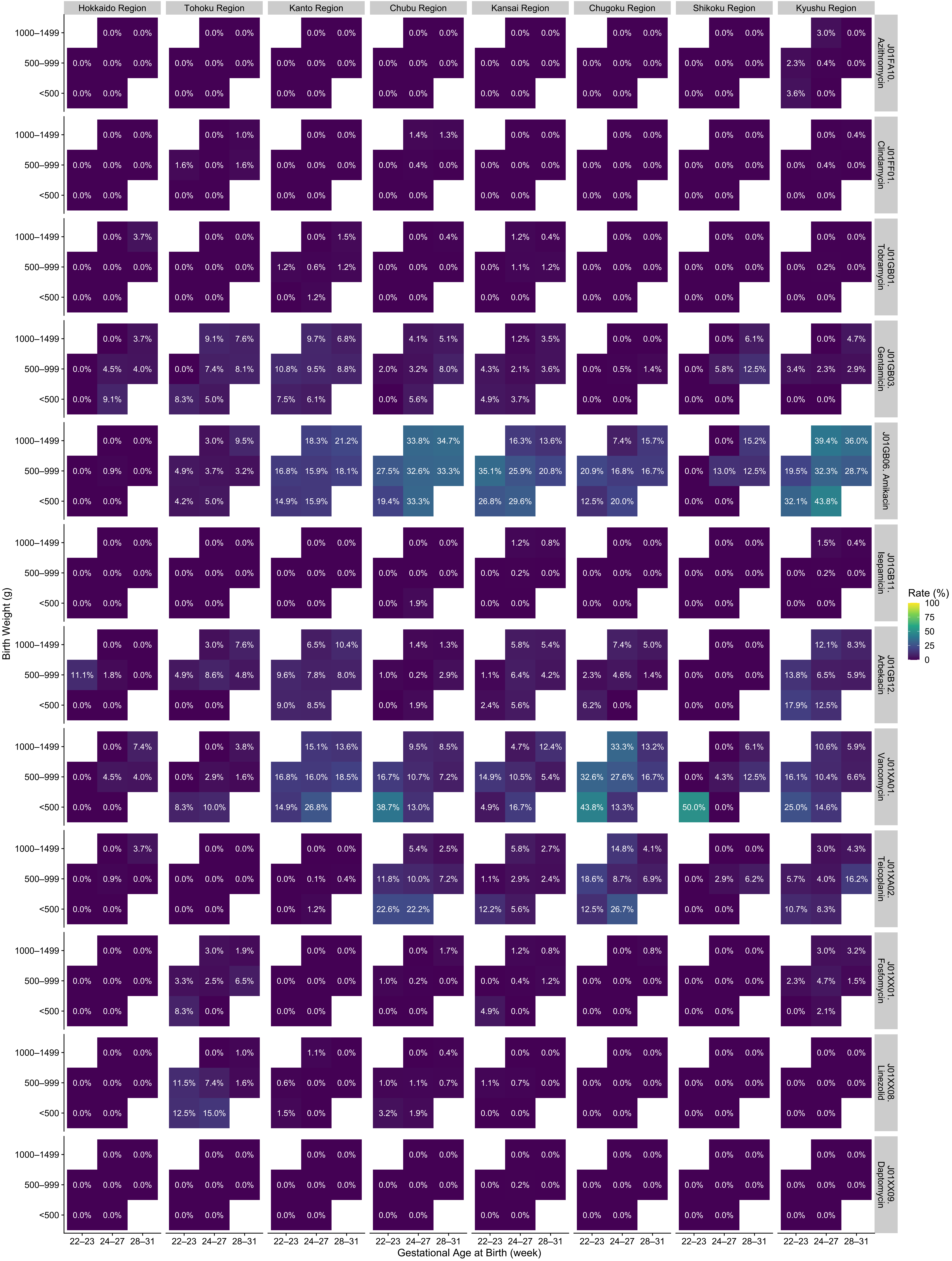

D. Early Neonatal Antimycotic Drug Exposure (Day 0–6)

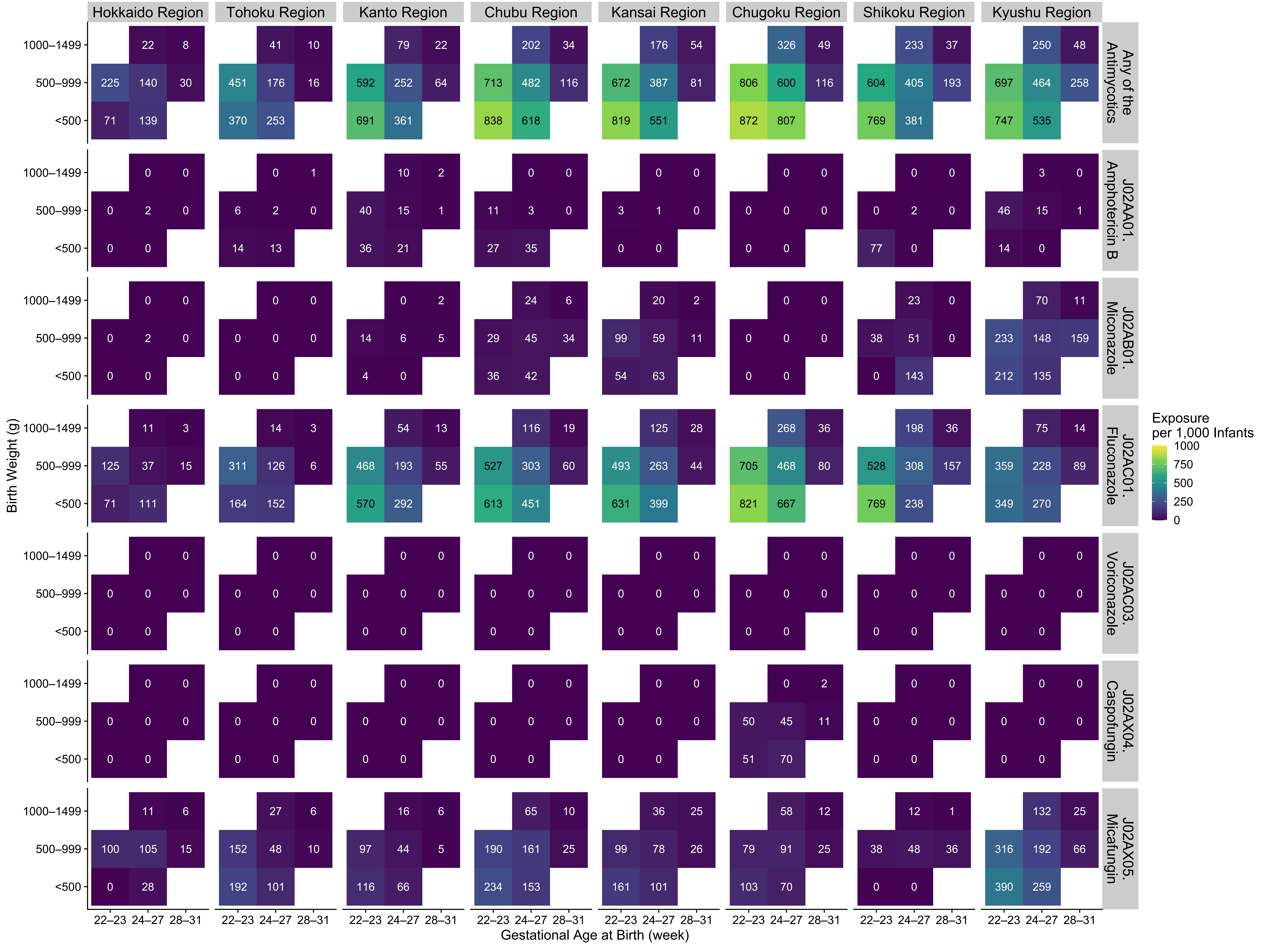

Supplement: S3 Fig — (A) Early and late neonatal episodes of antibacterial administration per 1,000 infants. (B) Drug selection rates on the first day of early neonatal courses of antibacterial administration (days 0–6). (C) Drug selection rates on the first day of late neonatal courses of antibacterial administration (days 7–27). (D) Early neonatal antimycotic drug exposure (days 0–6). We omitted the selection rate and exposure for categories with few cases, such as infants with a gestational age of 22 to 23 weeks and birth weight of 1,000 to 1,499 g and infants with a gestational age of 28 to 31 weeks and birth weight less than 500 g, because of concerns regarding high variance. (PDF) [file pone.0295528.s004.pdf]
