# Supplementary material for: Geographical distribution of antimicrobial exposure among very preterm and very low birth weight infants: A nationwide database study in Japan
Source: PLoS One. 2024 Jan 25;19(1):e0295528. doi: 10.1371/journal.pone.0295528 (PMC10810499; doi:10.1371/journal.pone.0295528)
Supplement: S1 Table — (DOCX) [file pone.0295528.s005.docx]

| **Drug Class** | **Early Neonatal Cohort** | | | **Neonatal Cohort** | | |
| --- | --- | --- | --- | --- | --- | --- |
|  | **Exposure^1^** | **Moran's *I*** | ***P* Value^2^** | **Exposure^1^** | **Moran's *I*** | ***P* Value^2^** |
| **J01AA08. Minocycline** | 0.00 | 0.00 | <0.001*** | 0.30 | -0.01 | 0.7 |
| **J01BA01. Chloramphenicol** | 0.00 | 0.00 | <0.001*** | 0.03 | -0.01 | 0.2 |
| **J01CA01. Ampicillin** | 586.63 | 0.14 | 0.076 | 598.74 | 0.12 | 0.11 |
| **J01CA12. Piperacillin** | 12.72 | 0.01 | 0.6 | 23.82 | 0.01 | 0.7 |
| **J01CA19. Aspoxicillin** | 0.02 | -0.00 | 0.019* | 0.03 | 0.00 | 0.019* |
| **J01CA51. Ampicillin, Combinations** | 55.86 | 0.09 | 0.2 | 80.28 | 0.10 | 0.13 |
| **J01CE01. Benzylpenicillin** | 0.41 | 0.11 | 0.13 | 0.43 | 0.13 | 0.076 |
| **J01CR05. Piperacillin and Beta-Lactamase Inhibitor** | 6.06 | -0.02 | 0.8 | 16.10 | -0.02 | >0.9 |
| **J01DB04. Cefazolin** | 23.27 | -0.06 | 0.4 | 62.37 | -0.08 | 0.4 |
| **J01DC07. Cefotiam** | 0.60 | 0.09 | 0.2 | 2.25 | -0.08 | 0.5 |
| **J01DC09. Cefmetazole** | 21.87 | -0.04 | 0.8 | 63.10 | 0.27 | <0.001*** |
| **J01DC14. Flomoxef** | 15.02 | -0.04 | 0.7 | 23.89 | 0.03 | 0.5 |
| **J01DD01. Cefotaxime** | 137.87 | 0.06 | 0.4 | 162.86 | 0.05 | 0.4 |
| **J01DD02. Ceftazidime** | 9.56 | -0.02 | 0.9 | 22.48 | -0.03 | 0.8 |
| **J01DD04. Ceftriaxone** | 0.17 | 0.01 | 0.7 | 0.71 | -0.06 | 0.7 |
| **J01DD62. Cefoperazone and Beta-Lactamase Inhibitor** | 0.31 | -0.05 | 0.6 | 3.10 | -0.05 | 0.6 |
| **J01DE01. Cefepime** | 0.24 | -0.08 | 0.4 | 0.96 | -0.08 | 0.5 |
| **J01DE02. Cefpirome** | 1.06 | -0.04 | 0.4 | 2.75 | -0.04 | 0.4 |
| **J01DE03. Cefozopran** | 3.89 | 0.05 | 0.2 | 11.05 | 0.09 | 0.14 |
| **J01DF01. Aztreonam** | 15.72 | 0.18 | 0.001** | 16.02 | 0.18 | 0.001** |
| **J01DH02. Meropenem** | 11.83 | 0.12 | 0.11 | 34.41 | 0.09 | 0.2 |
| **J01DH04. Doripenem** | 0.05 | -0.03 | 0.6 | 0.13 | 0.15 | <0.001*** |
| **J01DH05. Biapenem** | 0.02 | -0.03 | 0.2 | 0.33 | -0.04 | 0.3 |
| **J01DH51. Imipenem and Cilastatin** | 0.41 | 0.13 | 0.046* | 2.02 | 0.05 | 0.3 |
| **J01DH55. Panipenem and Betamipron** | 8.23 | -0.04 | 0.8 | 13.55 | -0.05 | 0.7 |
| **J01EE01. Sulfamethoxazole and Trimethoprim** | 0.00 | 0.00 | <0.001*** | 0.03 | -0.04 | 0.078 |
| **J01FA01. Erythromycin** | 15.26 | -0.09 | 0.3 | 23.04 | -0.09 | 0.3 |
| **J01FA10. Azithromycin** | 1.57 | -0.03 | 0.8 | 2.09 | 0.01 | 0.6 |
| **J01FF01. Clindamycin** | 0.29 | -0.07 | 0.5 | 1.72 | 0.05 | 0.3 |
| **J01GB01. Tobramycin** | 7.05 | 0.15 | 0.002** | 8.63 | 0.22 | <0.001*** |
| **J01GB03. Gentamicin** | 190.18 | 0.11 | 0.13 | 196.54 | 0.10 | 0.2 |
| **J01GB06. Amikacin** | 206.41 | 0.13 | 0.083 | 240.72 | 0.18 | 0.022* |
| **J01GB11. Isepamicin** | 0.14 | -0.03 | 0.8 | 0.38 | 0.02 | 0.5 |
| **J01GB12. Arbekacin** | 4.18 | 0.12 | 0.089 | 24.12 | 0.04 | 0.5 |
| **J01MA02. Ciprofloxacin** | 0.00 | 0.00 | <0.001*** | 0.03 | -0.03 | 0.6 |
| **J01MA18. Pazufloxacin** | 0.00 | 0.00 | <0.001*** | 0.03 | -0.04 | 0.078 |
| **J01XA01. Vancomycin** | 10.24 | -0.03 | >0.9 | 57.70 | 0.08 | 0.2 |
| **J01XA02. Teicoplanin** | 3.60 | -0.04 | 0.7 | 16.63 | -0.02 | >0.9 |
| **J01XD01. Metronidazole** | 0.00 | 0.00 | <0.001*** | 0.03 | -0.03 | 0.2 |
| **J01XX01. Fosfomycin** | 0.48 | 0.09 | 0.043* | 4.77 | -0.02 | >0.9 |
| **J01XX08. Linezolid** | 0.94 | -0.03 | 0.4 | 5.32 | -0.04 | 0.3 |
| **J01XX09. Daptomycin** | 0.00 | 0.00 | <0.001*** | 0.08 | -0.04 | 0.7 |
| **J02AA01. Amphotericin B** | 4.51 | -0.04 | 0.5 | 7.54 | -0.07 | 0.3 |
| **J02AB01. Miconazole** | 25.40 | 0.20 | 0.002** | 27.48 | 0.20 | 0.002** |
| **J02AC01. Fluconazole** | 126.81 | 0.18 | 0.023* | 140.20 | 0.18 | 0.027* |
| **J02AC03. Voriconazole** | 0.00 | 0.00 | <0.001*** | 0.08 | -0.07 | 0.4 |
| **J02AX04. Caspofungin** | 1.35 | -0.03 | 0.2 | 1.36 | -0.03 | 0.2 |
| **J02AX05. Micafungin** | 53.50 | -0.01 | >0.9 | 65.07 | -0.02 | >0.9 |
| **J04AC01. Isoniazid** | 0.02 | -0.02 | >0.9 | 0.03 | -0.02 | >0.9 |
| **J05AB01. Aciclovir** | 0.87 | -0.01 | 0.8 | 1.31 | 0.02 | 0.5 |
| **J05AB06. Ganciclovir** | 0.07 | -0.08 | 0.4 | 0.33 | -0.09 | 0.4 |
| **J05AH03. Peramivir** | 0.07 | -0.02 | >0.9 | 0.05 | -0.03 | 0.9 |
| ^1^Exposure per 1,000 Infants; ^2^Two-sided global Moran's I test; *p<0.05; **p<0.01; ***p<0.001; | | | | | | |
