# Supplementary material for: Geographical distribution of antimicrobial exposure among very preterm and very low birth weight infants: A nationwide database study in Japan
Source: PLoS One. 2024 Jan 25;19(1):e0295528. doi: 10.1371/journal.pone.0295528 (PMC10810499; doi:10.1371/journal.pone.0295528)
Supplement: S2 Table — (DOCX) [file pone.0295528.s006.docx]

| **Drug Class** | **Early Neonatal Cohort** | | | **Neonatal Cohort** | | |
| --- | --- | --- | --- | --- | --- | --- |
|  | **Exposure^1^** | **Moran's *I*** | ***P* Value^2^** | **Exposure^1^** | **Moran's *I*** | ***P* Value^2^** |
| **J01AA. Tetracyclines** | 0.00 | 0.00 | <0.001*** | 0.30 | -0.01 | 0.7 |
| **J01BA. Amphenicols** | 0.00 | 0.00 | <0.001*** | 0.03 | -0.01 | 0.2 |
| **J01CA. Penicillins with Extended Spectrum** | 648.53 | 0.08 | 0.3 | 666.96 | 0.06 | 0.4 |
| **J01CE. Beta-Lactamase Sensitive Penicillins** | 0.41 | 0.11 | 0.13 | 0.43 | 0.13 | 0.076 |
| **J01CR. Combinations of Penicillins, Including Beta-Lactamase Inhibitors** | 6.06 | -0.02 | 0.8 | 16.10 | -0.02 | >0.9 |
| **J01DB. First-Generation Cephalosporins** | 23.27 | -0.06 | 0.4 | 62.37 | -0.08 | 0.4 |
| **J01DC. Second-Generation Cephalosporins** | 37.47 | 0.05 | 0.3 | 88.91 | 0.27 | 0.001** |
| **J01DD. Third-Generation Cephalosporins** | 147.53 | -0.05 | 0.7 | 184.31 | -0.05 | 0.7 |
| **J01DE. Fourth-Generation Cephalosporins** | 5.19 | 0.01 | 0.7 | 14.68 | 0.01 | 0.7 |
| **J01DF. Monobactams** | 15.72 | 0.18 | 0.001** | 16.02 | 0.18 | 0.001** |
| **J01DH. Carbapenems** | 20.33 | -0.13 | 0.2 | 49.40 | -0.16 | 0.10 |
| **J01EE. Combinations of Sulfonamides and Trimethoprim, Including Derivatives** | 0.00 | 0.00 | <0.001*** | 0.03 | -0.04 | 0.078 |
| **J01FA. Macrolides** | 16.83 | -0.09 | 0.3 | 25.10 | -0.09 | 0.3 |
| **J01FF. Lincosamides** | 0.29 | -0.07 | 0.5 | 1.72 | 0.05 | 0.3 |
| **J01GB. Aminoglycosides Other than Streptomycins** | 401.23 | 0.43 | <0.001*** | 430.30 | 0.41 | <0.001*** |
| **J01MA. Fluoroquinolones** | 0.00 | 0.00 | <0.001*** | 0.05 | -0.07 | 0.5 |
| **J01XA. Glycopeptide Antibacterials** | 13.81 | 0.00 | 0.8 | 73.37 | 0.14 | 0.074 |
| **J01XD. Imidazole Derivatives** | 0.00 | 0.00 | <0.001*** | 0.03 | -0.03 | 0.2 |
| **J01XX. Other Antibacterials** | 1.42 | 0.31 | <0.001*** | 10.09 | 0.07 | 0.2 |
| **J02AA. Antibiotics** | 4.51 | -0.04 | 0.5 | 7.54 | -0.07 | 0.3 |
| **J02AB. Imidazole Derivatives** | 25.40 | 0.20 | 0.002** | 27.48 | 0.20 | 0.002** |
| **J02AC. Triazole and Tetrazole Derivatives** | 126.81 | 0.18 | 0.023* | 140.28 | 0.18 | 0.027* |
| **J02AX. Other Antimycotics for Systemic Use** | 54.85 | -0.02 | >0.9 | 66.43 | -0.03 | >0.9 |
| **J04AC. Hydrazides** | 0.02 | -0.02 | >0.9 | 0.03 | -0.02 | >0.9 |
| **J05AB. Nucleosides and Nucleotides Excluding Reverse Transcriptase Inhibitors** | 0.94 | -0.02 | >0.9 | 1.64 | -0.02 | >0.9 |
| **J05AH. Neuraminidase Inhibitors** | 0.07 | -0.02 | >0.9 | 0.05 | -0.03 | 0.9 |
| ^1^Exposure per 1,000 Infants; ^2^Two-sided global Moran's I test; *p<0.05; **p<0.01; ***p<0.001; | | | | | | |
