# Supplementary material for: Geographical distribution of antimicrobial exposure among very preterm and very low birth weight infants: A nationwide database study in Japan
Source: PLoS One. 2024 Jan 25;19(1):e0295528. doi: 10.1371/journal.pone.0295528 (PMC10810499; doi:10.1371/journal.pone.0295528)
Supplement: S3 Table — (DOCX) [file pone.0295528.s007.docx]

| **Drug Class** | **Early Neonatal Cohort** | | | **Neonatal Cohort** | | |
| --- | --- | --- | --- | --- | --- | --- |
|  | **Exposure^1^** | **Moran's *I*** | ***P* Value^2^** | **Exposure^1^** | **Moran's *I*** | ***P* Value^2^** |
| **J01AA08. Minocycline** | 0.00 | 0.00 | <0.001*** | 0.50 | -0.02 | >0.9 |
| **J01BA01. Chloramphenicol** | 0.00 | 0.00 | <0.001*** | 0.05 | -0.01 | 0.2 |
| **J01CA01. Ampicillin** | 640.00 | 0.11 | 0.14 | 657.05 | 0.09 | 0.2 |
| **J01CA12. Piperacillin** | 13.85 | -0.00 | 0.7 | 30.56 | 0.00 | 0.7 |
| **J01CA51. Ampicillin, Combinations** | 61.81 | 0.06 | 0.3 | 98.85 | 0.06 | 0.3 |
| **J01CE01. Benzylpenicillin** | 0.33 | 0.14 | 0.048* | 0.40 | 0.18 | 0.015* |
| **J01CR05. Piperacillin and Beta-Lactamase Inhibitor** | 9.12 | -0.02 | >0.9 | 24.62 | -0.02 | >0.9 |
| **J01DB04. Cefazolin** | 30.81 | -0.06 | 0.4 | 92.32 | -0.07 | 0.6 |
| **J01DC07. Cefotiam** | 0.43 | 0.06 | 0.2 | 2.89 | -0.09 | 0.4 |
| **J01DC09. Cefmetazole** | 29.91 | -0.02 | >0.9 | 93.02 | 0.28 | <0.001*** |
| **J01DC14. Flomoxef** | 15.78 | -0.05 | 0.6 | 29.21 | 0.04 | 0.5 |
| **J01DD01. Cefotaxime** | 154.58 | 0.04 | 0.5 | 190.22 | 0.04 | 0.5 |
| **J01DD02. Ceftazidime** | 11.48 | -0.02 | 0.9 | 33.05 | -0.03 | 0.7 |
| **J01DD04. Ceftriaxone** | 0.19 | -0.02 | >0.9 | 0.80 | -0.06 | 0.6 |
| **J01DD62. Cefoperazone and Beta-Lactamase Inhibitor** | 0.57 | -0.05 | 0.7 | 5.03 | -0.05 | 0.6 |
| **J01DE01. Cefepime** | 0.24 | -0.06 | 0.6 | 1.45 | -0.09 | 0.4 |
| **J01DE02. Cefpirome** | 1.65 | -0.04 | 0.4 | 4.59 | -0.04 | 0.4 |
| **J01DE03. Cefozopran** | 5.77 | 0.02 | 0.6 | 17.10 | 0.04 | 0.4 |
| **J01DF01. Aztreonam** | 21.03 | 0.20 | 0.001** | 21.29 | 0.20 | 0.001** |
| **J01DH02. Meropenem** | 18.38 | 0.04 | 0.5 | 57.18 | 0.07 | 0.3 |
| **J01DH04. Doripenem** | 0.09 | -0.03 | 0.6 | 0.20 | -0.03 | 0.6 |
| **J01DH05. Biapenem** | 0.05 | -0.03 | 0.2 | 0.55 | -0.04 | 0.3 |
| **J01DH51. Imipenem and Cilastatin** | 0.57 | 0.12 | 0.047* | 2.74 | 0.06 | 0.3 |
| **J01DH55. Panipenem and Betamipron** | 12.29 | -0.02 | >0.9 | 21.29 | -0.05 | 0.7 |
| **J01EE01. Sulfamethoxazole and Trimethoprim** | 0.00 | 0.00 | <0.001*** | 0.05 | -0.04 | 0.078 |
| **J01FA01. Erythromycin** | 21.22 | -0.09 | 0.3 | 31.80 | -0.09 | 0.3 |
| **J01FA10. Azithromycin** | 2.65 | -0.04 | 0.8 | 3.44 | 0.02 | 0.5 |
| **J01FF01. Clindamycin** | 0.38 | -0.07 | 0.5 | 2.69 | 0.03 | 0.5 |
| **J01GB01. Tobramycin** | 6.99 | 0.22 | <0.001*** | 9.12 | 0.29 | <0.001*** |
| **J01GB03. Gentamicin** | 216.01 | 0.08 | 0.3 | 225.16 | 0.07 | 0.3 |
| **J01GB06. Amikacin** | 245.36 | 0.09 | 0.2 | 301.23 | 0.15 | 0.060 |
| **J01GB11. Isepamicin** | 0.19 | -0.02 | 0.6 | 0.40 | -0.05 | 0.7 |
| **J01GB12. Arbekacin** | 7.14 | 0.14 | 0.047* | 36.84 | 0.03 | 0.5 |
| **J01MA02. Ciprofloxacin** | 0.00 | 0.00 | <0.001*** | 0.05 | -0.03 | 0.6 |
| **J01MA18. Pazufloxacin** | 0.00 | 0.00 | <0.001*** | 0.05 | -0.04 | 0.078 |
| **J01XA01. Vancomycin** | 16.49 | -0.03 | >0.9 | 92.77 | 0.05 | 0.4 |
| **J01XA02. Teicoplanin** | 6.38 | -0.03 | >0.9 | 27.96 | -0.02 | >0.9 |
| **J01XD01. Metronidazole** | 0.00 | 0.00 | <0.001*** | 0.05 | -0.03 | 0.2 |
| **J01XX01. Fosfomycin** | 0.85 | 0.06 | 0.046* | 7.78 | -0.02 | >0.9 |
| **J01XX08. Linezolid** | 1.61 | -0.03 | 0.4 | 9.92 | -0.04 | 0.4 |
| **J01XX09. Daptomycin** | 0.00 | 0.00 | <0.001*** | 0.10 | -0.03 | 0.2 |
| **J02AA01. Amphotericin B** | 7.99 | -0.05 | 0.5 | 13.66 | -0.08 | 0.3 |
| **J02AB01. Miconazole** | 44.80 | 0.20 | 0.002** | 48.70 | 0.19 | 0.003** |
| **J02AC01. Fluconazole** | 224.28 | 0.18 | 0.025* | 247.84 | 0.18 | 0.025* |
| **J02AC03. Voriconazole** | 0.00 | 0.00 | <0.001*** | 0.10 | -0.06 | 0.6 |
| **J02AX04. Caspofungin** | 2.55 | -0.03 | 0.2 | 2.59 | -0.03 | 0.2 |
| **J02AX05. Micafungin** | 88.42 | -0.03 | >0.9 | 108.37 | -0.05 | 0.8 |
| **J04AC01. Isoniazid** | 0.05 | -0.02 | >0.9 | 0.05 | -0.02 | >0.9 |
| **J05AB01. Aciclovir** | 0.71 | -0.13 | 0.2 | 1.25 | -0.07 | 0.5 |
| **J05AB06. Ganciclovir** | 0.05 | -0.03 | 0.2 | 0.55 | -0.06 | 0.6 |
| **J05AH03. Peramivir** | 0.09 | -0.03 | 0.9 | 0.05 | -0.03 | 0.6 |
| ^1^Exposure per 1,000 Infants; ^2^Two-sided global Moran's I test; *p<0.05; **p<0.01; ***p<0.001; | | | | | | |
