# Supplementary material for: Geographical distribution of antimicrobial exposure among very preterm and very low birth weight infants: A nationwide database study in Japan
Source: PLoS One. 2024 Jan 25;19(1):e0295528. doi: 10.1371/journal.pone.0295528 (PMC10810499; doi:10.1371/journal.pone.0295528)
Supplement: Attachment — Submitted filename: point_by_point_response_to_editor_and_reviewers.docx [file pone.0295528.s008.docx]

**Our point-by-point response to all comments and suggestions is listed below:**

We thank you for taking the time and effort necessary to review our manuscript and provide us with these valuable comments and suggestions. Accordingly, we revised our manuscript and made changes to it.

Journal requirements #1:

Please ensure that your manuscript meets PLOS ONE's style requirements, including those for file naming. The PLOS ONE style templates can be found at

<https://journals.plos.org/plosone/s/file?id=wjVg/PLOSOne_formatting_sample_main_body.pdf> and

<https://journals.plos.org/plosone/s/file?id=ba62/PLOSOne_formatting_sample_title_authors_affiliations.pdf>

Response:

We thank you for the comment. We checked the style requirements.

---

Journal requirements #2:

In your Data Availability statement, you have not specified where the minimal data set underlying the results described in your manuscript can be found. PLOS defines a study's minimal data set as the underlying data used to reach the conclusions drawn in the manuscript and any additional data required to replicate the reported study findings in their entirety. All PLOS journals require that the minimal data set be made fully available. For more information about our data policy, please see <http://journals.plos.org/plosone/s/data-availability>.

Response:

We thank you for your comment. We have specified our contact information for accessing the data in the manuscript, referring to the "acceptable data access restrictions" section of the linked page and similar previous studies (*Nagano, Hiroyuki, et. al., 2021. “Hospitalization for Ischemic Stroke Was Affected More in Independent Cases than in Dependent Cases during the COVID-19 Pandemic: An Interrupted Time Series Analysis.” PloS One 16 (12): e0261587., Ito, Fumiya, et. al., 2023. “Validation Study on Definition of Cause of Death in Japanese Claims Data.” PloS One 18 (3): e0283209.*).

Page 1, lines 14–17 (Title page)

***Data Availability****: Data cannot be disclosed to the public due to a license agreement and ethical issues in each participating facility. For data sharing of the dataset generated during this study, please contact the corresponding author or the Office of Life Science and Bioethics Research Center (Email: infobec@tmd.ac.jp; Tel: +81-3-3813-6111).*

---

Journal requirements #3:

*We note that Figures 2, 3, S1 Fig and S2 Fig in your submission contain [map/satellite] images which may be copyrighted. All PLOS content is published under the Creative Commons Attribution License (CC BY 4.0), which means that the manuscript, images, and Supporting Information files will be freely available online, and any third party is permitted to access, download, copy, distribute, and use these materials in any way, even commercially, with proper attribution. For these reasons, we cannot publish previously copyrighted maps or satellite images created using proprietary data, such as Google software (Google Maps, Street View, and Earth). For more information, see our copyright guidelines:* [*http://journals.plos.org/plosone/s/licenses-and-copyright*](http://journals.plos.org/plosone/s/licenses-and-copyright)*.*

Response:

We have obtained written permission from the Ministry of Land, Infrastructure, Transport and Tourism to use content under the CC BY 4.0 license. Please check the uploaded document.

---

Journal requirements #4:

*Please review your reference list to ensure that it is complete and correct. If you have cited papers that have been retracted, please include the rationale for doing so in the manuscript text, or remove these references and replace them with relevant current references. Any changes to the reference list should be mentioned in the rebuttal letter that accompanies your revised manuscript. If you need to cite a retracted article, indicate the article’s retracted status in the References list and also include a citation and full reference for the retraction notice.*

Response:

We thank you for the comment. Accordingly, we checked the reference list.

---

Editors’ Comments #1:

*On the short title: correct the spelling error(wight) .. replace it with weight.*

Response:

We thank you for pointing this out. Accordingly, we corrected the spelling.

Page 2, lines 24–25 (Title page)

Geographical distribution of antimicrobial exposure among very preterm and very low birth *weight* infants in Japan

---

Editor Comments #2:

*Please delete- "Compared to the results of previous studies in the United States, exposures to cefotaxime and gentamicin were higher and lower in Japan, respectively." in the abstract section. You are not supposed to discuss results in the abstract section.*

Response:

We thank you for this suggestion. Accordingly, we deleted the sentence.

---

Editors comment #3:

Please check all the references once again and shall be as per PLOS ONE guideline(e.g. put the "." after the intext citation).

Response:

We thank you for the suggestion. Accordingly, we checked the references and revised the in-text citations.

---

Editors’ comments #4:

*Please cite the source of the late neonatal period definition. Also please, operationally define the early neonatal and neonatal cohort in the method section.*

Response:

We thank you for the suggestion. We added the citation for the source of the definition (*Oza S, Lawn JE, Hogan DR, Mathers C, Cousens SN. Neonatal cause-of-death estimates for the early and late neonatal periods for 194 countries: 2000-2013. Bull World Health Organ. 2015;93: 19–28*). The original sentence “Infants discharged during the corresponding periods were excluded from the analysis." might have been unclear. Therefore, we elaborated on the description for clarity, as indicated below.

Page 5, lines 79–83, (Methods)

*We defined two specific cohorts based on the discharge period of infants. Cases discharged during the early neonatal period were excluded. Consequently, we established an “early neonatal cohort” to specifically assess drug exposure during this phase. Similarly, upon the exclusion of cases discharged during the neonatal period, we delineated a separate “neonatal cohort” for further analysis.*

---

Editors’ comments #5:

How do you define antimicrobial exposure (single dose vs multiple doses for prolonged time)? Was the study addressed duration? If not it shall be addressed as a study limitation.

Response:

We thank you for these queries. As noted in the method section, no distinction was made between single and multiple doses. The effect of cumulative drug exposure is a very important topic. However, susceptibility to adverse effects could vary widely based on genetic background, postnatal age, and postmenstrual age. In this study, we focused on identifying injectable antimicrobials prescribed for many infants. We added the text below to the limitation part.

Page 12–13, lines 230–232 (Limitation)

*Our study focused on identifying and analyzing drugs to which a notable proportion of infants were exposed at least once, and we did not evaluate the duration or total dosage of drug exposure.*

---

Editors’ comment #6:

*As a limitation, clinical indications were not collected and analyzed. But on the conclusion, the geographical variation might be because of the clinical hetrogeniety. How do you come up with such conclusion. Can you please clarify?*

Response:

We thank you for the suggestion. To avoid misunderstanding, the term “clinical heterogeneity” has been changed to “geographical heterogeneity” throughout the entire manuscript, including the following. The term “clinical heterogeneity” was intended to indicate heterogeneity in clinical *care*, not in the clinical *backgrounds*. Regional differences in the detailed treatment indication have not been verified. However, we have revealed the regional variations in clinical care of antimicrobial administration.

Page 13, lines 251–253 (Conclusion)

Our study uncovered …, thereby revealing spatial autocorrelation and *geographical* heterogeneity.

---

Reviewer #1’s comments #1:

*All readers are not familiar with Global and local Moran’s I statistics. Although those might be basic things for statisticians, it would be better for the authors to have an additional explanation about what it means by high-high, high-low, low-low, and low-high in the selection of antimicrobials.*

Response:

We thank you for the comment. We have revised the description as follows.

Page 5, lines 97–106 (Methods)

A high-high classification indicates “high exposure surrounded by high exposures.” This is indicative of a positive spatial autocorrelation or a spatial cluster of high exposures (a hot spot). A high-low classification is an outliner that indicates “high exposure in the target district while neighboring districts had low exposure.” A low-high classification is another outliner that indicates “low exposure in the target district while neighboring districts had high exposure.” A low-low classification indicates “low exposure surrounded by low exposures”. Similar to the “high-high” scenario, this is indicative of positive spatial autocorrelation or a spatial cluster of low exposures (a cold spot). The “not significant” classification indicates a lack of either positive or negative significant spatial autocorrelation according to the local Moran’s *I* statistics.

---

Reviewer #1’s comments #2:

*I am not sure that those statistical methods are appropriate for the explanation of the outcomes of preterm infants in Japan. Do the authors have any information of morbidity or mortality especially in Hokkaido or Kyushu area that might be different from other areas, because those regional differences in antimicrobial strategy might affect those outcomes?*

Response:

We thank you for the comment. As shown below, we have performed supplementary spatial analyses on early neonatal and infant mortalities in 2020 among very low birth weight infants in each prefecture, based on government statistics (https://www.e-stat.go.jp/en/node). No obvious spatial effects were identified. We are currently considering a causal inferential analysis based on linkage with a nationwide case registry. If required, we would add these figures to the supplementary materials.

**Fig. The early neonatal mortality and infant mortality among VLBW infants based on the Japanese government statistics.**

---

Reviewer #2’s comments #1:

*Results need modification of Figure 2 because is blurred, and Figure 3 is very blurred.*

Response:

Thank you for the suggestion. The resolution of the raster image initially submitted was the maximum within the regulations. For better visibility, we created and uploaded these figures in EPS format.

---

Along with including the DOIs in the reference, the subsequent modifications were made:

Additional Corrections #1

We have corrected misstatements of the study period.

Page 3, line 33 (Abstract) and Page 5, lines 77 (Methods)

Before: from April 2012 to March 2021

After: from April 2010 to March 2021

---

Additional Corrections #2

In February 2023, the count of islands in Japan has changed due to enhancements in map precision, and we have reflected this accordingly.

https://www.gsi.go.jp/kihonjohochousa/pressrelease20230228.html

https://www.nippon.com/en/japan-data/h01615/

Page 12, line 213 (Discussion)

Before: (…) that comprise more than 2,500 islands [30].

After: (…) that comprise 4,600 islands [30].
